# Climate change linked to functional homogenization of a subtropical estuarine system

**DOI:** 10.1002/ece3.8783

**Published:** 2022-04-12

**Authors:** Michaela Pawluk, Masami Fujiwara, Fernando Martinez‐Andrade

**Affiliations:** ^1^ 14736 Department of Wildlife and Fisheries Sciences Texas A&M University College Station Texas USA; ^2^ 14736 Department of Ecology and Conservation Biology Texas A&M University College Station Texas USA; ^3^ 114669 Coastal Fisheries Division Texas Parks and Wildlife Department Corpus Christi Texas USA

**Keywords:** climate change, fish community dynamics, functional diversity

## Abstract

Climate change causes marine species to shift and expand their distributions, often leading to changes in species diversity. While increased biodiversity is often assumed to confer positive benefits on ecosystem functioning, many examples have shown that the relationship is specific to the ecosystem and function studied and is often driven by functional composition and diversity. In the northwestern Gulf of Mexico, tropical species expansion was shown to have increased estuarine fish and invertebrate diversity; however, it is not yet known how those increases have affected functional diversity. To address this knowledge gap, two metrics of functional diversity, functional richness (FRic) and functional dispersion (FDis), were estimated in each year for a 38‐year study period, for each of the eight major bays along the Texas coast. Then, the community‐weighted mean (CWM) trait values for each of the functional traits are calculated to assess how functional composition has changed through time. Finally, principal component analysis (PCA) was used to identify species contributing most to changing functional diversity. We found significant increases in log‐functional richness in both spring and fall, and significant decreases in functional dispersion in spring, suggesting that although new functional types are entering the bays, assemblages are becoming more dominated by similar functional types. Community‐weighted trait means showed significant increases in the relative abundance of traits associated with large, long‐lived, higher trophic level species, suggesting an increase in periodic and equilibrium life‐history strategists within the bays. PCA identified mainly native sciaenid species as contributing most to functional diversity trends although several tropical species also show increasing trends through time. We conclude that the climate‐driven species expansion in the northwestern Gulf of Mexico led to a decrease in functional dispersion due to increasing relative abundance of species with similar life‐history characteristics, and thus the communities have become more functionally homogeneous.

## Graphical abstract

Distribution shifts in fish species, which have been linked to increasing temperatures, have led to subsequent increases in fish species diversity. In our study, increasing species diversity led to increased functional richness but decreased functional dispersion. Increasing prevalence of large, long‐lived, predatory species has resulted in functional homogenization of the fish assemblages.

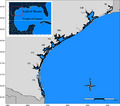

## 1 INTRODUCTION

Biodiversity is often associated with positive effects on ecosystem functions (Loreau, 1998); however, depending on the specific function being considered, the relationship between species diversity and ecosystem functioning can be positive, neutral, or even negative (Schwartz et al., 2000). Evidence suggests that ecosystem functioning is more closely related to functional diversity (Hooper et al., 2005), and thus the observed relationship between biodiversity and ecosystem function is driven by the relationship between biodiversity and functional diversity for a given ecosystem and function (Mayfield et al., 2010). For ecosystems in which functional diversity and species diversity are positively correlated, ecosystem functioning is expected to increase with increasing taxonomic diversity (Petchey, 2000). Understanding the relationship between taxonomic and functional diversity is, thus, critical for predicting the potential consequences of anthropogenic impacts to ecosystems.

In marine ecosystems, climate change is driving large‐scale expansion and distribution shifts for many species (Sorte et al., 2010). For example, Perry et al. (2005) found that increasing sea temperatures over a 25‐year period in the North Sea led to increasing mean latitude of occurrence, increasing depth of occurrence, or both, for nearly two‐thirds of the fish species observed. While a meta‐analysis of coastal survey data by Pinsky et al. (2013) found that climate velocity (the rate and direction of climate shifts) significantly explained fish and invertebrate distribution shifts. Many such distribution shifts and expansions have led to increasing fish species diversity in temperate and subtropical ecosystems (Fujiwara et al., 2019; Hiddink & Ter Hofstede, 2008; Pawluk et al., 2021). As novel communities are formed due to range shifts, novel interactions between native and invading species are potentially produced (Van der Putten et al., 2010). It is, therefore, imperative to understand how changing diversity due to species range shifts or expansions relates to changing functional diversity, in order to understand how climate change will impact ecosystem functioning of marine ecosystems. It is also important to identify how expanding species may impact the dynamics of native species and drive trends in changing functional composition and diversity.

While previous studies have demonstrated evidence of climate‐induced distribution shifts (Collie et al., 2008; Perry et al., 2005; Pinsky et al., 2013) and increasing taxonomic diversity of marine fish species (Stefansdottir et al., 2010), few studies to date have addressed impacts of climate‐driven marine distribution shifts on functional composition and diversity (McLean et al., 2018, 2019). In this study, we address this knowledge gap by assessing the impact of expanding tropical species on fish functional diversity in the bays in the northwestern part of the Gulf of Mexico. Previous studies have indicated that fish and invertebrate diversity in the bays has increased during recent years due to the expansion of tropical species associated with increasing temperatures and salinities (Fujiwara et al., 2019; Pawluk et al., 2021). However, it is not yet known how such expansion has affected the functional composition and diversity.

By analyzing a 38‐year survey dataset from the bays of Texas, we assess the long‐term trends in functional diversity and functional composition of a subtropical coastal ecosystem. In this study, we consider functional diversity to be the “components of biodiversity that influence how an ecosystem operates or functions” (Tilman, 2001); therefore, a broad range of traits are considered in the analysis. A variety of metrics are proposed for characterizing the functional diversity and structure of a community (Cadotte et al., 2011), with trait‐based approaches often being used for characterization of functional diversity without explicit a priori grouping of species (Coleman et al., 2015; Laliberté & Legendre, 2010; Silva‐Júnior et al., 2017; Villéger et al., 2010). Trait‐based approaches allow for the incorporation of life‐history characteristics (e.g., age at maturation, maximum size), trophic characteristics (e.g., diet type, trophic level, feeding mode), and habitat characteristics (e.g., water column position, salinity preference, temperature preference), which allows for a more detailed characterization of a species position within the community, without the restrictive nature of broad functional groups. Two such trait‐based metrics frequently used to characterize functional diversity include functional richness (FRic) and functional dispersion (FDis).

Functional richness is given by the minimum convex hull volume for a multidimensional trait space and measures the range of trait values along each trait axis, with higher functional richness implying more diverse trait types within the assemblage (Villéger et al., 2008). Whereas FRic is informative as to the volume of trait space occupied, it does not incorporate relative abundances of trait values. Functional dispersion measures the abundance‐weighted mean distance to the abundance‐weighted centroid in trait space (Laliberté & Legendre, 2010), and because it accounts for the relative abundance of different trait types, it is informative on whether a community is dominated by a particular functional type, or by a variety of diverse functional types. Combining the two metrics allows for a determination of whether or not trait space has expanded or contracted, and whether any changes to trait space are significantly impacting the functioning of the system (i.e., whether new traits are occurring in high relative abundance).

Although these indices provide some indication of how functional diversity has changed through time, they do not provide insight into which functional types have contributed to the observed changes, or how individual traits may be changing through time. To address this issue, community‐weighted trait means are used. Essentially, the mean value of each trait, weighted by the relative abundance of each species, across all species within the community is calculated (Lavorel et al., 2008). By calculating traits means in each year, temporal trends in trait values can be inferred, allowing for interpretation of how functional composition has changed.

Using a trait‐based approach, we seek to address three main questions: (1) *Has increasing species diversity led to increasing functional diversity?* (2) *How has functional composition changed through time in response to species expansion?* (3) *Which species are contributing most to trends in functional diversity and composition?* We address these questions in three steps: (1) calculation of functional diversity indices for the bays of Texas in each year and season, (2) calculation of community‐weighted trait means to assess changes to functional structure, and (3) ordination of species abundances to identify those species contributing the most to observed trends.

## 2 METHODS

### 2.1 Data collection

**FIGURE 1:**
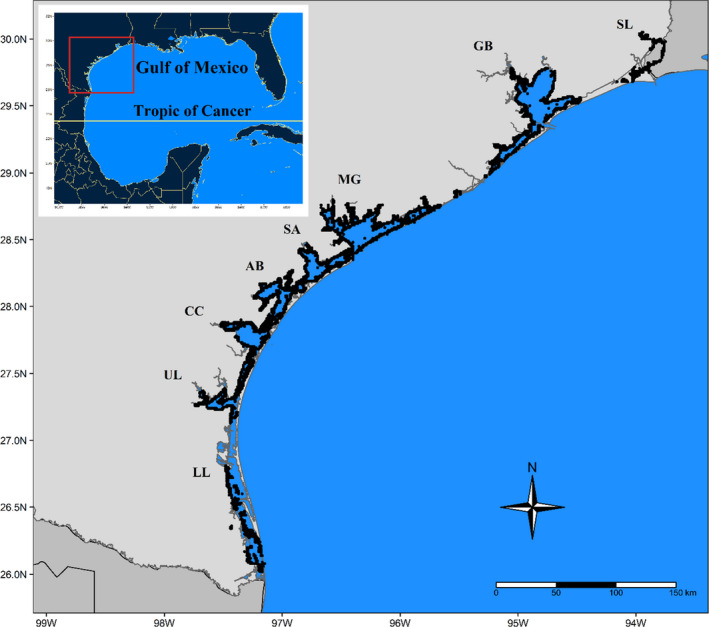
Map of gillnet sample locations. The map depicts the Texas coastline with sample locations shown as black dots. An inset map shows the entire Gulf of Mexico, with the Tropic of Cancer separating the gulf into tropical and subtropical regions. Each bay is labeled with its corresponding bay abbreviation: Sabine Lake (SL), Galveston Bay (GB), Matagorda Bay (MG), San Antonio Bay (SA), Aransas Bay (AB), Corpus Christi Bay (CC), Upper Laguna Madre (UL), and Lower Laguna Madre (LL)

The abundance data for this project were collected over a 38‐year period from 1982 to 2019 as a part of a gillnet survey program conducted by the Coastal Fisheries Division of the Texas Parks and Wildlife Department (TPWD) (Martinez‐Andrade, 2015). Samples were collected in spring (April–June) and fall (September–November) from each of the eight major bays of Texas: Sabine Lake, Galveston Bay, Matagorda Bay, San Antonio Bay, Aransas Bay, Corpus Christi Bay, Upper Laguna Madre, and Lower Laguna Madre (Figure 1). For Sabine Lake, sampling did not begin until 1986, while the other seven bays have been consistently sampled since 1982. In all sampled seasons, a total of 45 gillnet samples were collected from each bay. For determining the location of each sample, the bays were divided into a 1‐minute latitude by a 1‐minute longitude sample grid, with each grid square being further divided into 144 gridlets of 5‐second latitude by 5‐second longitude. A stratified cluster sampling design was used to randomly select individual grid square locations without replacement from the predefined sample grid for each bay, and gridlet locations within the selected grid square for net placement. Gillnets comprised four equal length (45.7 m) panels of differing mesh sizes (76 mm, 102 mm, 127 mm, and 152 mm). Gillnets were set perpendicular to the shoreline, with the smallest mesh size nearest to the shore, and allowed to soak from sunset to sunrise for an average of 13.5 h. For all samples, each individual caught was identified to species, and concurrent latitude, longitude, and environmental data were recorded. The environmental data consist of temperature (Celsius), Salinity, dissolved oxygen (ppm), and turbidity (NTU). Then, the data were converted into the catch per unit effort (number of individuals caught per hour; CPUE) for each species.

In addition to species catch per unit effort (CPUE), functional trait data were obtained from existing sources for each species observed. Life history and trait data were collected from the database FishBase (Froese & Pauly, 2020), and from the data‐integrated predictive life history model developed by Thorson et al. (2017) available in the R package “FishLife” (R Core Team, 2018; Thorson, 2019). The “FishLife” package allows for estimation of life‐history parameters by incorporating existing data, life‐history correlations, and similarities among related species, into a multivariate random‐walk model. The functional traits used in this study are shown in Table 1. These traits were selected to represent aspects of life history, diet, and niche utilization. All non‐numeric traits were converted to numerical traits, following an ordinal scale, in order to simplify the calculation of functional diversity indices and mean trait values, as many analyses do not allow for mixed data types, as well as to improve interpretability for categorical trait means. It is important to note that using Gower's dissimilarity would allow for the calculation of functional diversity indices with mixed data types; however, the mean trait values for categorical variables would then be calculated as the most commonly occurring trait value. We, thus, used the conversion to numerical traits in order to be able to track how relative abundances of categorical values have changed through time, rather than simply determining the most common category in each year.

**TABLE 1:** Fish functional traits. Trait abbreviations, descriptions, units (or scale for categorical variables), and the data source for all functional traits included in the analysis. FB: FishBase species page. For traits with multiple sources, the sources are listed in order of preference (i.e., if info is not available from source 1 it is taken from source 2, then 3 etc.). When trait data were not available from any of the listed sources the cell was left blank

Trait abbreviation	Trait description	Units or scale	Data source
Lmax	Maximum observed length (TL)	mm	FB
Lcom	Commonly observed length (TL)	mm	FB
Lmat	Length at maturity (TL)	mm	(1) FB life‐history tool, (2) R FishLife
Amat	Age at maturity	yrs	(1) FB life‐history tool, (2) R FishLife
Tmax	Maximum reported age	yrs	(1) FB, (2) FB life‐history tool, (3) R FishLife
Wmax	Maximum reported weight	kg	(1) FB, (2) FB life‐history tool, (3) R FishLife
Linf	L infinity, von Bertalanffy asymptotic length	mm	(1) FB life‐history tool, (2) R FishLife
K	von Bertalanffy growth parameter	unitless	(1) FB life‐history tool, (2) R FishLife
t0	von Bertalanffy hypothetical age at length‐0	yrs	(1) FB life‐history tool, (2) R FishLife
M	Natural mortality rate	unitless	(1) FB life‐history tool, (2) R FishLife
GenT	Generation time	yrs	(1) FB life‐history tool, (2) R FishLife
TrLvl	Trophic level	unitless	(1) FB, (2) R FishLife
MoPos	Mouth position	1(inferior), 2(subterminal), 3(terminal), 4(supraterminal), 5(superior)	Visual assessment
CauFin	Caudal fin shape	0(reduced), 1(rounded), 2(truncate), 3(emarginate), 4(lunate), 5(forked), 6(heterocercal)	Visual assessment
BdSh	Body shape	1(flat ‐ dorsoventrally), 2(elongate ‐ long and narrow), 3(moderate, fusiform), 4(deep ‐ dorsoventrally)	Visual assessment
CrossSec	Body cross‐section	1(depressed ‐ dorsoventral compression), 2(round), 3(oval), 4(compressed ‐ lateral)	Visual assessment
piscivore	Consumes fish	1(Yes), 0(No)	FB
invertivore	Consumes invertebrates	1(Yes), 0(No)	FB
herbivore	Consumes algae or other plants	1(Yes), 0(No)	FB
detritivore	Consumes detritus	1(Yes), 0(No)	FB
Pos	Water column position	1(Near‐shore/reef associated), 2(pelagic‐neritic), 3(pelagic‐oceanic), 4(benthopelagic), 5(demersal)	FB
RepGuild	Parental care (based on reproductive guild)	0(open water/substratum egg scatterers), 1(brood hiders), 2(nest guarders), 3(clutch tenders), 4(external brooders), 5(internal live bearers)	FB
Temp	Preferred temperature, mean temperature of occurrence	Celsius	(1) FB, (2) R FishLife
MinTemp	Minimum temperature in which species is observed	Celsius	FB
MaxTemp	Maximum temperature in which species is observed	Celsius	FB
TempRng	Temperature range (Maximum – Minimum)	Celsius	Calculated from other traits
Sal	Salinity preference	0(freshwater), 1(freshwater/brackish), 2(freshwater/marine/brackish), 3(marine/brackish), 4(marine)	FB

### 2.2 Functional diversity indices

In order to assess the functional impact of tropical species expansion into the bays of Texas, estimates of two functional diversity indices were calculated for each bay and year for both spring and fall assemblages. Functional richness (FRic) provides a measure of the volume of trait space occupied by a community, with higher FRic implying a wider range of trait values along one or more trait axes (Villéger et al., 2008). While functional richness can provide insight into the range of functional types within a community, it does not incorporate relative abundance of different trait types. Functional dispersion (FDis) addresses this issue by incorporating species relative abundance and their position in trait space to determine how clumped or dispersed community abundance is within the occupied trait space (Laliberté & Legendre, 2010). Prior to calculating the functional diversity indices, species that were encountered fewer than three times throughout the study period were removed from the dataset to limit the influence of very rare species. Calculations for FRic and FDis were done using the dbFD function within the “FD” package in R (Laliberté et al., 2014; R Core Team, 2018). This function uses Gower's dissimilarity as opposed to Euclidean distance in calculating the FD indices when there are missing data, as in our case. Additionally, a correction method must be specified when distances cannot be represented as Euclidean distances for use in the principal coordinates analysis (PCoA) needed for calculating the indices. In our case, the “lingoes” correction was employed (Lingoes, 1971).

In order to assess the significance of any temporal trends in FRic and FDis, linear models were fit for each bay and season, with the metric as the dependent variable and year as the independent variable. For FRic, the natural log of FRic was used as the response variable to stabilize the variance. To assess spatial and seasonal differences in functional diversity, analysis of variance (ANOVA) was used with the following equation: metric ~bay + season, where metric is either logFRic or FDis, bay is a categorical variable distinguishing between bays (arranged from north to south, with Sabine Lake being the northernmost bay and Lower Laguna Madre being the southernmost bay), and season being a categorical variable, either spring or fall.

### 2.3 Analysis of trait means

Calculation of functional diversity metrics is informative as to whether a community has become more or less functionally diverse but provides no information on which traits predominate within the community, or how traits may be changing through time. In order to characterize the functional composition and assess the significance of temporal trends in trait changes, community‐weighted mean (CWM) trait values were calculated for all functional traits in each year and bay for both spring and fall assemblages. Community‐weighted trait means are calculated as the mean trait value for all species within the community, weighted by species abundance (i.e., more abundant species have stronger influence on the mean trait value). Abundance‐weighted trait means were calculated using the “functcomp” function within the “FD” package (Laliberté et al., 2014) in R, which takes a species by trait matrix, and site by species abundance matrix and returns the community‐level weighted mean for each trait within the trait matrix at each site. For the purposes of this analysis, “sites” were a given year within a given bay, for each season.

After obtaining a time series of CWM trait values for each bay and season, it was possible to statistically test for significant changes in mean trait values within the community, thereby giving an indication of how the functional composition of the assemblage has changed through time. Because many traits showed non‐linear trends, significance of trait changes was tested by grouping data by decade and performing analysis of variance (ANOVA) to test for significance differences in mean trait value among decades. To avoid excessive testing and inflated experiment‐wise error rate, only selected traits were tested for temporal trends. In particular, traits that were related to life‐history strategy, trophic relationships and environmental relationships were tested in order to identify which life‐history types are contributing to changing functional diversity and identify whether trait data were reflective of increasing presence of warm‐water‐associated predators. The traits selected were trophic level, maximum temperature, piscivory, age at maturity, natural mortality, maximum age, L_∞_, generation time, and parental care.

### 2.4 Abundance analyses

Principal component analysis (PCA) was used on CPUE data to identify which species contributed most to changing abundances through time within each bay and season. The “prcomp” function in R was used for calculating the principal components. Species were then ranked by the magnitude of their PC1 and PC2 loadings (i.e., highest magnitude has rank 1, second highest magnitude has rank 2, etc.). Ranks were summed across all bays for each season, with the species having the lowest rank sum contributing most to changing abundances within the bays. Temporal trends in abundance for species identified as important by PCA are shown in the results section. Additionally, species were classified in terms of occurrence (temperate, subtropical, or tropical) and abundance trends for the most commonly occurring tropical species were examined in order to assess whether there was evidence of tropical species expansion. Species occurrence was classified based on the classification listed on FishBase.

## 3 RESULTS

### 3.1 Functional diversity indices

**FIGURE 2:**
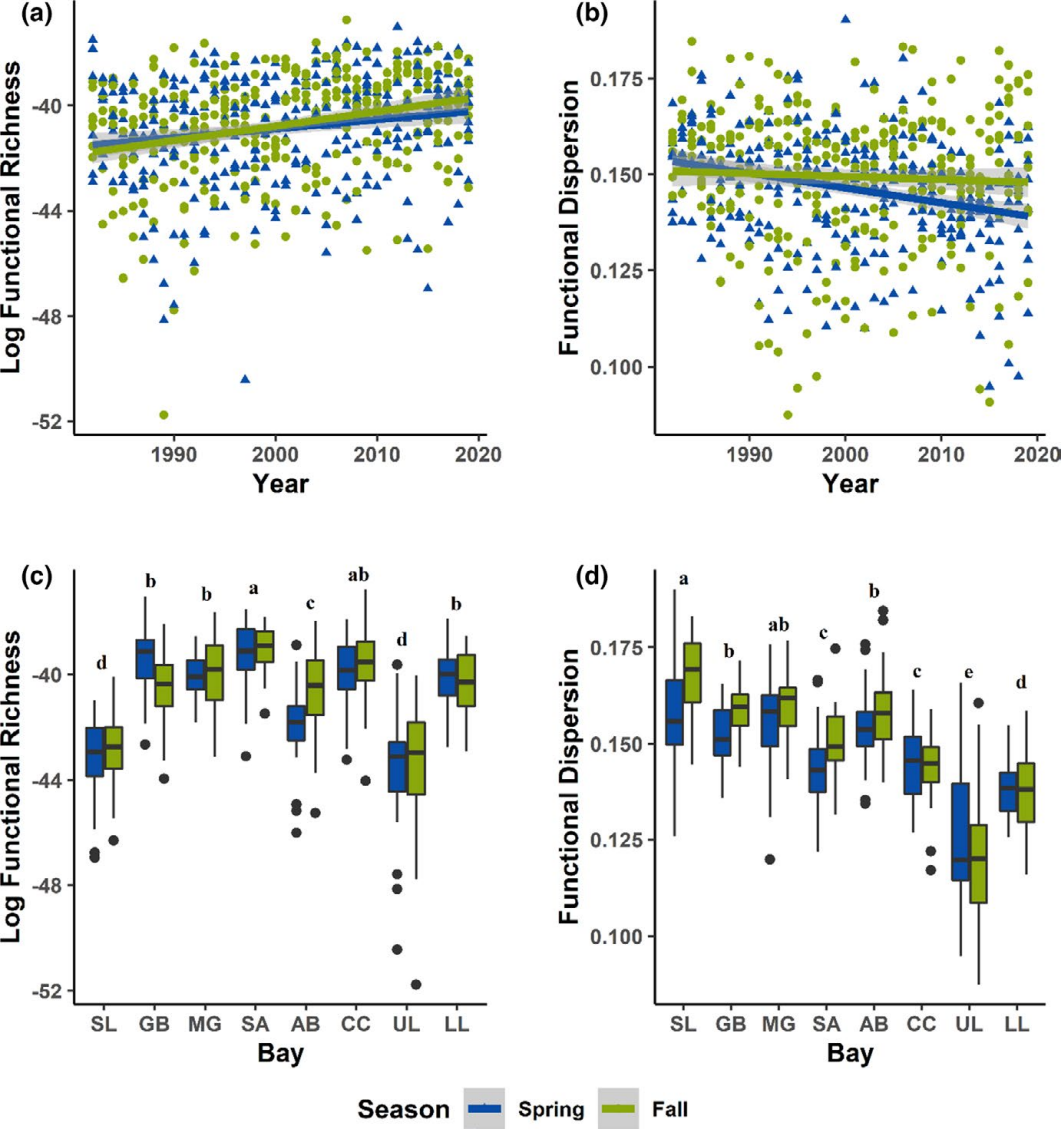
Spatial and temporal trends in functional diversity. Temporal trends in (a) log‐functional richness, and (b) functional dispersion are shown for spring and fall, with spring shown with blue triangles, and fall shown with green dots. Spatial differences in (c) log‐functional richness, and (d) functional dispersion are shown for spring and fall, with spring shown in blue, and fall shown in green, with the bays arranged from north to south along the x‐axis. Letters above boxplots represent groups of bays that are not significantly different. Bay abbreviations are as follows, SL: Sabine Lake, GB: Galveston Bay, MG: Matagorda Bay, SA: San Antonio Bay, AB: Aransas Bay, CC: Corpus Christi Bay, UL: Upper Laguna Madre, LL: Lower Laguna Madre

For both spring and fall, functional diversity indices were found to vary across space and through time. Functional richness (FRic) showed an increasing trend across all bays in both spring and fall (logFRic ~ Year, *p* = .0018 and *p* < .0001 for spring and fall, respectively, Figure 2a). Functional dispersion (FDis) decreased significantly through time in spring (*p* < .0001), but not fall (*p* = .404). Although fall did not show a significant linear trend, there appeared to be non‐linear trend in fall, with FDis initially decreasing, and subsequently increasing (Figure 2b). Natural log of functional richness was significantly different among bays (*p* < .0001), but not among seasons (*p* = .324). Although significant differences in functional richness were evident among bays, no clear spatial pattern was evident (Figure 2c, Table 2). For functional dispersion, both among bay differences (*p* < .0001) and between season differences (*p* = .0005) were highly significant. There was a clear pattern of higher FDis in the north compared to the south (Figure 2d, Table 3), meaning that bays in the south were more strongly dominated by fewer trait types, while in the north assemblages were somewhat less clumped in trait space (although overall, FDis was relatively low for all bays). Functional dispersion was generally higher in fall than in spring, suggesting that for a given bay, the fall assemblage was less clumped in trait space.

**TABLE 2:** Tukeyʼs HSD for FRic. Tukeyʼs honestly significant difference among bays for (a) spring and (b) fall functional richness

FRic	GB	MG	SA	AB	CC	UL	LL
(a) Spring							
SL	**<0.0001**	0.2160	**<0.0001**	0.9992	**0.0017**	1	0.0920
GB		**0.0007**	0.9907	**<0.0001**	0.1450	**<0.0001**	**0.0030**
MG			**<0.0001**	0.5184	0.7312	0.2376	0.9999
SA				**<0.0001**	**0.0132**	**<0.0001**	**0.0001**
AB					**0.0095**	0.9998	0.2832
CC						**0.0017**	0.9146
UL							0.1008
(b) Fall							
SL	0.5681	**0.0015**	**<0.0001**	0.398	**<0.0001**	1	0.3856
GB		0.3195	**0.0001**	1	**0.0016**	0.5222	1
MG			0.1967	0.4842	0.6241	**0.0009**	0.4979
SA				**0.0003**	0.9966	**<0.0001**	**0.0003**
AB					**0.0044**	0.3522	1
CC						**<0.0001**	**0.0048**
UL							0.3402

Note

Table shows the adjusted *p*‐value for a given comparison (e.g., difference in FRic between Sabine Lake and Galveston Bay). Cells containing *p*‐values significant at the 0.05 significance level are in bold. Bay abbreviations are as follows, SL: Sabine Lake, GB: Galveston Bay, MG: Matagorda Bay, SA: San Antonio Bay, AB: Aransas Bay, CC: Corpus Christi Bay, UL: Upper Laguna Madre, LL: Lower Laguna Madre.

**TABLE 3:** Tukeyʼs HSD for FDis. Tukeyʼs honestly significant difference among bays for (a) spring and (b) fall functional dispersion

FDis	GB	MG	SA	AB	CC	UL	LL
(a) Spring							
SL	0.6385	0.9937	**<0.0001**	0.9678	**0.0004**	**<0.0001**	**<0.0001**
GB		0.9720	**0.0259**	0.9951	0.1272	**<0.0001**	**<0.0001**
MG			**0.0006**	1	**0.0059**	**<0.0001**	**<0.0001**
SA				**0.0018**	0.9991	**<0.0001**	0.5148
AB					**0.0145**	**<0.0001**	**<0.0001**
CC						**<0.0001**	0.1875
UL							**0.0005**
(b) Fall							
SL	**0.0032**	**0.0142**	**<0.0001**	**0.0003**	**<0.0001**	**<0.0001**	**<0.0001**
GB		0.9999	**0.0060**	0.9989	**<0.0001**	**<0.0001**	**<0.0001**
MG			**0.0012**	0.9698	**<0.0001**	**<0.0001**	**<0.0001**
SA				**0.0421**	0.1320	**<0.0001**	**<0.0001**
AB					**<0.0001**	**<0.0001**	**<0.0001**
CC						**<0.0001**	0.1050
UL							**<0.0001**

Note

Table shows the adjusted *p*‐value for a given comparison (e.g., difference in FDis between Sabine Lake and Galveston Bay). Cells containing *p*‐values significant at the 0.05 significance level are in bold. Bay abbreviations are as follows, SL: Sabine Lake, GB: Galveston Bay, MG: Matagorda Bay, SA: San Antonio Bay, AB: Aransas Bay, CC: Corpus Christi Bay, UL: Upper Laguna Madre, LL: Lower Laguna Madre.

### 3.2 Analysis of trait means

**FIGURE 3:**
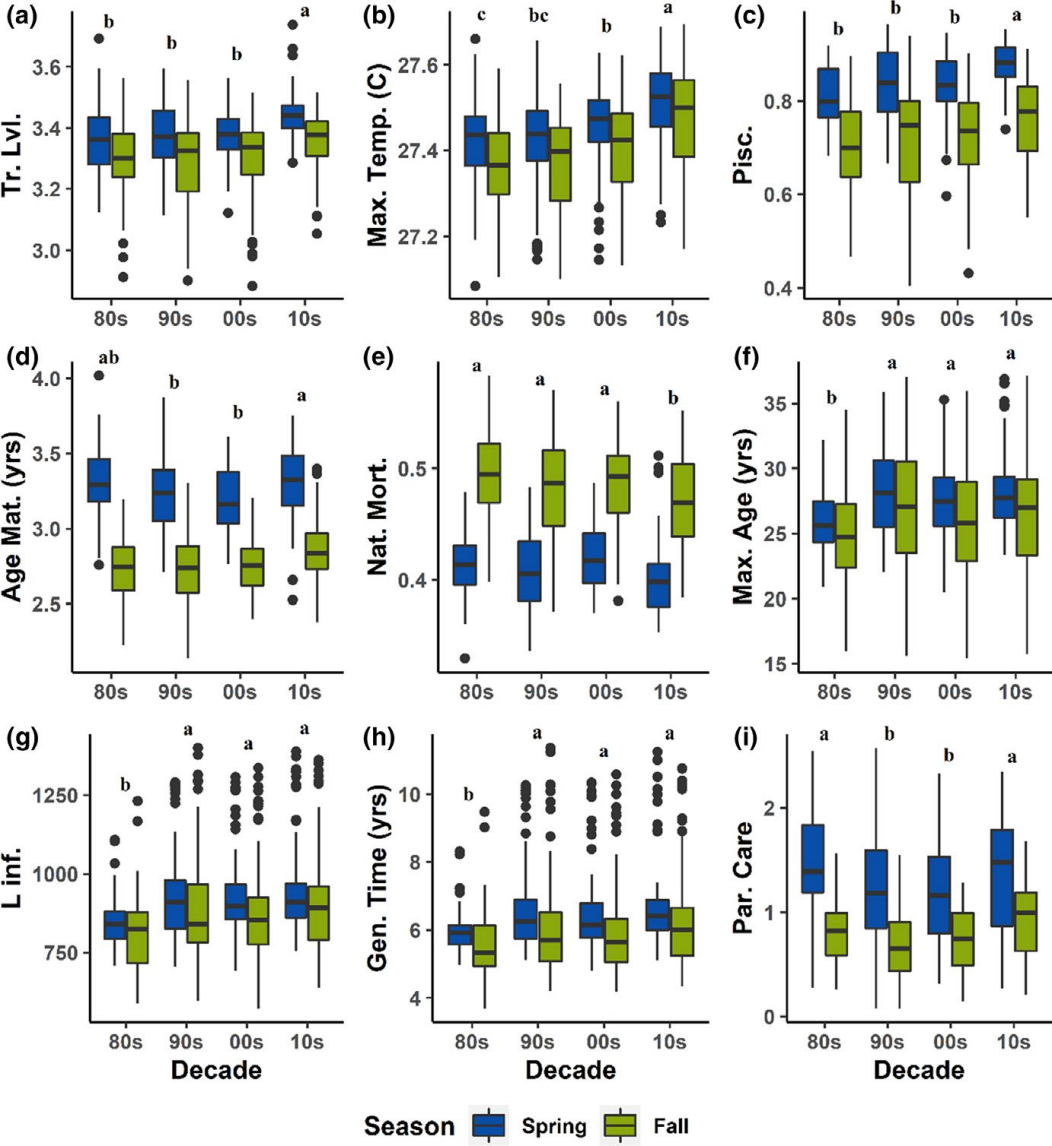
Community‐weighted mean trait values by decade. CWM trait values are shown with data grouped by decade for (a) Trophic level, (b) Maximum temperature observed (°C), (c) Piscivory, (d) Age at maturity (years), (e) Natural mortality rate, (f) Maximum age (years), (g) L_∞_ (mm), (h) Generation time (years), and (i) Parental care. Spring data are shown in blue, and fall data are shown in green. Letters above boxplots represent groups of decades that are not significantly different

Community‐weighted mean trait values were calculated for all functional traits in each bay and year for each season separately, tables containing all community‐weight trait means are presented in Appendix: Tables A1, A2 and A1, A2. Nine functional traits were selected for testing of significant temporal and seasonal trends: trophic level, maximum temperature, piscivory, age at maturity, natural mortality, maximum age, L infinity, generation time, and parental care. All nine traits had significant decade and season terms at an alpha of 0.05, with *p*‐values ranging from a maximum of .0002 to a minimum of 2.56 × 10^−104^ (effectively 0). Tukeyʼs honestly significant difference (Tukeyʼs HSD) for traits by decade and season are shown in Tables 4 and 5, respectively. ANOVA tables are shown in Appendix: Tables A3, A4, A5, A6, A7, A8, A9, A10, A11. Mean trophic level, maximum temperature, piscivory, maximum age, L infinity, and generation time all increased through time and were higher in spring than in fall (Figure 3a–c,f–h). Natural mortality decreased through time and was significantly lower in spring than in fall (Figure 3e), which was expected, given that larger, longer lived individuals tend to have lower natural mortality rates. Age at maturity and parental care showed a “U”‐shaped trend (Figure 3d,i), with initial decreases and subsequent increases, likely indicative of increased equilibrium strategists (i.e., species with well‐developed parental care and delayed maturity) in more recent years.

**TABLE 4:** Tukeyʼs HSD for traits – Decade. Tukeyʼs honestly significant difference among decades for (a) Trophic level, (b) Maximum temperature, (c) Piscivore, (d) Age at maturity, (e) Natural mortality, (f) Maximum age, (g) Asymptotic length (L_∞_), (h) Generation time, and (i) Parental care

Tukeyʼs HSD for traits – decade							
(a) Trophic level				(b) Maximum temperature			
	1990s	2000s	2010s		1990s	2000s	2010s
1980s	0.9257	0.4616	**<0.0001**	1980s	0.9775	**0.0321**	**<0.0001**
1990s		0.8002	**<0.0001**	1990s		0.0578	**<0.0001**
2000s			**0.0001**	2000s			**<0.0001**

(c) Piscivore				(d) Age at maturity			
	1990s	2000s	2010s		1990s	2000s	2010s
1980s	0.1659	0.1647	**<0.0001**	1980s	0.557	0.2094	0.1884
1990s		1	**0.0001**	1990s		0.9013	**0.0021**
2000s			**0.0002**	2000s			**0.0001**

(e) Natural mortality				(f) Maximum age			
	1990s	2000s	2010s		1990s	2000s	2010s
1980s	0.1256	0.7883	**<0.0001**	1980s	**<0.0001**	**0.0041**	**<0.0001**
1990s		0.5213	**0.0452**	1990s		0.4290	0.9991
2000s			**0.0004**	2000s			0.5142

(g) Asymptotic length				(h) Generation time			
	1990s	2000s	2010s		1990s	2000s	2010s
1980s	**<0.0001**	**0.0002**	**<0.0001**	1980s	**0.0003**	**0.0039**	**<0.0001**
1990s		0.9645	0.8788	1990s		0.8734	0.9217
2000s			0.6141	2000s			0.5064

(i) Parental care							
	1990s	2000s	2010s				
1980s	**0.006**	**0.0082**	0.9965				
1990s		0.9997	**0.0054**				
2000s			**0.0075**				

Note

Table shows the adjusted *p*‐value for a given comparison (e.g., difference between 1980s and 1990s). Cells containing p‐values significant at the .05 significance level are in bold.

**TABLE 5:** Tukeyʼs HSD for traits – season. Tukeyʼs honestly significant difference among spring and fall for (a) Trophic level, (b) Maximum temperature, (c) Piscivore, (d) Age at maturity, (e) Natural mortality, (f) Maximum age, (g) Asymptotic length (L_∞_), (h) Generation time, and (i) Parental care. Table shows the adjusted *p*‐value for a given comparison (e.g., difference between 1980s and 1990s)

Tukeyʼs HSD for traits – season	
Trait	*p*‐value
Trophic level	<.0001
Maximum temperature	<.0001
Piscivore	<.0001
Age at maturity	<.0001
Natural mortality	<.0001
Maximum age	<.0001
Asymptotic length	.0002
Generation time	<.0001
Parental care	<.0001

### 3.3 Abundance analyses

**FIGURE 4:**
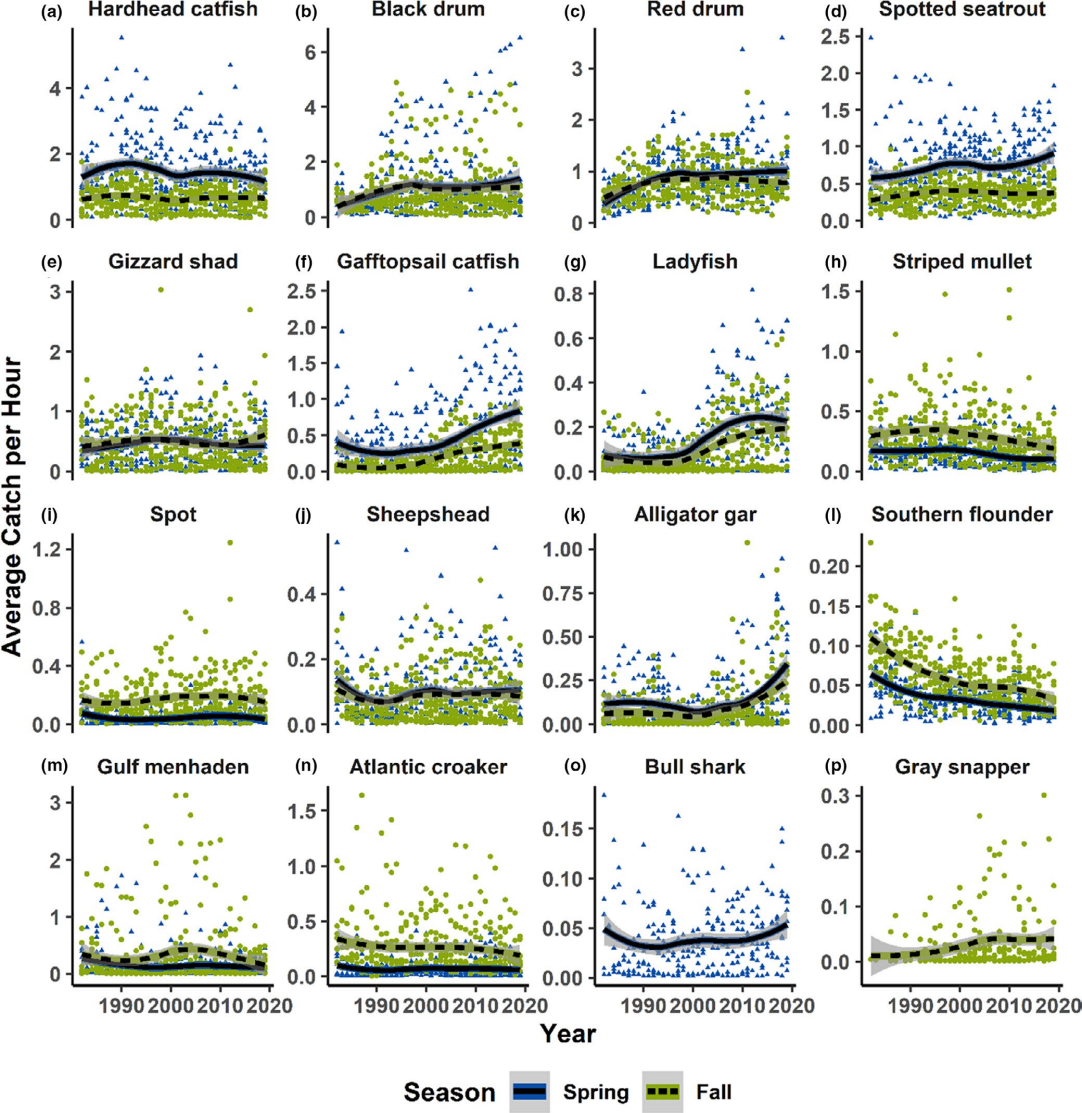
Abundance of top‐ranked species. Average catch per hour data and loess trend is shown for the top 15 ranked species, in spring (blue triangles, solid black line) and fall (green dots, dashed black line), for (a) Hardhead catfish (*Ariopsis felis*), (b) Black drum (*Pogonias cromis*), (c) Red drum (*Sciaenops ocellatus*), (d) Spotted seatrout (*Cynoscion nebulosus*), (e) Gizzard shad (*Dorosoma cepedianum*), (f) Gafftopsail catfish (*Bagre marinus*), (g) Ladyfish (*Elops saurus*), (h) Striped mullet (*Mugil cephalus*), (i) Spot (*Leiostomus xanthurus*), (j) Sheepshead (*Archosargus probatocephalus*), (k) Alligator gar (*Atractosteus spatula*), (l) Southern flounder (*Paralichthys lethostigma*), (m) Gulf menhaden (*Brevoortia patronus*), (n) Atlantic croaker (*Micropogonias undulatus*), (o) Bull shark (*Carcharhinus leucas*), and (p) Gray snapper (*Lutjanus griseus*) (note: there are 16 plots as Bull shark was in the top 15 in spring but not fall, and Gray snapper was in the top 15 in fall but not spring)

**FIGURE 5:**
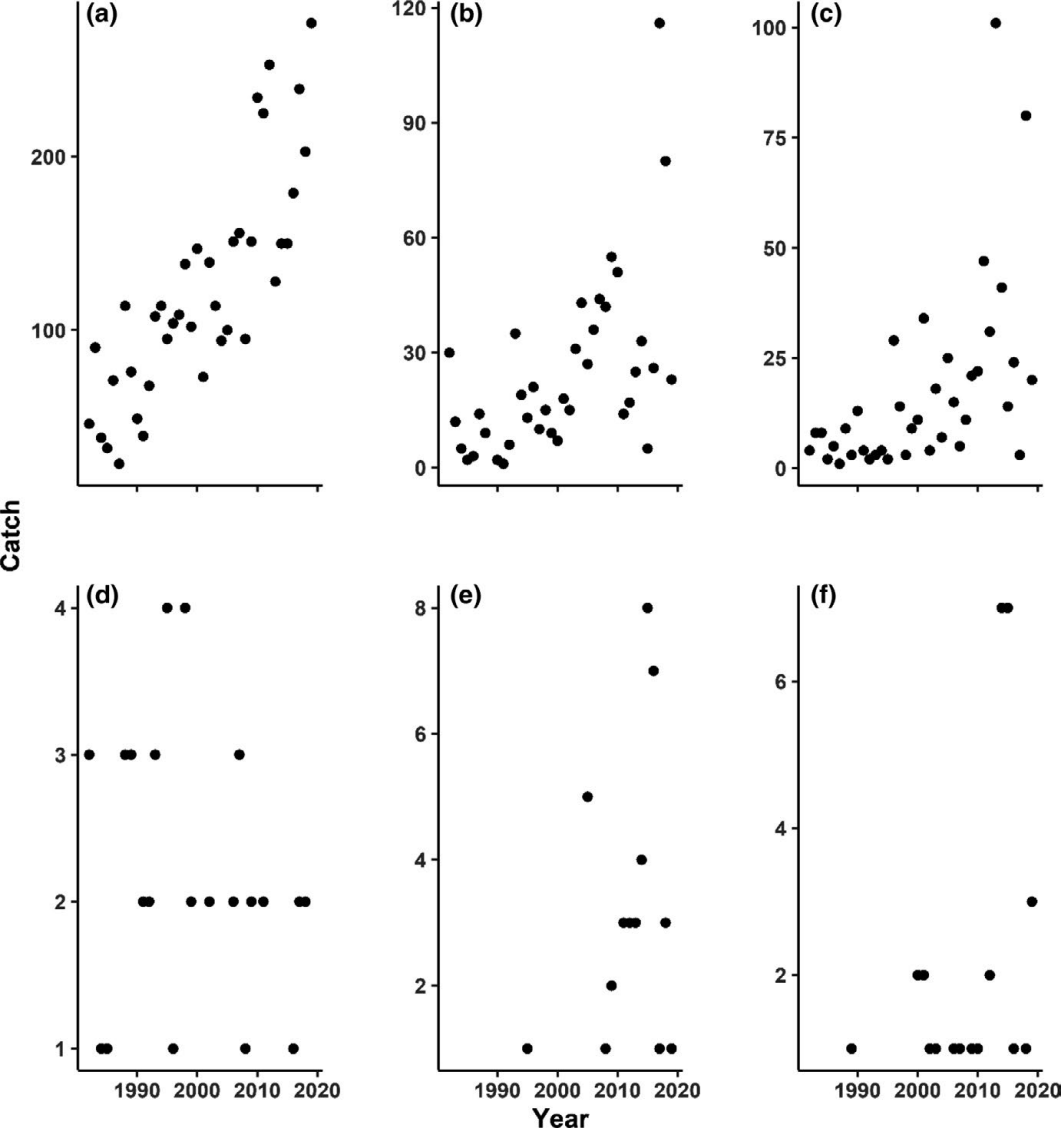
Catch rates of commonly encountered tropical species. Total catch across all bays for a given year is shown for (a) Cownose ray (*Rhinoptera bonasus*), (b) Common snook (*Centropomus undecimalis*), (c) Scalloped hammerhead (*Sphyrna lewini*), (d) Inshore lizardfish (*Synodus foetens*), (e) Gulf pipefish (*Syngnathus scovelli*), and (f) Irish pompano (*Diapterus auratus*)

Principal component analysis was run on the species abundance data for each season and bay, to identify which species were contributing most to temporal trends in functional diversity and trait means. Tables showing the total number of individuals caught throughout the study period for each species, and the proportion of total catch by species are presented in Appendix: Tables A12, A13. Species were ranked using PCA to identify the most important species contributing to changing assemblage structure in each season. The abundances for the 15 most important species in each season are shown in Figure 4. In addition to the species identified as important by PCA, the most commonly encountered tropical species are identified, and catch rates are shown in Figure 5. The six most commonly encountered tropical species are, respectively, Cownose ray (*Rhinoptera bonasus*), Common snook (*Centropomus undecimalis*), Scalloped hammerhead (*Sphyrna lewini*), Inshore lizardfish (*Synodus foetens*), Gulf pipefish (*Syngnathus scovelli*), and Irish pompano (*Diapterus auratus*). All but Inshore lizardfish show increasing catch rates through time.

## 4 DISCUSSION

In this study, we found that increasing species diversity (Pawluk et al., 2021) is associated with increasing functional richness, but stable or decreasing functional dispersion (Figure 2). The trait analysis indicated that these trends were predominantly driven by increasing prevalence of large, long‐lived, later‐maturing, and warm‐water‐associated species (Figure 3). Abundance analyses confirmed that these trends were driven both by greater abundance of resident species with these functional traits, as well as increasing prevalence of tropical species into the bays in the northwestern Gulf of Mexico (Figures 3 and 4). Although species characterized as subtropical make up the vast majority of the assemblage, the proportion of total catch made up of tropical species increased 1.8‐fold when comparing the period of 1982–2000 to 2001–2019 (Appendix: Table A14), and the individual trends for the most widely encountered tropical species showed increasing occurrence through time.

Along the Texas coast, as in many marine ecosystems (Collie et al., 2008; Hiddink & Ter Hofstede, 2008; Perry et al., 2005), climate change has led to shifts in the spatial distributions and abundances of invertebrates and fishes that yield an overall increase in species diversity (Fujiwara et al., 2019; Pawluk et al., 2021). While biodiversity is often thought to confer benefits on ecosystem functioning through a positive correlation with functional diversity (Hooper et al., 2005), the relationship between species and functional diversity often differs across ecosystems and functions considered (Cadotte et al., 2011). Therefore, an increase in species diversity does not guarantee increased ecosystem services and functioning. The observed increase in FRic found in this study implies new values along one or more trait axes within the fish assemblages of the Texas bays (Villéger et al., 2008). The observed increase in functional richness with a concurrent decrease in functional dispersion suggests that, while new functional types may have entered the bays, the majority of the species entering the bays are functionally redundant. Therefore, the functional benefit of the increasing biodiversity resultant from tropical species expansion may be somewhat limited. However, functional redundancy has been shown to increase ecosystem stability and resilience (Biggs et al., 2020), and therefore, increasing species diversity, coupled with stable or decreasing FDis, may indicate stability and resilience for those functions provided by the most abundant functional types.

The functional types increasing in prevalence, as determined by the trait analyses, were those whose trait values were associated with being larger, slower maturing, longer lived, and associated with warmer temperatures (Figure 3). These trait types correspond to periodic (e.g., black drum (*Pogonias cromis*), red drum (*Sciaenops ocellatus*), ladyfish (*Elops saurus*), and gray snapper (*Lutjanus griseus*)) and equilibrium (e.g., bull shark (*Carcharhinus leucas*), cownose ray, and bonnethead shark (*Sphyrna tiburo*)) strategists on the trilateral life‐history continuum (Winemiller & Rose, 1992), or K‐selected on the r‐K continuum (Pianka, 1970). Both periodic and equilibrium strategists are relatively large and long‐lived, with the main distinction being between a higher investment in fecundity for periodic strategists, and in parental care for equilibrium strategists, producing fewer highly developed offspring. The trait analysis found an initial decrease in the community‐weighted mean for parental care, followed by an increase (Figure 3i), which suggests that initially, periodic strategists were increasing in relative abundance, followed by a period of increasing relative abundance of equilibrium strategists as well. An increase in k‐selected species is consistent with previous findings that most Atlantic species fall more toward the periodic endpoint of the trilateral continuum (Vila‐Gispert et al., 2002), as well as with a previous finding showing a shift in dominance from r‐selected species to K‐selected species in an estuary within the English channel response to climate warming coupled with the warm phase of the Atlantic Multi‐decadal Oscillation (AMO) (McLean et al., 2019).

The observed increase in abundance of periodic and equilibrium strategists (e.g., red drum, ladyfish, bull shark) likely results from a bottom‐up trophic cascade driven by two main mechanisms: (1) relaxed abiotic filter (increasing temperature) removing physiological barrier to expansion, and (2) relaxed biotic filter (increased abundance and diversity of invertebrates) minimizing competition with resident species, which would allow for successful establishment by expanding species. A previous study found invertebrates to be highly important forage species for abundant predators in the Gulf of Mexico (Fujiwara et al., 2016). In this study, approximately 97% of the species included in this study, for which diet data were available, incorporate invertebrates into their diet to some extent. The increasing invertebrate diversity and abundance within the bays (Fujiwara et al., 2019), coupled with increasing temperatures may have allowed for the expansion of both tropical and subtropical invertivores which could have led to greater abundance of piscivores within the bays.

The principal component analysis (PCA) identified which species were contributing most to changing abundance, and thus changing trait means (Figure 4). The species contributing most to changing abundance consisted mainly of several common sciaenid species (e.g., red drum, black drum, Atlantic croaker (*Micropogonias undulatus*), and spotted seatrout (*Cynoscion nebulosus*)) as well as several expanding tropical species (e.g., bull shark, gray snapper, and gafftopsail catfish (*Bagre marinus*)). Most species appear to show either stable or increasing trends, although Southern flounder (*Paralichthys lethostigma*) shows a strong decreasing trend. The fact that most species show stable or increasing trends is indicative that the changing trait means are due to increasing abundance of those trait types, as opposed to decreasing abundance of opposite trait types (e.g., decreasing abundance of small fish would increase the *relative* abundance of large fish without actually changing the abundance of large fish). The consistent decline in Southern flounder is probably due, at least in part, to rising temperatures. Southern flounder is known to exhibit temperature‐dependent sex determination, whereby warmer temperatures can lead to masculinization of the population (Honeycutt et al., 2019), and temperature is known to affect recruitment in some estuaries (Erickson et al., 2021). However, temperature may not be solely responsible, both commercial and recreational fisheries significantly impact southern flounder populations through bycatch and target fishing, respectively (Matlock, 1982). In general, the trait means for the most important species did not significantly differ from the overall trait means, suggesting that these species are likely contributing most to changing abundance due to their high relative abundance (i.e., most abundant species contribute most to changing abundance).

Our results indicate that climate change is important in driving fish assemblage shifts in the northwestern Gulf of Mexico because of the ways tropical and subtropical species are shifting their distributions. However, many other anthropogenic factors may also be contributing to ecosystem change at the same time. For example, recreational and commercial fishing can significantly impact fish population dynamics (Radford et al., 2018), and the extent of their impact is dependent on the life‐history strategies of the fish (Fujiwara, 2012). Thus, fishing pressure may impact local fish community composition and diversity (Farriols et al., 2017; Pérez‐Matus et al., 2017). In Texas, total recreational fishing landings for sport boat anglers decreased through time from 1974 to 2008 (Green & Campbell, 2010). A decrease in recreational fishing mortality for sport fish may have contributed to increasing abundance of large, long‐lived species, which were shown to have increased in this study. Species such as red drum, spotted seatrout, sheepshead (*Archosargus probatocephalus*), and black drum are commonly targeted by recreational fishers and were shown to be increasing in abundance in our study.

In addition to recreational fishing pressure, Texas coastal fish are impacted by commercial fishing pressure by the Gulf of Mexico shrimp fishery in the form of bycatch mortality. While total commercial fisheries landings (by weight) in Texas remained relatively stable for the period of 1981–2001, total finfish landings have increased through time, with species such as black drum, flounder species, and sheepshead being frequently caught as bycatch (Culbertson et al., 2004). Increasing fisheries pressure is generally thought to negatively impact biodiversity (Hall et al., 2006); however, the impact of bycatch mortality on a few widely abundant species may have allowed for proliferation of otherwise excluded species.

In addition to changes in fishing pressure, anthropogenic impacts to habitat may have contributed to changing community composition. For example, the number of oil platforms in the Gulf of Mexico increased dramatically through time peaking at approximately 4,000 active platforms in the Gulf of Mexico in 2007 (Priest, 2007). Although active platforms have decreased to 1,862 as of April, 2019 according to the Bureau of Safety and Environmental Enforcement, many decommissioned platforms have been converted into artificial reef habitat (BSEE, 2021). Both active and decommissioned oil platforms serve as important habitat for both reef‐associated and pelagic species (Reynolds, 2015; Stanley & Wilson, 1997), and may serve as stepping stones, increasing species dispersal capacity (Sammarco et al., 2004). Tropical species may, therefore, have gained increased access to the bays of Texas through time as a result of the increasing presence of oil platforms; however, characterization of the communities present on oil platforms near Texas bays showed little overlap among the species observed at platforms, and those encountered in this study (Rooker et al., 1997). It is, therefore, unlikely that oil platforms are the main factor driving increasing species diversity in Texas bays, although it may be one contributing factor.

Another anthropogenic impact that may have contributed to changing coastal ecosystems is increased nutrient input through agricultural runoff. Increased fertilization from agriculture along the Mississippi River has led to increasing nitrogen loading in the Gulf of Mexico (Goolsby et al., 1999; Tian et al., 2020). If increased nutrient input led to increased primary productivity, it could feasibly lead to increased fish production, and possibly increasing diversity. An increase in abundance of prey fish species could potentially decrease competitive pressures, allowing for expansion of previously excluded species; however, data from the Gulf of Mexico show no evidence of long‐term change in net primary production (Muller‐Karger et al., 2015).

Overall, this study found that increasing species diversity of Texas Gulf coast fishes has been accompanied by a reduction in functional diversity, with long‐lived, large, predatory species increasing in prevalence. Changes in community structure, including greater prevalence of tropical species, may have altered the intensity of species interactions, such as competition and predation, with negative effects on certain native species. Evidence from this study further suggests that climate change is one of the important factors contributing to the changing fish communities, although the observed changes are likely the result of many factors acting in concert. The results observed in this study are likely not unique to Texas, as many coastal ecosystems are currently experiencing shifts in the geographic distributions of marine species (Perry et al., 2005; Wernberg et al., 2016). Future research is needed to identify whether the pattern of functional homogenization and/or decreasing functional diversity following species expansion is consistent across other subtropical and temperate coastal ecosystems.

## CONFLICT OF INTEREST

The authors declare that they have no conflict of interest.

## AUTHOR CONTRIBUTIONS

**Michaela Pawluk:** Conceptualization (lead); Formal analysis (lead); Visualization (lead); Writing – original draft (lead); Writing – review & editing (equal). **Masami Fujiwara:** Conceptualization (supporting); Funding acquisition (lead); Supervision (lead); Writing – review & editing (equal). **Fernando Martinez‐Andrade:** Conceptualization (supporting); Data curation (lead); Writing – review & editing (equal).

## Data Availability

Data are freely available at: https://www.bco‐dmo.org/dataset/828794. https://doi.org/10.26008/1912/bco‐dmo.828794.1.

## 1

**TABLE A1:** Community‐weighted means for functional traits, part 1. Community‐weighted trait means for maximum length in mm (Lmax), common length in mm (Lcom), length at maturity (Lmat), age at maturity (Amat), maximum age (Tmax), maximum weight (Wmax), asymptotic length (Linf), von Bertalanffy growth parameter (K), hypothetical age at length 0 (t0), natural mortality rate (M), generation time (GenT), trophic level (TrLvl), and mouth position (MoPos). Season abbreviations are SP: spring, FA: fall. Bay abbreviations are as follows, SL: Sabine Lake, GB: Galveston Bay, MG: Matagorda Bay, SA: San Antonio Bay, AB: Aransas Bay, CC: Corpus Christi Bay, UL: Upper Laguna Madre, LL: Lower Laguna Madre

Season	Bay	Year	Lmax	Lcom	Lmat	Amat	Tmax	Wmax	Linf	K	t0	M	GenT	TrLvl	MoPos
SP	SL	1986	1328.73	789.87	633.06	3.43	24.42	22.38	1032.59	0.23	−0.62	0.36	6.15	3.60	2.93
		1987	1530.92	929.91	709.44	3.32	27.25	34.87	996.86	0.24	−0.59	0.38	6.44	3.69	2.94
		1988	1269.39	749.48	577.93	3.00	25.00	25.43	887.86	0.28	−0.55	0.44	5.49	3.47	2.87
		1989	1320.08	763.83	586.23	2.76	27.54	30.19	891.92	0.30	−0.51	0.47	5.65	3.41	2.77
		1990	1420.26	856.37	625.72	2.95	28.53	30.99	974.24	0.28	−0.52	0.44	5.64	3.55	2.78
		1991	1477.79	871.25	667.32	3.12	28.14	32.65	1041.62	0.26	−0.55	0.40	6.29	3.59	2.77
		1992	1320.60	754.58	552.77	2.78	30.60	34.10	843.91	0.31	−0.53	0.48	5.35	3.43	2.70
		1993	1420.94	783.11	617.77	3.08	29.54	36.63	923.14	0.27	−0.58	0.44	6.39	3.56	2.82
		1994	1242.82	654.42	550.19	2.95	26.60	28.24	885.05	0.28	−0.57	0.45	6.08	3.35	2.83
		1995	1364.51	801.23	582.14	2.80	28.36	31.82	913.80	0.30	−0.51	0.47	5.43	3.47	2.75
		1996	1393.59	830.48	605.97	2.71	28.31	28.52	1008.58	0.30	−0.47	0.46	5.49	3.50	2.71
		1997	1333.05	746.03	585.09	2.85	27.82	29.31	970.76	0.29	−0.52	0.46	5.99	3.47	2.75
		1998	1257.90	698.67	553.54	3.02	26.20	22.11	1016.87	0.27	−0.56	0.43	5.75	3.50	2.86
		1999	1176.58	663.56	531.23	3.05	23.42	20.60	949.95	0.27	−0.58	0.44	5.66	3.44	2.89
		2000	1085.00	608.65	492.80	2.77	20.89	17.71	902.52	0.29	−0.54	0.48	5.38	3.20	2.92
		2001	1212.43	653.05	538.40	3.04	26.50	24.33	958.63	0.28	−0.59	0.44	6.12	3.48	2.85
		2002	1146.34	588.06	501.99	2.89	27.12	25.09	893.13	0.28	−0.58	0.46	5.95	3.31	2.79
		2003	1210.99	669.82	547.85	3.07	25.84	26.74	945.77	0.28	−0.59	0.44	6.15	3.39	2.80
		2004	1243.00	668.75	535.30	2.99	27.89	30.83	980.52	0.29	−0.58	0.46	6.29	3.40	2.73
		2005	1308.88	682.44	589.40	2.86	28.27	28.79	1008.91	0.28	−0.53	0.45	6.71	3.42	2.74
		2006	1116.89	597.99	510.24	2.92	23.86	22.47	860.94	0.29	−0.58	0.46	5.85	3.29	2.87
		2007	1237.87	672.88	525.61	2.88	30.78	28.83	909.47	0.29	−0.57	0.46	5.82	3.44	2.73
		2008	1171.39	620.27	515.22	2.80	27.68	27.32	869.25	0.30	−0.56	0.48	5.97	3.31	2.76
		2009	1358.07	760.15	582.18	2.79	30.99	35.45	939.00	0.29	−0.53	0.47	6.16	3.43	2.67
		2010	1418.55	816.41	557.44	2.53	36.53	39.48	908.99	0.33	−0.48	0.51	5.31	3.54	2.52
		2011	1262.29	723.85	527.69	2.66	30.16	31.18	854.79	0.32	−0.51	0.50	5.31	3.43	2.67
		2012	1330.62	776.19	550.40	2.66	31.73	34.53	861.34	0.32	−0.51	0.50	5.26	3.46	2.67
		2013	1257.95	681.03	542.95	3.03	29.29	29.99	904.98	0.28	−0.59	0.45	6.04	3.53	2.76
		2014	1427.72	837.64	604.49	2.90	32.62	36.77	930.30	0.30	−0.53	0.46	5.73	3.57	2.68
		2015	1408.99	811.31	610.23	3.05	30.33	34.24	946.17	0.28	−0.57	0.44	6.00	3.64	2.78
		2016	1469.56	852.05	665.51	3.42	29.39	33.11	1043.36	0.24	−0.63	0.38	6.67	3.74	2.85
		2017	1423.52	796.38	658.19	3.22	28.48	34.44	991.62	0.25	−0.60	0.40	6.97	3.57	2.81
		2018	1462.22	815.03	644.53	3.10	32.14	39.02	990.36	0.27	−0.58	0.42	6.82	3.55	2.71
		2019	1547.39	879.34	693.88	3.19	32.62	39.42	1063.41	0.25	−0.57	0.39	7.12	3.66	2.71
SP	GB	1982	895.55	421.11	416.91	3.29	24.39	14.36	757.78	0.25	−0.69	0.41	5.40	3.21	2.91
		1983	890.51	402.12	415.37	3.27	24.02	14.21	769.82	0.24	−0.69	0.41	5.62	3.19	2.91
		1984	967.36	456.75	429.52	3.11	25.80	18.35	791.43	0.26	−0.65	0.43	5.49	3.23	2.86
		1985	876.85	405.58	410.97	3.36	23.20	14.03	732.72	0.23	−0.72	0.41	5.50	3.17	3.01
		1986	875.99	430.82	389.37	3.06	23.62	14.24	708.46	0.26	−0.66	0.45	4.96	3.16	2.95
		1987	808.40	359.48	387.42	3.49	22.17	10.82	718.42	0.22	−0.75	0.39	5.46	3.15	3.06
		1988	897.96	397.52	425.70	3.61	25.24	14.36	785.87	0.22	−0.75	0.37	5.77	3.28	2.95
		1989	830.52	369.54	396.25	3.47	22.57	11.98	732.88	0.21	−0.76	0.38	5.57	3.12	3.08
		1990	792.12	356.93	388.29	3.59	22.04	10.51	713.62	0.20	−0.78	0.37	5.59	3.11	3.10
		1991	905.64	396.48	443.15	3.65	23.76	15.72	770.71	0.21	−0.76	0.37	6.08	3.23	2.98
		1992	976.36	467.61	439.57	3.42	25.79	19.75	781.95	0.23	−0.72	0.40	5.64	3.26	2.95
		1993	873.42	422.02	403.20	3.40	23.72	14.93	705.38	0.22	−0.74	0.40	5.30	3.16	3.04
		1994	914.48	403.24	433.89	3.78	23.73	16.32	775.53	0.20	−0.79	0.36	6.02	3.25	3.03
		1995	910.47	411.28	425.68	3.71	24.22	16.32	768.08	0.22	−0.77	0.37	5.77	3.26	2.97
		1996	959.23	478.00	415.46	3.09	25.54	18.07	767.66	0.27	−0.64	0.45	5.12	3.26	2.84
		1997	1074.55	537.05	453.74	3.23	27.88	22.30	846.91	0.26	−0.66	0.43	5.55	3.41	2.84
		1998	946.73	468.87	412.04	3.31	23.80	17.32	763.11	0.25	−0.70	0.43	5.30	3.29	2.96
		1999	956.30	465.38	420.10	3.24	24.46	16.83	787.71	0.26	−0.67	0.43	5.44	3.29	2.92
		2000	907.46	419.88	420.88	3.35	23.47	14.73	790.76	0.24	−0.70	0.42	5.78	3.31	2.92
		1992	1137.16	605.39	487.06	3.12	28.18	26.04	816.95	0.28	−0.62	0.45	5.37	3.33	2.80
		1993	1048.87	522.17	472.73	3.54	25.77	22.01	814.19	0.24	−0.72	0.39	5.79	3.32	2.90
		1994	1022.13	506.25	459.03	3.34	23.70	21.53	810.20	0.26	−0.68	0.43	5.81	3.23	2.92
		1995	1041.76	503.07	449.56	3.11	24.42	21.73	818.97	0.28	−0.64	0.45	5.75	3.15	2.88
		1996	942.85	454.16	421.78	3.14	25.03	16.71	785.73	0.27	−0.65	0.44	5.31	3.21	2.86
		1997	1083.09	489.66	472.17	3.24	25.18	21.47	877.13	0.26	−0.66	0.42	6.25	3.28	2.91
		1998	1052.38	522.58	447.94	3.32	25.76	23.13	848.02	0.27	−0.67	0.43	5.69	3.31	2.86
		1999	933.28	447.60	429.39	3.32	22.84	17.28	812.42	0.26	−0.68	0.43	5.76	3.22	2.94
		2000	1055.04	509.76	470.27	3.44	23.93	22.96	901.99	0.25	−0.69	0.42	6.20	3.34	2.86
		2001	959.89	453.53	443.87	3.44	24.51	17.66	838.16	0.24	−0.70	0.40	6.06	3.26	2.91
		2002	1000.71	465.05	463.92	3.55	25.74	20.70	880.03	0.23	−0.72	0.39	6.42	3.30	2.85
		2003	1088.98	530.34	484.08	3.43	27.32	24.03	919.02	0.24	−0.69	0.40	6.42	3.39	2.84
		2004	1118.00	545.10	483.11	3.35	26.48	23.57	898.94	0.25	−0.67	0.41	6.03	3.38	2.87
		2005	1048.94	519.95	469.49	3.46	25.27	21.15	877.72	0.23	−0.71	0.39	6.08	3.33	2.91
		2006	1024.85	527.43	465.10	3.46	23.09	21.60	856.25	0.25	−0.70	0.42	5.98	3.32	2.95
		2007	1083.75	557.01	479.73	3.36	25.21	24.23	848.38	0.26	−0.68	0.42	6.06	3.31	2.91
		2008	1067.80	528.38	488.43	3.28	26.34	22.94	899.03	0.25	−0.66	0.42	6.33	3.38	2.82
		2009	906.14	478.19	425.89	3.26	22.91	16.20	787.85	0.26	−0.68	0.43	5.66	3.23	2.89
		2010	1037.77	520.67	488.52	3.66	24.86	20.05	913.87	0.21	−0.74	0.37	6.49	3.44	2.90
		2011	966.68	519.27	451.86	3.69	23.97	18.07	850.83	0.22	−0.76	0.38	6.06	3.40	2.95
		2012	1042.89	513.22	486.82	3.54	27.15	20.84	904.44	0.22	−0.72	0.37	6.57	3.41	2.83
		2013	1044.31	527.63	484.97	3.51	27.44	21.50	911.51	0.23	−0.71	0.38	6.54	3.42	2.78
		2014	1019.92	530.16	478.32	3.60	25.85	20.00	908.59	0.22	−0.73	0.37	6.31	3.47	2.83
		2015	1172.29	616.50	502.48	3.21	29.41	28.66	889.91	0.26	−0.64	0.43	6.00	3.45	2.76
		2016	979.18	502.46	461.94	3.46	23.41	19.23	839.87	0.24	−0.70	0.41	6.14	3.30	2.94
		2017	1122.29	584.34	517.16	3.34	26.07	25.20	895.48	0.25	−0.67	0.41	6.38	3.40	2.85
		2018	1189.52	638.69	544.39	3.58	27.80	30.78	899.63	0.23	−0.72	0.39	6.70	3.46	2.85
		2019	1193.81	662.42	543.76	3.31	26.95	30.43	841.27	0.26	−0.66	0.42	6.20	3.37	2.86
SP	SA	1982	994.84	480.87	471.54	3.75	25.22	19.68	840.12	0.21	−0.77	0.36	6.56	3.37	2.98
		1983	969.93	495.71	447.87	3.57	25.19	17.29	821.37	0.22	−0.74	0.38	6.01	3.38	2.92
		1984	900.09	466.82	434.89	3.75	22.52	15.30	782.46	0.21	−0.78	0.37	5.80	3.30	3.00
		1985	1020.42	499.23	472.96	4.02	24.40	21.82	803.28	0.18	−0.84	0.33	6.25	3.39	3.06
		1986	1007.58	506.82	442.12	3.62	27.68	19.29	795.19	0.22	−0.75	0.38	5.57	3.43	2.93
		1987	1113.28	558.85	463.92	3.28	28.92	23.70	866.49	0.26	−0.66	0.42	5.56	3.41	2.84
		1988	1262.17	659.54	535.82	3.30	31.87	32.60	852.13	0.26	−0.66	0.41	5.96	3.47	2.79
		1989	1075.80	526.11	495.60	3.67	27.47	23.37	801.76	0.22	−0.75	0.37	6.05	3.38	2.93
		1990	975.04	449.35	462.59	3.87	24.90	18.37	813.13	0.20	−0.79	0.35	6.15	3.35	2.96
		1991	1018.46	446.95	474.46	3.87	25.05	19.20	859.90	0.19	−0.79	0.34	6.40	3.40	2.99
		1992	1251.39	635.31	541.82	3.36	32.67	32.16	926.21	0.25	−0.66	0.40	6.53	3.46	2.70
		1993	1237.70	583.61	552.36	3.37	32.35	30.95	973.13	0.24	−0.67	0.38	7.24	3.42	2.67
		1994	1056.64	515.37	476.27	3.35	28.12	22.06	865.28	0.25	−0.68	0.41	6.24	3.32	2.79
		1995	1016.28	480.07	445.77	3.62	26.17	20.38	829.52	0.24	−0.74	0.39	5.95	3.31	2.89
		1996	1062.19	495.16	455.79	3.25	28.07	20.90	853.40	0.26	−0.66	0.43	5.78	3.37	2.82
		1997	1101.67	483.28	508.40	3.73	28.94	24.69	970.40	0.21	−0.75	0.35	7.36	3.42	2.80
		1998	1064.31	463.50	478.14	3.54	27.51	20.35	913.69	0.22	−0.72	0.38	6.72	3.46	2.90
		1999	1119.44	479.88	476.24	3.49	25.37	24.04	892.79	0.25	−0.70	0.41	6.62	3.28	2.88
		2000	1049.84	465.01	474.69	3.49	25.01	21.34	899.53	0.24	−0.71	0.41	6.66	3.34	2.91
		2001	1227.81	475.43	554.87	3.52	28.99	28.42	1076.72	0.22	−0.70	0.37	8.38	3.37	2.73
		2002	1092.82	459.95	498.66	3.54	28.33	21.94	955.53	0.22	−0.71	0.37	7.16	3.40	2.82
		2003	1093.07	490.16	491.27	3.48	27.97	22.35	918.49	0.23	−0.71	0.38	6.74	3.40	2.87
		2004	1141.61	503.94	520.54	3.34	29.55	25.20	969.91	0.23	−0.67	0.39	7.23	3.39	2.75
		2005	1106.73	498.20	497.64	3.42	28.01	23.38	912.28	0.24	−0.69	0.39	6.69	3.38	2.84
		2006	1060.20	524.29	461.13	3.19	25.02	21.29	828.28	0.27	−0.64	0.44	5.51	3.29	2.88
		2007	1088.25	529.72	477.36	3.40	28.62	23.07	879.66	0.24	−0.69	0.40	6.16	3.40	2.78
		2008	1061.60	507.15	484.12	3.51	27.82	21.81	905.99	0.23	−0.71	0.38	6.38	3.42	2.81
		2009	1159.72	548.48	512.00	3.28	28.82	26.99	966.41	0.25	−0.65	0.41	6.65	3.42	2.72
		2010	1144.24	520.13	516.73	3.48	29.00	24.54	973.19	0.22	−0.70	0.37	7.01	3.47	2.76
		2011	1039.90	492.67	487.00	3.51	25.59	18.61	917.70	0.22	−0.71	0.38	6.52	3.44	2.89
		2012	1097.31	518.59	492.28	3.39	27.74	21.75	911.22	0.24	−0.68	0.40	6.41	3.47	2.86
		2013	1070.43	533.90	490.31	3.56	25.86	17.68	927.65	0.22	−0.71	0.37	6.22	3.52	2.91
		2014	1104.83	543.26	490.10	3.31	26.68	21.11	925.18	0.24	−0.66	0.40	6.20	3.47	2.84
		2015	1009.30	519.33	456.00	3.63	26.77	17.64	861.60	0.22	−0.74	0.37	5.93	3.50	2.91
		2016	1016.58	520.26	456.58	3.70	25.06	17.02	827.57	0.22	−0.75	0.37	5.64	3.54	2.98
		2017	1175.10	576.54	522.99	3.53	26.77	27.70	891.13	0.23	−0.71	0.39	6.64	3.42	2.89
		2018	1168.45	546.88	518.53	3.75	28.59	28.49	943.19	0.21	−0.75	0.35	6.94	3.45	2.80
		2019	1136.80	544.16	512.05	3.43	27.78	24.04	942.77	0.23	−0.69	0.39	6.57	3.46	2.81
SP	AB	1982	1154.82	564.15	540.58	3.47	27.54	27.55	920.54	0.23	−0.70	0.38	7.24	3.38	2.88
		1983	1114.02	523.56	513.24	3.30	27.61	24.93	934.12	0.24	−0.67	0.41	7.11	3.34	2.82
		1984	1000.30	538.83	455.06	3.33	23.77	20.63	722.25	0.25	−0.70	0.42	5.44	3.22	3.00
		1985	1167.35	635.97	522.46	3.26	26.10	28.39	754.71	0.25	−0.68	0.42	5.98	3.27	3.05
		1986	1128.94	583.59	494.03	3.21	28.13	25.80	826.14	0.26	−0.66	0.43	6.01	3.36	2.91
		1987	1176.86	576.86	502.74	2.99	29.20	26.23	872.23	0.28	−0.59	0.45	6.06	3.36	2.81
		1988	1165.03	579.88	512.99	3.26	28.87	26.45	852.46	0.25	−0.66	0.42	6.23	3.44	2.89
		1989	1087.40	517.17	478.62	3.11	27.72	22.88	852.00	0.27	−0.63	0.44	5.96	3.32	2.84
		1990	1043.00	484.15	488.36	3.51	27.27	21.18	846.12	0.23	−0.71	0.38	6.46	3.33	2.87
		1991	1033.41	445.45	483.65	3.48	26.59	19.12	887.26	0.22	−0.71	0.38	6.77	3.33	2.91
		1992	1183.42	595.33	509.17	3.20	30.79	27.93	857.86	0.26	−0.64	0.42	5.93	3.37	2.77
		1993	1266.47	607.71	556.74	2.99	31.44	31.35	949.43	0.27	−0.58	0.43	6.90	3.35	2.68
		1994	1136.70	556.20	501.35	3.05	28.17	24.42	878.91	0.28	−0.60	0.44	6.05	3.33	2.81
		1995	1037.73	490.92	463.13	2.90	25.89	21.16	821.26	0.29	−0.58	0.47	5.82	3.18	2.82
		1996	1112.39	551.31	466.59	2.89	29.98	24.01	859.99	0.29	−0.58	0.47	5.53	3.34	2.73
		1997	1159.57	530.88	508.39	3.13	30.61	25.16	945.41	0.25	−0.63	0.42	6.64	3.39	2.72
		1998	1134.02	504.02	500.80	3.28	30.46	23.82	937.90	0.24	−0.66	0.40	6.66	3.43	2.78
		1999	1153.66	523.65	505.97	3.03	28.37	24.54	937.54	0.27	−0.60	0.44	6.61	3.36	2.80
		2000	1091.97	496.99	478.72	2.98	27.17	22.44	900.43	0.28	−0.60	0.46	6.27	3.31	2.82
		2001	1198.10	496.39	536.83	3.08	30.28	26.71	1028.96	0.25	−0.61	0.42	7.62	3.38	2.73
		2002	1057.18	476.31	469.57	3.12	27.29	20.72	893.60	0.26	−0.63	0.43	6.24	3.34	2.82
		2003	1105.92	485.16	490.61	3.13	28.41	22.52	925.89	0.25	−0.63	0.43	6.60	3.37	2.81
		2004	1230.44	582.86	542.66	3.16	30.24	28.83	976.20	0.26	−0.62	0.42	6.87	3.45	2.75
		2005	1238.49	584.63	553.42	3.03	29.86	29.90	955.13	0.27	−0.59	0.43	6.93	3.37	2.72
		2006	1144.04	569.03	511.93	3.00	27.33	26.66	873.16	0.28	−0.60	0.45	6.17	3.29	2.80
		2007	1155.08	567.20	510.73	3.26	29.28	25.57	895.34	0.25	−0.65	0.41	6.15	3.44	2.82
		2008	1113.50	532.52	509.11	3.16	27.41	24.87	913.89	0.26	−0.63	0.42	6.55	3.32	2.80
		2009	1148.87	513.45	520.64	3.04	30.77	25.82	988.11	0.26	−0.60	0.42	7.01	3.34	2.62
		2010	1376.17	578.19	633.59	3.22	33.85	36.31	1132.20	0.23	−0.62	0.38	8.90	3.45	2.58
		2011	1114.22	538.88	508.64	3.32	27.53	24.30	906.86	0.24	−0.67	0.41	6.55	3.41	2.86
		2012	1117.22	540.36	503.16	3.16	29.00	24.42	904.83	0.26	−0.63	0.42	6.40	3.38	2.75
		2013	1011.62	467.80	481.58	3.47	27.10	19.18	902.88	0.22	−0.70	0.38	6.59	3.37	2.82
		2014	1064.41	515.08	502.61	3.49	27.17	21.25	935.95	0.22	−0.71	0.37	6.88	3.44	2.85
		2015	1067.93	514.56	499.44	3.44	27.75	21.83	923.38	0.22	−0.70	0.38	6.83	3.39	2.83
		2016	1079.08	582.00	484.57	3.15	25.24	24.13	779.20	0.28	−0.64	0.45	5.57	3.31	2.92
		2017	1257.74	661.74	574.28	3.13	28.59	33.59	875.56	0.26	−0.62	0.43	6.76	3.37	2.82
		2018	1267.76	665.60	576.51	3.37	29.47	33.58	904.36	0.25	−0.67	0.40	6.85	3.48	2.84
		2019	1196.51	598.47	548.19	3.28	28.55	28.97	916.41	0.25	−0.65	0.41	6.92	3.40	2.82
SP	CC	1982	1022.21	483.98	450.88	3.21	24.42	19.07	796.30	0.26	−0.65	0.45	5.61	3.48	2.94
		1983	982.56	426.37	450.41	3.06	24.19	17.94	828.81	0.27	−0.62	0.45	6.07	3.31	2.85
		1984	972.53	479.99	424.16	2.81	25.28	17.33	798.67	0.29	−0.57	0.48	4.98	3.24	2.80
		1985	857.41	398.27	391.02	3.31	22.85	12.58	719.93	0.23	−0.71	0.41	5.11	3.18	3.04
		1986	924.24	428.53	415.10	3.17	23.48	14.60	780.51	0.25	−0.67	0.44	5.50	3.33	2.96
		1987	1052.44	475.21	462.85	3.46	27.20	18.68	879.78	0.23	−0.71	0.39	6.15	3.50	2.98
		1988	936.34	400.95	438.92	3.37	24.89	15.01	830.89	0.24	−0.69	0.40	6.01	3.29	2.91
		1989	941.13	421.49	426.12	3.24	24.07	14.70	803.37	0.26	−0.66	0.43	5.48	3.34	2.95
		1990	968.83	451.06	428.16	3.24	25.70	16.33	805.51	0.26	−0.66	0.43	5.47	3.34	2.91
		1991	945.38	461.25	416.65	3.12	24.59	15.04	782.24	0.27	−0.64	0.45	5.12	3.32	2.94
		1992	1136.65	488.01	504.14	3.24	29.39	23.53	963.70	0.24	−0.65	0.41	6.91	3.46	2.79
		1993	1176.43	469.31	546.07	3.13	30.57	26.15	1057.87	0.24	−0.62	0.40	7.91	3.37	2.65
		1994	1088.89	429.43	518.09	3.30	27.98	22.18	995.39	0.23	−0.66	0.39	7.60	3.35	2.76
		1995	944.31	411.39	429.29	3.02	23.57	15.66	812.19	0.28	−0.61	0.46	5.68	3.26	2.88
		1996	1095.09	497.12	478.04	2.97	29.22	23.10	916.19	0.28	−0.59	0.45	6.25	3.36	2.70
		1997	1082.55	493.70	476.52	3.32	29.04	21.29	909.53	0.24	−0.67	0.40	6.28	3.46	2.83
		1998	1080.81	470.90	480.41	3.32	29.35	21.57	918.70	0.24	−0.67	0.41	6.42	3.48	2.75
		1999	1066.87	471.82	473.64	3.25	27.02	19.90	904.41	0.25	−0.66	0.43	6.28	3.50	2.85
		2000	1180.25	524.27	518.89	3.16	30.24	26.60	1001.11	0.26	−0.63	0.42	6.92	3.51	2.71
		2001	1165.07	553.45	497.10	3.08	30.51	25.72	955.05	0.26	−0.62	0.44	6.22	3.50	2.72
		2002	1168.30	472.00	532.22	3.22	29.60	25.11	1031.77	0.23	−0.64	0.40	7.64	3.52	2.72
		2003	1099.34	524.98	459.35	2.95	27.99	21.31	871.40	0.28	−0.60	0.47	5.45	3.52	2.82
		2004	1098.75	557.59	468.01	3.10	27.23	22.27	844.59	0.27	−0.62	0.45	5.26	3.49	2.87
		2005	1071.45	545.81	454.54	3.02	26.42	21.74	818.61	0.28	−0.61	0.46	5.06	3.42	2.86
		2006	1019.06	480.55	467.22	3.08	22.92	18.50	857.03	0.26	−0.62	0.45	5.77	3.41	2.94
		2007	994.93	458.94	450.78	3.19	25.82	17.58	849.43	0.26	−0.65	0.43	5.77	3.36	2.86
		2008	1008.25	459.20	463.42	3.12	25.27	18.98	880.23	0.25	−0.64	0.43	6.11	3.36	2.85
		2009	1006.79	483.61	456.09	3.01	24.82	18.65	864.53	0.27	−0.61	0.46	5.72	3.38	2.83
		2010	1053.09	486.48	473.25	3.02	25.92	21.11	895.23	0.26	−0.63	0.44	6.34	3.29	2.87
		2011	1055.82	478.43	481.66	3.20	26.20	19.66	919.82	0.24	−0.65	0.41	6.28	3.43	2.84
		2012	1098.58	513.32	498.55	3.24	27.27	21.60	945.55	0.24	−0.64	0.41	6.24	3.49	2.80
		2013	993.18	456.03	470.05	3.34	23.35	16.44	896.67	0.23	−0.68	0.40	6.21	3.42	2.91
		2014	1057.14	480.29	488.88	3.31	25.81	20.07	937.48	0.23	−0.67	0.40	6.56	3.42	2.86
		2015	1017.02	476.85	466.27	3.25	24.92	18.55	888.35	0.25	−0.66	0.42	6.02	3.39	2.89
		2016	958.72	441.89	448.19	3.55	24.11	15.05	848.21	0.22	−0.73	0.39	6.01	3.43	2.98
		2017	920.05	435.16	433.41	3.32	23.33	14.28	812.05	0.24	−0.69	0.41	5.64	3.33	2.92
		2018	1007.02	480.31	467.83	3.51	23.94	17.51	872.86	0.22	−0.72	0.39	6.07	3.48	3.02
		2019	1106.79	524.27	494.54	3.37	27.18	21.14	928.28	0.23	−0.68	0.40	6.21	3.55	2.89
SP	UL	1982	902.88	385.88	411.65	3.03	20.91	14.81	777.95	0.26	−0.64	0.47	5.72	3.35	2.90
		1983	984.07	380.71	463.40	3.39	25.04	16.88	885.03	0.22	−0.69	0.40	6.77	3.36	2.85
		1984	1002.22	427.71	443.14	3.07	26.46	17.55	841.85	0.26	−0.62	0.43	5.96	3.16	2.73
		1985	1001.48	418.53	468.40	3.10	25.91	18.94	896.33	0.25	−0.62	0.43	6.45	3.34	2.68
		1986	1058.63	433.44	487.89	3.21	26.46	20.64	937.39	0.24	−0.64	0.42	6.85	3.47	2.71
		1987	1249.05	493.46	566.36	3.11	32.15	28.91	1103.54	0.24	−0.61	0.40	8.23	3.43	2.61
		1988	1095.41	471.79	490.19	3.20	29.22	22.23	940.43	0.24	−0.64	0.41	6.62	3.42	2.70
		1989	1234.38	478.03	568.09	3.25	31.82	27.87	1107.91	0.23	−0.64	0.38	8.32	3.44	2.61
		1990	1235.61	458.16	573.82	3.10	31.06	27.79	1121.14	0.23	−0.60	0.38	8.62	3.29	2.57
		1991	1369.29	481.41	652.18	3.05	33.81	34.53	1290.98	0.22	−0.58	0.37	10.20	3.42	2.49
		1992	1380.70	515.31	636.82	2.97	34.94	35.24	1256.37	0.24	−0.57	0.39	9.64	3.43	2.40
		1993	1356.89	513.05	622.04	2.94	34.51	33.97	1224.25	0.24	−0.56	0.40	9.33	3.42	2.46
		1994	1380.42	498.08	647.02	2.99	34.88	35.24	1279.47	0.24	−0.57	0.38	10.01	3.41	2.44
		1995	1342.78	460.85	644.09	3.11	34.27	33.80	1274.32	0.22	−0.60	0.36	10.27	3.36	2.46
		1996	1380.97	491.58	650.19	3.06	35.25	35.24	1286.40	0.23	−0.58	0.37	10.13	3.40	2.41
		1997	1344.70	469.67	627.27	3.13	33.44	32.61	1237.71	0.22	−0.60	0.37	9.89	3.37	2.52
		1998	1399.80	505.98	648.57	2.98	35.91	36.15	1283.30	0.24	−0.57	0.38	10.05	3.42	2.40
		1999	1358.32	476.13	640.70	3.04	34.16	34.24	1267.08	0.23	−0.58	0.38	10.10	3.41	2.46
		2000	1260.11	469.79	582.21	2.96	31.24	29.42	1141.61	0.25	−0.58	0.41	8.81	3.32	2.56
		2001	1372.54	493.89	640.90	3.05	34.90	34.76	1266.64	0.23	−0.58	0.38	9.96	3.42	2.47
		2002	1400.01	494.60	660.23	3.06	35.30	36.11	1308.15	0.23	−0.58	0.37	10.33	3.44	2.41
		2003	1373.90	474.73	651.02	2.99	34.12	35.01	1289.91	0.23	−0.57	0.38	10.35	3.36	2.43
		2004	1357.27	479.85	644.72	3.04	34.23	34.74	1274.38	0.23	−0.58	0.38	10.11	3.42	2.42
		2005	1368.10	493.89	643.71	3.05	34.91	34.76	1272.27	0.23	−0.58	0.38	9.92	3.43	2.44
		2006	1316.87	498.32	603.56	3.03	32.96	31.39	1185.54	0.24	−0.59	0.39	8.99	3.40	2.53
		2007	1364.36	485.22	640.04	3.04	34.07	34.18	1265.18	0.23	−0.58	0.38	9.97	3.39	2.45
		2008	1241.80	466.43	588.74	3.02	30.69	29.27	1154.83	0.24	−0.59	0.40	8.80	3.38	2.54
		2009	1289.58	476.77	612.29	2.97	32.19	31.23	1205.25	0.24	−0.57	0.40	9.23	3.36	2.49
		2010	1407.93	490.34	669.00	3.04	35.17	36.16	1327.05	0.22	−0.58	0.36	10.52	3.40	2.40
		2011	1266.10	464.00	595.15	3.01	31.14	29.99	1168.21	0.24	−0.59	0.40	9.11	3.36	2.58
		2012	1271.39	471.30	596.89	3.02	30.82	29.64	1172.22	0.24	−0.58	0.40	8.94	3.40	2.56
		2013	1379.00	495.27	648.90	3.05	34.89	34.87	1283.23	0.23	−0.58	0.37	10.00	3.45	2.45
		2014	1393.96	497.85	660.92	3.04	33.45	34.69	1309.06	0.22	−0.58	0.37	10.16	3.46	2.49
		2015	1432.87	503.46	672.07	3.04	35.17	36.79	1333.06	0.22	−0.57	0.37	10.52	3.48	2.45
		2016	1384.54	510.23	645.35	2.95	33.64	34.68	1276.30	0.24	−0.56	0.39	9.79	3.43	2.47
		2017	1452.66	488.12	697.42	2.92	36.61	39.16	1389.81	0.23	−0.55	0.37	11.26	3.37	2.31
		2018	1453.60	498.25	690.47	3.01	36.89	38.80	1373.71	0.22	−0.57	0.36	11.01	3.43	2.34
		2019	1417.09	496.68	667.06	2.87	34.76	37.03	1324.48	0.23	−0.55	0.39	10.52	3.35	2.40
SP	LL	1982	1045.12	441.92	457.77	3.32	25.08	17.79	869.89	0.24	−0.67	0.43	6.14	3.52	2.99
		1983	1071.25	438.36	483.81	3.22	26.66	20.20	924.47	0.24	−0.65	0.42	6.73	3.46	2.82
		1984	1056.41	469.76	457.02	3.27	26.79	18.75	866.98	0.24	−0.66	0.41	5.90	3.36	2.88
		1985	965.00	437.63	425.92	3.33	22.55	15.36	790.82	0.23	−0.72	0.42	5.72	3.37	3.22
		1986	1030.80	471.73	447.09	3.44	25.96	16.99	841.84	0.24	−0.70	0.41	5.60	3.52	3.06
		1987	1047.07	469.87	448.15	3.51	26.60	17.42	847.55	0.23	−0.72	0.40	5.77	3.54	3.09
		1988	1066.09	500.80	452.01	3.47	27.19	19.52	856.21	0.24	−0.70	0.41	5.59	3.53	3.02
		1989	1060.06	479.51	459.58	3.39	27.80	18.97	871.92	0.24	−0.68	0.41	5.77	3.51	2.94
		1990	1104.72	494.26	479.58	3.32	26.32	19.95	915.72	0.24	−0.67	0.41	6.09	3.50	2.95
		1991	1091.31	462.21	488.19	3.53	28.21	20.23	932.44	0.22	−0.71	0.38	6.64	3.54	2.94
		1992	1215.65	501.15	540.75	3.32	31.45	26.42	1038.08	0.23	−0.66	0.39	7.59	3.53	2.77
		1993	1195.57	485.47	540.35	3.40	30.65	25.51	1037.11	0.22	−0.67	0.38	7.64	3.53	2.80
		1994	1264.01	512.03	566.63	3.21	33.05	29.16	1100.04	0.24	−0.63	0.39	8.08	3.50	2.66
		1995	1156.14	457.57	532.22	3.39	30.84	24.60	1028.54	0.22	−0.68	0.37	7.68	3.46	2.74
		1996	1231.22	450.74	580.40	3.44	31.84	27.58	1133.92	0.21	−0.68	0.35	8.84	3.48	2.70
		1997	1170.96	511.82	507.93	3.28	30.24	24.12	975.81	0.24	−0.66	0.41	6.71	3.56	2.83
		1998	1120.10	522.61	471.31	3.12	28.92	21.60	897.76	0.26	−0.63	0.44	5.72	3.52	2.81
		1999	1131.68	496.45	486.45	3.33	28.94	22.18	930.65	0.24	−0.67	0.41	6.40	3.58	2.90
		2000	1127.72	481.97	499.16	3.38	28.99	22.40	958.11	0.23	−0.68	0.40	6.77	3.54	2.86
		2001	1168.78	472.90	532.53	3.41	29.18	23.97	1030.39	0.22	−0.68	0.38	7.58	3.53	2.85
		2002	1072.59	486.21	467.60	3.31	27.42	19.80	889.70	0.24	−0.67	0.41	5.91	3.51	2.93
		2003	1126.94	543.73	467.83	3.08	28.99	22.07	889.75	0.27	−0.62	0.45	5.44	3.56	2.83
		2004	1143.02	571.07	457.11	2.84	29.24	23.23	867.61	0.29	−0.56	0.49	4.96	3.56	2.75
		2005	1033.70	509.31	432.90	3.06	25.71	17.64	815.76	0.27	−0.62	0.46	4.79	3.50	2.93
		2006	1051.74	472.74	464.71	3.06	27.53	20.70	886.07	0.26	−0.60	0.45	5.82	3.39	2.77
		2007	1068.29	509.76	456.66	3.22	28.02	19.81	856.00	0.26	−0.65	0.43	5.38	3.52	2.88
		2008	1129.75	505.88	495.32	3.21	28.75	22.36	948.62	0.25	−0.64	0.42	6.32	3.52	2.84
		2009	1039.00	458.62	471.58	3.41	26.39	18.14	895.59	0.23	−0.69	0.39	6.04	3.47	2.91
		2010	1054.71	487.38	480.36	3.49	28.42	20.53	851.14	0.23	−0.70	0.38	5.95	3.45	2.86
		2011	1129.77	502.55	494.03	3.33	27.41	21.88	911.88	0.24	−0.67	0.41	6.37	3.55	2.94
		2012	1121.74	463.98	515.83	3.39	28.38	22.49	988.11	0.22	−0.68	0.39	7.12	3.51	2.82
		2013	1021.16	418.38	481.10	3.62	26.32	17.49	910.50	0.21	−0.73	0.36	6.71	3.47	2.95
		2014	1052.97	440.00	483.42	3.44	27.43	19.32	918.95	0.22	−0.69	0.39	6.57	3.46	2.85
		2015	1159.27	492.83	524.93	3.38	29.07	23.70	990.91	0.23	−0.67	0.39	7.10	3.54	2.84
		2016	1144.12	490.76	504.43	3.22	28.65	23.10	968.87	0.25	−0.64	0.42	6.72	3.54	2.81
		2017	1128.36	458.15	522.45	3.31	28.62	23.02	1008.18	0.23	−0.66	0.39	7.37	3.46	2.76
		2018	1109.79	444.68	517.84	3.47	29.06	22.21	999.69	0.22	−0.69	0.37	7.42	3.44	2.80
		2019	1044.45	474.17	458.53	3.28	27.82	18.93	870.72	0.25	−0.66	0.42	5.79	3.44	2.86
FA	SL	1986	1144.68	610.45	496.26	2.52	25.24	28.28	790.20	0.32	−0.50	0.52	5.53	3.32	2.76
		1987	1210.29	677.85	520.33	2.55	26.10	27.70	846.45	0.31	−0.49	0.51	5.30	3.38	2.73
		1988	1007.14	565.25	424.54	2.23	22.84	21.49	684.68	0.35	−0.45	0.58	4.26	3.12	2.75
		1989	1237.05	641.12	543.15	2.50	27.66	31.06	882.32	0.31	−0.49	0.51	6.18	3.27	2.67
		1990	1133.59	604.88	496.50	2.56	25.26	26.70	783.32	0.32	−0.50	0.51	5.38	3.28	2.72
		1991	1267.74	726.25	498.02	2.34	30.35	34.30	776.40	0.34	−0.47	0.54	4.80	3.31	2.69
		1992	1203.81	650.86	515.30	2.27	27.14	31.30	781.71	0.36	−0.43	0.56	5.36	3.20	2.64
		1993	1052.36	551.50	427.88	2.36	21.80	20.95	710.86	0.31	−0.50	0.53	4.77	3.04	2.89
		1994	1080.03	558.05	446.38	2.29	25.05	25.11	788.72	0.35	−0.46	0.56	5.04	3.15	2.66
		1995	1197.99	576.06	517.00	2.51	27.74	28.46	919.93	0.31	−0.49	0.50	6.36	3.24	2.61
		1996	1117.09	585.78	460.78	2.14	26.17	24.53	818.22	0.36	−0.42	0.57	4.99	3.17	2.65
		1997	1243.35	599.66	533.50	2.39	29.64	29.94	971.41	0.32	−0.46	0.52	6.56	3.34	2.50
		1998	1319.18	713.79	588.39	2.76	27.80	26.27	1057.17	0.29	−0.50	0.45	6.33	3.46	2.67
		1999	1055.82	517.74	454.53	2.33	24.26	23.04	824.22	0.34	−0.47	0.55	5.55	3.20	2.67
		2000	1139.19	530.31	505.53	2.43	25.94	26.68	913.53	0.32	−0.48	0.52	6.55	3.26	2.59
		2001	991.69	500.71	422.37	2.40	23.00	20.64	761.26	0.29	−0.55	0.52	5.39	3.05	2.92
		2002	1137.07	592.85	472.36	2.48	27.03	24.94	839.11	0.32	−0.50	0.53	5.29	3.35	2.69
		2003	1090.06	547.70	462.16	2.47	25.69	24.51	835.40	0.31	−0.52	0.52	5.70	3.19	2.73
		2004	1114.20	538.24	481.53	2.56	25.06	25.20	881.30	0.31	−0.51	0.52	6.05	3.29	2.65
		2005	1242.85	653.63	536.49	2.63	28.09	29.98	907.39	0.31	−0.50	0.50	6.00	3.40	2.60
		2006	967.00	472.97	430.25	2.56	21.42	21.42	761.11	0.27	−0.59	0.50	5.87	3.02	2.99
		2007	1013.57	533.56	424.36	2.53	23.16	22.26	736.14	0.28	−0.58	0.51	5.21	3.06	3.02
		2008	1372.49	761.09	575.30	2.50	31.87	36.31	895.49	0.31	−0.48	0.49	5.96	3.37	2.67
		2009	1031.73	534.85	436.21	2.47	24.10	22.68	767.09	0.30	−0.54	0.52	5.30	3.15	2.84
		2010	1265.78	627.91	542.43	2.58	30.35	33.42	929.51	0.30	−0.51	0.49	6.68	3.36	2.60
		2011	1292.68	734.91	560.47	2.52	27.72	37.62	733.79	0.31	−0.50	0.52	5.67	3.35	2.72
		2012	969.84	516.89	419.26	2.38	21.08	21.35	676.73	0.31	−0.52	0.55	4.87	3.11	2.85
		2013	1066.56	544.12	468.77	2.61	23.92	24.94	787.30	0.31	−0.53	0.52	5.63	3.30	2.67
		2014	1119.27	589.26	459.73	2.46	26.65	25.71	797.89	0.32	−0.51	0.53	5.24	3.28	2.69
		2015	1089.27	606.26	442.68	2.47	26.10	25.73	768.46	0.32	−0.52	0.54	4.76	3.30	2.70
		2016	1244.75	666.83	536.77	2.67	28.58	33.23	828.66	0.29	−0.54	0.49	6.04	3.41	2.69
		2017	1457.11	864.21	640.31	2.72	30.40	43.50	792.51	0.30	−0.51	0.48	6.00	3.42	2.75
		2018	1334.17	723.16	582.52	2.89	29.14	38.10	915.10	0.28	−0.57	0.47	6.68	3.35	2.71
		2019	1237.88	719.58	533.08	2.70	27.40	30.81	804.09	0.31	−0.53	0.49	5.39	3.34	2.79
FA	GB	1982	869.25	410.19	388.44	2.80	21.45	14.20	717.72	0.29	−0.60	0.49	4.98	3.18	2.87
		1983	778.41	405.00	334.36	2.59	17.59	11.28	590.82	0.29	−0.61	0.52	4.14	2.91	3.15
		1984	880.02	435.37	376.41	2.74	21.54	14.11	681.31	0.29	−0.60	0.49	4.62	3.08	2.98
		1985	868.30	426.26	375.69	2.63	20.70	15.47	693.19	0.29	−0.59	0.51	4.89	3.10	2.93
		1986	867.65	419.66	375.78	2.65	20.32	15.11	696.01	0.28	−0.61	0.51	4.97	3.06	3.00
		1987	756.87	390.65	324.49	2.45	16.21	10.41	588.34	0.29	−0.59	0.54	4.14	2.98	3.06
		1988	857.97	418.76	369.39	2.69	20.49	13.34	685.66	0.29	−0.59	0.51	4.65	3.17	2.94
		1989	1031.58	435.45	467.90	2.64	24.98	21.63	895.90	0.28	−0.55	0.48	6.68	3.18	2.69
		1990	797.48	392.67	351.89	2.67	17.57	11.58	639.20	0.27	−0.62	0.50	4.63	2.99	3.08
		1991	832.14	410.37	371.33	2.60	18.92	13.42	656.46	0.29	−0.59	0.51	4.78	2.94	3.08
		1992	850.88	432.57	367.89	2.62	19.73	14.26	647.10	0.29	−0.59	0.52	4.57	3.02	3.01
		1993	836.09	405.22	353.19	2.53	17.76	12.61	638.41	0.29	−0.56	0.54	4.44	3.18	2.84
		1994	942.16	442.88	397.37	2.93	20.48	17.88	721.02	0.29	−0.62	0.49	5.07	3.11	2.93
		1995	751.40	373.65	326.60	2.50	15.63	10.09	597.62	0.28	−0.61	0.53	4.43	2.90	3.18
		1996	762.67	386.51	326.40	2.52	15.61	10.30	595.50	0.29	−0.60	0.53	4.20	2.97	3.18
		1997	840.81	432.18	352.98	2.59	18.70	13.37	649.18	0.29	−0.60	0.52	4.49	3.07	3.06
		1998	848.38	417.47	368.22	2.76	18.36	13.90	678.46	0.28	−0.62	0.51	4.81	3.13	3.09
		1999	827.66	409.55	358.67	2.63	17.94	12.58	666.56	0.28	−0.60	0.51	4.69	3.13	3.05
		2000	866.93	436.39	364.12	2.48	19.70	15.48	676.79	0.30	−0.56	0.55	4.52	3.21	2.80
		2001	814.54	400.97	352.27	2.64	18.72	12.76	651.37	0.27	−0.63	0.51	4.85	2.99	3.11
		2002	861.25	448.07	360.87	2.57	19.92	14.45	656.97	0.28	−0.60	0.52	4.61	3.06	3.06
		2003	739.74	383.72	318.28	2.58	15.64	9.76	571.79	0.28	−0.63	0.52	4.28	2.88	3.23
		2004	756.71	387.65	335.52	2.82	15.43	10.84	608.13	0.26	−0.67	0.50	4.66	2.98	3.21
		2005	786.39	411.21	336.72	2.63	16.76	11.71	613.10	0.28	−0.61	0.52	4.36	3.03	3.10
		2006	857.09	447.08	361.85	2.68	19.73	15.15	666.33	0.29	−0.59	0.53	4.45	3.26	2.84
		2007	811.43	428.64	353.93	2.81	18.30	13.50	645.35	0.27	−0.65	0.50	4.65	3.10	3.07
		2008	893.21	478.12	385.98	2.74	19.65	16.98	660.39	0.28	−0.62	0.50	4.85	3.10	3.09
		2009	906.66	448.27	400.34	2.89	20.81	17.04	728.92	0.28	−0.62	0.49	5.22	3.28	2.86
		2010	806.31	433.26	350.49	2.84	18.30	13.37	637.00	0.27	−0.66	0.49	4.57	3.05	3.12
		2011	902.15	464.38	394.93	2.74	20.82	17.37	722.80	0.29	−0.58	0.51	4.92	3.27	2.81
		2012	788.89	429.84	354.63	2.72	15.76	14.45	650.61	0.30	−0.59	0.55	4.33	3.31	2.77
		2013	878.61	451.88	389.33	2.90	19.22	17.45	723.32	0.28	−0.62	0.51	5.03	3.27	2.81
		2014	871.18	465.03	369.50	2.65	18.86	15.87	680.63	0.30	−0.58	0.53	4.48	3.30	2.87
		2015	842.99	446.85	368.01	2.75	18.94	15.52	667.49	0.28	−0.62	0.52	4.70	3.16	2.95
		2016	1043.96	567.29	461.71	2.77	23.35	25.00	734.40	0.30	−0.56	0.51	5.25	3.37	2.73
		2017	1025.38	577.66	453.84	2.74	22.05	24.73	686.06	0.30	−0.58	0.52	4.98	3.23	2.89
		2018	1068.55	579.35	464.60	3.04	22.32	26.71	762.84	0.28	−0.63	0.49	5.42	3.33	2.87
		2019	926.03	523.46	412.87	2.75	19.88	19.68	677.44	0.31	−0.58	0.52	4.77	3.11	2.92
FA	MG	1982	1145.63	582.99	478.42	2.56	25.59	27.73	851.85	0.33	−0.50	0.53	5.60	3.24	2.74
		1983	1121.62	566.77	480.23	2.71	24.23	28.80	847.38	0.32	−0.54	0.53	5.78	3.21	2.77
		1984	940.44	509.06	401.18	2.82	19.92	20.93	712.94	0.31	−0.59	0.52	4.72	3.02	2.94
		1985	1150.99	612.27	474.60	3.14	26.18	32.13	892.00	0.30	−0.63	0.49	5.80	3.34	2.76
		1986	1103.73	570.79	461.95	2.73	24.10	26.15	868.75	0.31	−0.56	0.51	5.43	3.28	2.84
		1987	989.36	505.07	408.12	2.65	23.79	18.77	757.04	0.31	−0.54	0.52	4.71	3.26	2.80
		1988	1039.63	521.16	446.49	2.92	24.66	24.50	839.55	0.29	−0.59	0.49	5.63	3.24	2.71
		1989	1042.80	486.93	470.82	3.03	24.27	24.16	888.68	0.27	−0.62	0.45	6.20	3.27	2.72
		1990	938.03	448.15	421.53	2.99	23.19	18.05	787.03	0.26	−0.64	0.45	5.67	3.12	2.93
		1991	994.68	474.75	446.97	3.01	24.46	19.05	819.33	0.28	−0.61	0.46	5.61	3.26	2.85
		1992	998.31	480.71	429.53	2.80	24.73	19.52	788.39	0.30	−0.57	0.49	5.31	3.16	2.83
		1993	1016.86	491.40	430.80	2.72	25.00	19.86	795.04	0.30	−0.56	0.49	5.27	3.18	2.78
		1994	978.17	477.77	417.31	2.62	23.11	18.35	765.47	0.32	−0.53	0.52	5.09	3.12	2.78
		1995	885.54	428.02	395.30	2.72	21.46	14.29	735.34	0.31	−0.56	0.50	4.94	3.07	2.86
		1996	1004.95	476.05	438.26	2.71	24.31	19.90	822.91	0.31	−0.55	0.50	5.61	3.20	2.71
		1997	1043.62	537.44	394.47	2.33	23.49	19.80	734.66	0.35	−0.47	0.56	4.38	3.03	2.77
		1998	994.93	493.21	402.21	2.45	21.97	19.69	752.42	0.35	−0.50	0.55	4.65	3.04	2.77
		1999	1011.61	517.90	439.68	3.04	21.96	24.71	840.71	0.29	−0.62	0.49	5.73	3.20	2.84
		2000	1024.70	475.65	448.10	2.88	24.38	20.96	855.82	0.28	−0.59	0.48	5.89	3.38	2.72
		2001	1079.64	524.63	463.97	3.08	25.35	23.68	879.79	0.27	−0.63	0.45	5.90	3.32	2.79
		2002	1074.44	538.39	441.35	2.70	25.63	22.94	818.15	0.31	−0.55	0.51	5.34	3.27	2.75
		2003	1078.74	551.28	449.68	2.84	25.00	25.42	851.68	0.30	−0.58	0.49	5.49	3.26	2.78
		2004	984.67	519.72	412.63	2.71	22.27	20.59	766.84	0.31	−0.57	0.52	4.91	3.15	2.89
		2005	1053.99	568.90	457.18	2.98	24.14	23.96	862.86	0.29	−0.60	0.47	5.54	3.25	2.78
		2006	965.23	532.32	427.01	2.80	20.88	19.92	776.54	0.31	−0.57	0.51	4.95	3.24	2.80
		2007	1001.85	536.13	442.65	3.08	21.82	22.15	821.39	0.29	−0.63	0.48	5.35	3.26	2.94
		2008	1032.67	503.31	470.94	2.95	24.79	23.02	893.50	0.28	−0.60	0.46	6.33	3.21	2.73
		2009	935.63	482.35	428.69	2.87	21.17	19.76	798.40	0.30	−0.59	0.49	5.64	3.13	2.83
		2010	1058.23	532.72	462.74	3.21	24.42	23.70	858.70	0.26	−0.66	0.44	6.10	3.30	2.89
		2011	925.13	488.23	414.17	2.73	21.24	17.35	780.73	0.31	−0.56	0.50	4.98	3.22	2.81
		2012	1167.93	634.15	484.43	2.83	27.13	26.76	916.36	0.29	−0.57	0.48	5.24	3.46	2.65
		2013	1097.31	567.87	480.37	3.05	23.65	27.11	918.76	0.28	−0.61	0.46	5.89	3.40	2.66
		2014	1137.74	582.49	501.44	3.31	24.56	29.53	968.52	0.25	−0.67	0.43	6.54	3.41	2.79
		2015	1051.51	564.59	437.43	2.72	23.08	25.13	818.01	0.32	−0.55	0.52	5.01	3.21	2.78
		2016	978.41	526.26	425.61	2.89	22.54	20.92	780.39	0.30	−0.59	0.49	5.02	3.21	2.86
		2017	1149.93	656.64	512.35	2.88	23.26	30.45	752.43	0.30	−0.58	0.50	5.40	3.30	2.87
		2018	1322.01	729.18	573.38	3.12	26.97	37.91	959.16	0.27	−0.61	0.44	6.35	3.41	2.73
		2019	1229.10	686.45	520.21	3.03	26.53	33.80	869.92	0.29	−0.60	0.47	5.56	3.43	2.77
FA	SA	1982	1075.36	501.44	478.79	2.94	24.97	23.97	887.03	0.28	−0.59	0.47	6.36	3.27	2.75
		1983	1003.92	487.23	425.90	2.87	22.67	21.24	792.24	0.29	−0.59	0.50	5.38	3.34	2.77
		1984	1020.27	524.02	433.08	3.10	24.44	22.06	784.43	0.26	−0.65	0.45	5.37	3.24	2.85
		1985	1173.30	617.03	458.56	2.79	29.75	27.35	834.34	0.30	−0.56	0.48	4.95	3.35	2.66
		1986	1111.10	558.55	446.74	2.93	27.66	23.20	833.67	0.28	−0.59	0.47	5.05	3.41	2.71
		1987	1202.18	610.39	463.16	3.04	27.71	27.92	823.92	0.28	−0.60	0.45	5.11	3.27	2.81
		1988	1135.32	586.13	479.96	2.92	27.83	26.76	877.92	0.29	−0.59	0.48	6.06	3.31	2.77
		1989	1169.20	547.84	498.98	3.00	29.51	27.75	909.85	0.28	−0.59	0.45	6.23	3.40	2.64
		1990	1168.48	559.62	490.03	3.04	29.44	26.67	924.71	0.27	−0.60	0.44	6.16	3.30	2.67
		1991	1190.73	550.49	500.43	2.95	30.12	27.13	931.92	0.27	−0.59	0.44	6.28	3.36	2.64
		1992	1146.62	597.43	475.88	3.03	28.78	27.52	829.88	0.29	−0.61	0.47	5.52	3.33	2.73
		1993	1163.16	573.19	482.98	3.14	28.90	28.86	887.54	0.28	−0.63	0.45	6.00	3.32	2.65
		1994	1073.70	502.46	463.00	3.12	26.45	23.81	858.46	0.27	−0.63	0.45	5.92	3.31	2.74
		1995	939.91	432.00	418.58	3.13	23.96	16.73	785.93	0.26	−0.65	0.45	5.52	3.32	2.83
		1996	1077.90	483.64	472.29	3.18	25.61	23.01	900.05	0.26	−0.64	0.45	6.34	3.37	2.69
		1997	1067.45	489.49	448.54	3.15	27.20	21.56	847.18	0.26	−0.64	0.44	5.80	3.40	2.76
		1998	1152.62	535.34	482.43	3.09	27.92	27.65	919.27	0.28	−0.62	0.46	6.36	3.36	2.64
		1999	1158.16	561.40	495.51	3.31	27.01	28.65	948.04	0.28	−0.66	0.45	6.77	3.31	2.67
		2000	1141.78	499.49	492.38	2.73	27.89	26.10	950.16	0.29	−0.54	0.48	6.63	3.37	2.53
		2001	1213.69	532.74	523.12	3.11	29.59	29.76	1014.25	0.25	−0.62	0.43	7.29	3.43	2.61
		2002	1048.60	516.20	429.63	2.76	25.63	21.52	814.04	0.29	−0.58	0.50	5.36	3.33	2.73
		2003	1116.45	532.48	470.95	2.94	27.03	24.87	888.46	0.27	−0.60	0.46	6.13	3.33	2.71
		2004	1066.94	550.49	437.47	2.91	23.96	22.65	794.06	0.29	−0.60	0.48	5.19	3.23	2.88
		2005	1142.92	568.63	475.49	3.14	26.24	26.82	850.00	0.27	−0.64	0.45	5.83	3.30	2.81
		2006	1124.10	550.58	478.03	3.07	27.36	25.97	900.18	0.27	−0.62	0.45	5.98	3.37	2.68
		2007	1089.03	547.58	458.89	3.21	24.78	24.87	853.70	0.27	−0.65	0.45	5.62	3.31	2.83
		2008	1122.72	495.90	512.08	2.99	27.58	25.43	978.32	0.26	−0.59	0.44	7.08	3.36	2.63
		2009	1279.63	552.59	571.63	3.01	32.20	31.41	1104.42	0.26	−0.58	0.42	8.00	3.39	2.50
		2010	1172.46	552.61	502.28	3.11	27.54	28.24	944.27	0.26	−0.62	0.44	6.59	3.39	2.69
		2011	1213.85	599.33	499.95	2.64	32.05	28.38	959.61	0.30	−0.52	0.49	6.00	3.43	2.53
		2012	1199.41	587.90	513.79	2.97	29.56	28.34	974.71	0.27	−0.59	0.45	6.36	3.44	2.65
		2013	1153.53	559.85	507.63	3.03	26.57	26.42	966.32	0.26	−0.61	0.44	6.46	3.37	2.68
		2014	1132.80	589.85	468.66	2.88	25.27	23.48	895.28	0.29	−0.58	0.48	5.32	3.40	2.75
		2015	1207.23	624.85	505.86	3.40	25.47	31.57	948.00	0.26	−0.67	0.43	6.02	3.46	2.82
		2016	1018.19	552.41	433.60	2.77	21.78	22.58	755.64	0.32	−0.56	0.51	4.85	3.14	2.88
		2017	1225.35	575.79	537.90	3.12	27.72	31.99	967.98	0.27	−0.62	0.44	7.05	3.38	2.69
		2018	1234.02	560.10	542.66	3.37	28.60	31.48	1012.43	0.24	−0.67	0.39	7.36	3.40	2.70
		2019	1204.42	594.81	504.89	3.28	26.56	31.64	889.93	0.26	−0.66	0.43	6.16	3.37	2.77
FA	AB	1982	1172.41	546.70	496.45	2.57	26.07	26.98	873.91	0.30	−0.51	0.50	6.23	3.25	2.64
		1983	1125.35	562.50	478.44	2.77	24.27	26.79	825.02	0.29	−0.56	0.50	5.83	3.25	2.75
		1984	1099.00	611.14	453.51	2.79	23.51	26.99	693.98	0.29	−0.60	0.49	4.92	3.08	2.97
		1985	1193.35	628.85	489.18	2.70	29.80	29.47	825.62	0.30	−0.54	0.50	5.39	3.43	2.61
		1986	1064.84	542.75	437.35	2.60	27.12	22.65	787.64	0.31	−0.53	0.52	5.06	3.31	2.68
		1987	1140.56	552.09	453.84	2.58	27.15	23.45	819.90	0.30	−0.52	0.51	5.34	3.33	2.68
		1988	1105.70	553.04	457.70	2.47	26.91	24.04	812.86	0.33	−0.49	0.53	5.27	3.26	2.63
		1989	1209.94	534.95	530.39	2.74	29.91	28.60	970.95	0.28	−0.54	0.46	7.05	3.32	2.58
		1990	1231.38	595.63	504.83	2.66	29.94	29.02	904.64	0.30	−0.52	0.48	6.11	3.29	2.64
		1991	1082.31	513.81	462.98	2.88	28.54	22.53	841.33	0.28	−0.58	0.46	5.67	3.29	2.68
		1992	1229.51	626.60	511.14	2.73	30.45	29.91	836.42	0.30	−0.53	0.48	5.68	3.36	2.64
		1993	1360.57	668.22	557.82	2.54	33.82	35.05	967.01	0.31	−0.48	0.48	6.53	3.34	2.51
		1994	1104.65	527.62	464.83	2.74	27.68	23.07	835.27	0.30	−0.55	0.48	5.66	3.25	2.71
		1995	1031.95	481.17	447.03	2.74	25.47	20.53	824.92	0.30	−0.55	0.49	5.66	3.26	2.68
		1996	1104.49	505.08	477.57	2.74	27.71	23.81	884.81	0.30	−0.55	0.48	6.13	3.29	2.66
		1997	1204.68	587.00	480.92	2.62	30.82	26.84	895.20	0.31	−0.52	0.50	5.73	3.37	2.59
		1998	1114.58	534.32	451.38	2.55	27.26	22.66	840.59	0.31	−0.51	0.51	5.40	3.24	2.69
		1999	1287.82	593.34	536.83	2.69	32.23	31.92	1007.17	0.29	−0.52	0.47	6.89	3.36	2.56
		2000	1178.47	508.33	508.97	2.56	29.72	27.27	980.34	0.30	−0.51	0.49	6.90	3.27	2.55
		2001	1072.67	477.52	466.03	2.87	26.15	21.06	886.96	0.27	−0.59	0.46	6.38	3.25	2.79
		2002	1112.35	537.48	462.77	2.62	28.00	23.54	880.50	0.31	−0.53	0.50	5.68	3.33	2.63
		2003	1186.94	587.38	484.03	2.70	29.50	27.20	883.25	0.30	−0.54	0.49	5.76	3.34	2.64
		2004	1175.17	599.40	497.35	2.83	27.94	27.19	853.21	0.29	−0.57	0.47	5.74	3.36	2.76
		2005	1141.33	582.16	483.00	2.85	27.95	26.81	846.24	0.29	−0.57	0.48	5.66	3.34	2.69
		2006	1047.11	545.13	450.30	2.62	24.84	21.25	788.83	0.31	−0.53	0.51	5.00	3.29	2.72
		2007	1162.48	593.08	486.03	2.88	28.56	27.00	871.00	0.29	−0.57	0.47	5.64	3.33	2.71
		2008	1242.57	546.51	565.08	2.81	30.34	31.63	1036.04	0.27	−0.54	0.45	7.79	3.41	2.48
		2009	1163.32	524.97	515.58	2.65	28.87	26.89	959.01	0.30	−0.52	0.48	6.85	3.23	2.58
		2010	1153.45	545.08	497.74	2.77	27.67	25.71	907.44	0.27	−0.57	0.46	6.48	3.24	2.75
		2011	1165.35	546.40	503.99	2.84	29.50	26.76	937.16	0.28	−0.56	0.46	6.47	3.38	2.64
		2012	1213.00	531.00	543.45	2.86	30.32	28.77	1026.42	0.27	−0.56	0.45	7.36	3.39	2.58
		2013	1098.13	484.26	504.75	3.02	27.31	23.46	966.07	0.25	−0.60	0.43	7.03	3.37	2.64
		2014	1232.76	553.37	542.10	3.00	30.54	28.83	1051.94	0.26	−0.59	0.42	7.41	3.39	2.58
		2015	1248.24	649.10	527.12	2.72	29.56	31.52	880.29	0.30	−0.54	0.48	5.98	3.37	2.70
		2016	1208.37	672.58	524.80	2.71	26.11	30.98	762.87	0.31	−0.53	0.50	5.46	3.23	2.82
		2017	1204.55	648.39	537.01	2.90	27.25	30.47	820.10	0.29	−0.57	0.47	6.01	3.31	2.77
		2018	1243.80	635.80	565.93	3.05	28.36	32.89	900.21	0.27	−0.60	0.44	6.82	3.31	2.74
		2019	1134.49	550.99	498.15	2.87	27.82	25.60	898.01	0.28	−0.57	0.46	6.22	3.32	2.67
FA	CC	1982	798.39	393.99	350.43	2.42	15.95	12.62	638.71	0.31	−0.54	0.57	4.49	3.28	2.80
		1983	829.03	406.41	363.77	2.34	17.41	12.98	674.23	0.34	−0.49	0.58	4.43	3.22	2.72
		1984	878.69	481.88	349.68	2.24	20.09	15.24	641.14	0.34	−0.49	0.58	3.68	3.19	2.75
		1985	890.60	467.07	366.01	2.59	20.82	15.08	673.49	0.30	−0.57	0.54	4.17	3.35	2.79
		1986	779.79	395.88	329.30	2.46	16.07	11.44	606.26	0.30	−0.55	0.57	3.97	3.35	2.75
		1987	770.57	385.40	321.95	2.54	16.21	10.04	587.15	0.31	−0.54	0.58	3.76	3.56	2.65
		1988	913.12	429.08	400.94	2.65	21.01	16.07	756.95	0.30	−0.55	0.53	5.06	3.38	2.59
		1989	1026.77	458.06	454.36	2.53	24.24	21.23	866.14	0.32	−0.51	0.53	6.01	3.26	2.62
		1990	1060.51	525.35	442.00	2.57	24.56	20.99	842.03	0.31	−0.52	0.52	5.07	3.35	2.72
		1991	1061.63	524.19	435.78	2.55	26.61	22.09	827.13	0.32	−0.52	0.53	5.14	3.37	2.65
		1992	989.39	476.60	409.97	2.60	23.51	18.40	773.78	0.30	−0.54	0.53	5.01	3.45	2.62
		1993	1065.58	479.40	465.95	2.78	25.67	22.13	891.05	0.29	−0.56	0.49	6.36	3.43	2.65
		1994	992.35	459.98	430.33	2.75	22.46	19.89	806.65	0.29	−0.56	0.52	5.52	3.49	2.62
		1995	1059.21	461.52	476.57	2.76	25.00	22.18	915.94	0.28	−0.55	0.49	6.44	3.36	2.61
		1996	1060.35	467.10	474.21	2.80	25.00	22.25	912.18	0.28	−0.57	0.48	6.52	3.37	2.60
		1997	1073.76	518.24	438.75	2.61	26.30	22.61	834.53	0.30	−0.54	0.51	5.37	3.38	2.63
		1998	921.38	433.40	398.81	2.54	21.54	17.30	753.25	0.31	−0.52	0.54	5.01	3.34	2.63
		1999	863.49	435.25	373.16	2.58	19.20	14.76	696.50	0.29	−0.57	0.54	4.66	3.26	2.81
		2000	1130.67	526.19	476.83	2.51	28.44	26.15	917.37	0.30	−0.50	0.52	6.04	3.52	2.41
		2001	972.84	477.79	415.03	2.67	22.47	18.60	785.87	0.29	−0.57	0.52	5.22	3.37	2.70
		2002	1034.91	498.11	426.51	2.81	23.45	19.87	805.42	0.28	−0.59	0.49	5.19	3.47	2.74
		2003	915.83	473.18	386.24	2.49	20.21	17.92	702.61	0.32	−0.52	0.56	4.44	3.39	2.60
		2004	994.04	497.64	426.92	2.76	22.34	19.37	764.58	0.28	−0.57	0.50	4.91	3.51	2.72
		2005	1016.79	517.60	433.61	2.75	23.99	20.18	794.12	0.29	−0.57	0.50	5.06	3.35	2.77
		2006	950.47	458.72	421.13	2.75	22.51	17.94	779.21	0.29	−0.56	0.50	5.16	3.38	2.68
		2007	947.38	496.79	402.90	2.76	22.99	17.94	730.04	0.30	−0.57	0.51	4.53	3.42	2.68
		2008	871.97	428.49	394.07	2.73	20.23	14.45	728.34	0.29	−0.56	0.52	4.87	3.45	2.64
		2009	1018.52	459.76	463.71	2.89	25.47	20.86	885.77	0.27	−0.59	0.46	6.27	3.37	2.64
		2010	1023.48	522.22	429.88	2.70	24.29	20.45	797.91	0.29	−0.56	0.51	5.01	3.47	2.61
		2011	1000.40	490.46	436.53	2.84	24.59	19.00	828.77	0.28	−0.58	0.48	5.37	3.40	2.70
		2012	979.61	498.21	421.49	2.71	21.74	17.53	797.95	0.30	−0.55	0.52	4.79	3.50	2.57
		2013	1067.58	504.69	477.20	3.11	25.74	22.46	916.90	0.25	−0.63	0.44	6.23	3.43	2.69
		2014	996.82	504.43	433.61	2.79	22.36	18.98	826.27	0.29	−0.57	0.50	5.24	3.48	2.70
		2015	1035.39	541.13	427.16	2.49	24.44	21.40	802.07	0.32	−0.50	0.54	4.72	3.44	2.59
		2016	865.20	438.95	385.06	2.73	18.42	13.05	715.73	0.30	−0.57	0.51	4.48	3.31	2.83
		2017	966.62	487.98	437.24	2.94	20.75	17.68	812.11	0.27	−0.61	0.47	5.22	3.45	2.80
		2018	1084.32	598.52	476.00	2.98	21.83	20.70	908.39	0.26	−0.61	0.45	5.26	3.48	2.75
		2019	1067.20	535.45	461.80	2.90	23.95	21.28	877.08	0.27	−0.58	0.47	5.32	3.52	2.67
FA	UL	1982	1016.72	417.21	463.28	2.88	24.09	19.77	882.34	0.27	−0.58	0.47	6.52	3.43	2.66
		1983	959.15	388.81	437.92	3.19	24.38	16.29	832.05	0.24	−0.65	0.43	6.12	3.35	2.80
		1984	1069.96	513.06	431.16	2.75	28.28	20.88	812.17	0.28	−0.57	0.47	5.17	3.24	2.72
		1985	1019.75	434.30	462.34	2.79	24.79	20.96	886.88	0.27	−0.57	0.47	6.45	3.29	2.61
		1986	1127.59	489.98	502.23	3.07	29.03	24.27	967.06	0.25	−0.61	0.43	6.76	3.49	2.63
		1987	1171.03	512.38	506.16	2.95	30.29	25.53	969.33	0.26	−0.59	0.44	6.75	3.43	2.63
		1988	1312.68	488.04	593.70	2.86	32.48	31.83	1167.18	0.25	−0.55	0.41	9.02	3.34	2.46
		1989	1367.89	506.64	624.31	2.85	34.50	34.41	1231.94	0.24	−0.54	0.40	9.48	3.34	2.40
		1990	1326.63	574.15	572.94	2.68	34.53	32.68	1118.82	0.28	−0.51	0.44	7.64	3.41	2.46
		1991	1393.90	520.03	642.12	2.94	35.75	36.25	1269.21	0.24	−0.56	0.39	9.78	3.46	2.41
		1992	1325.27	551.90	577.84	2.78	33.40	32.80	1125.21	0.27	−0.54	0.45	8.12	3.48	2.50
		1993	1436.63	526.92	655.87	2.74	35.74	37.91	1297.30	0.25	−0.52	0.41	10.09	3.35	2.34
		1994	1469.94	493.75	702.60	2.91	36.98	40.04	1400.79	0.23	−0.55	0.37	11.38	3.40	2.30
		1995	1455.42	486.34	692.42	2.93	36.29	39.18	1378.89	0.23	−0.55	0.37	11.25	3.37	2.30
		1996	1417.00	498.51	653.18	2.93	35.11	36.25	1294.47	0.23	−0.56	0.38	10.29	3.36	2.41
		1997	1456.59	531.80	663.10	2.79	37.06	38.85	1315.63	0.25	−0.53	0.40	10.25	3.39	2.31
		1998	1311.81	474.99	613.89	2.77	32.60	33.75	1212.39	0.26	−0.53	0.43	9.56	3.34	2.35
		1999	1280.92	488.89	582.47	2.78	31.76	31.43	1143.80	0.26	−0.54	0.43	8.76	3.35	2.44
		2000	1397.68	513.15	644.87	2.76	35.66	37.34	1277.40	0.25	−0.53	0.41	10.01	3.36	2.34
		2001	1335.99	481.62	619.24	2.88	33.25	33.38	1222.14	0.24	−0.56	0.41	9.62	3.33	2.46
		2002	1380.22	492.70	638.22	2.76	33.83	35.84	1264.22	0.25	−0.53	0.41	10.01	3.34	2.35
		2003	1195.61	467.29	544.39	2.70	28.91	28.35	1063.39	0.26	−0.56	0.45	8.22	3.24	2.63
		2004	1337.88	503.94	616.01	2.79	33.70	34.42	1215.09	0.25	−0.55	0.42	9.37	3.36	2.43
		2005	1409.31	506.08	659.41	2.84	35.82	37.48	1308.06	0.24	−0.54	0.40	10.27	3.39	2.30
		2006	1299.08	482.91	598.33	2.81	32.20	32.15	1177.77	0.25	−0.54	0.42	9.09	3.37	2.40
		2007	1362.90	512.48	624.04	2.79	33.86	34.80	1231.50	0.25	−0.54	0.42	9.45	3.38	2.40
		2008	1280.35	488.16	595.79	2.79	31.88	31.95	1171.84	0.26	−0.54	0.43	8.89	3.36	2.41
		2009	1426.93	499.04	673.00	2.88	35.92	38.04	1337.43	0.23	−0.54	0.38	10.59	3.35	2.31
		2010	1403.35	562.23	608.68	2.65	33.96	35.45	1166.33	0.27	−0.50	0.43	8.76	3.28	2.43
		2011	1309.46	487.61	611.40	2.84	32.56	32.79	1201.40	0.25	−0.55	0.42	9.29	3.36	2.45
		2012	1327.89	505.91	604.09	2.75	32.82	32.89	1189.07	0.26	−0.52	0.43	8.91	3.39	2.42
		2013	1407.29	497.05	661.09	2.88	34.45	36.23	1311.25	0.23	−0.54	0.39	10.30	3.37	2.38
		2014	1438.53	516.30	669.84	2.86	35.70	38.05	1330.14	0.24	−0.54	0.40	10.40	3.46	2.35
		2015	1473.81	523.14	685.08	2.80	37.14	40.09	1362.87	0.24	−0.52	0.39	10.76	3.41	2.27
		2016	1382.33	492.54	650.41	2.77	34.51	36.47	1286.93	0.25	−0.52	0.41	10.16	3.35	2.32
		2017	1428.90	492.64	679.57	2.86	35.77	38.39	1351.77	0.23	−0.54	0.38	10.77	3.39	2.30
		2018	1389.27	493.60	653.60	2.80	34.90	36.86	1294.95	0.24	−0.54	0.40	10.27	3.34	2.33
		2019	1351.28	521.73	616.73	2.76	34.46	35.27	1211.82	0.25	−0.54	0.42	9.22	3.40	2.40
FA	LL	1982	1152.97	469.79	521.34	2.86	26.94	24.97	1010.72	0.26	−0.56	0.45	7.33	3.38	2.65
		1983	1043.31	476.30	444.41	2.86	24.39	19.82	840.64	0.28	−0.58	0.48	5.67	3.43	2.76
		1984	1062.65	522.15	449.35	2.90	25.33	20.83	798.95	0.27	−0.60	0.47	5.33	3.33	2.89
		1985	1030.92	508.29	433.92	2.84	24.87	19.50	820.14	0.29	−0.58	0.49	5.05	3.46	2.77
		1986	1041.70	495.46	430.38	2.90	24.57	18.30	811.48	0.28	−0.59	0.48	5.12	3.43	2.83
		1987	1170.40	588.83	458.55	2.64	29.52	24.88	872.93	0.31	−0.53	0.51	5.02	3.55	2.66
		1988	1070.26	524.02	436.34	2.76	26.99	20.78	824.70	0.29	−0.56	0.49	5.01	3.44	2.72
		1989	1149.75	519.54	486.30	2.80	29.12	24.26	933.64	0.28	−0.55	0.47	6.17	3.38	2.66
		1990	1188.80	541.04	503.29	2.90	30.45	25.67	968.11	0.27	−0.57	0.44	6.34	3.45	2.63
		1991	1127.06	512.21	473.78	2.70	28.33	24.31	908.38	0.29	−0.54	0.50	6.09	3.56	2.49
		1992	1220.12	526.77	534.09	2.84	30.67	28.36	1013.77	0.27	−0.56	0.45	7.21	3.50	2.54
		1993	1231.19	517.18	542.84	2.81	30.95	29.12	1055.29	0.26	−0.56	0.44	7.52	3.42	2.53
		1994	1263.07	524.43	560.03	2.88	32.44	30.50	1088.79	0.26	−0.56	0.43	7.78	3.46	2.50
		1995	1270.01	503.18	573.74	2.97	32.90	30.60	1121.28	0.25	−0.58	0.41	8.32	3.46	2.50
		1996	1267.93	521.08	561.16	2.86	32.39	30.56	1088.84	0.26	−0.56	0.43	7.89	3.44	2.49
		1997	1225.33	595.59	495.21	2.47	32.07	30.16	949.16	0.32	−0.49	0.52	5.85	3.51	2.42
		1998	1258.70	562.58	525.79	2.69	32.17	29.28	1018.61	0.29	−0.53	0.47	6.77	3.49	2.55
		1999	1160.77	521.11	493.74	2.80	29.04	25.56	950.13	0.27	−0.57	0.46	6.39	3.44	2.68
		2000	1183.76	558.35	493.87	2.70	30.95	27.06	949.46	0.29	−0.54	0.48	6.06	3.47	2.55
		2001	1235.15	558.08	520.35	2.88	29.93	27.72	1006.15	0.27	−0.57	0.45	6.58	3.51	2.67
		2002	1149.24	560.98	464.80	2.58	28.67	24.64	882.01	0.31	−0.52	0.51	5.37	3.43	2.63
		2003	1167.40	587.31	449.02	2.44	29.26	25.10	852.88	0.32	−0.49	0.53	4.84	3.41	2.61
		2004	1050.44	557.72	412.40	2.58	27.53	21.61	775.10	0.32	−0.52	0.53	4.17	3.50	2.58
		2005	1114.96	562.58	449.84	2.68	29.55	24.08	854.16	0.30	−0.53	0.50	4.85	3.50	2.59
		2006	1130.12	562.15	452.67	2.56	28.55	23.21	860.36	0.31	−0.51	0.50	4.97	3.35	2.64
		2007	1187.91	561.61	499.63	2.81	30.10	26.49	958.06	0.29	−0.55	0.47	6.01	3.51	2.58
		2008	1133.08	530.73	483.08	2.80	27.45	24.02	914.69	0.28	−0.56	0.48	5.88	3.51	2.69
		2009	1066.46	505.19	454.20	2.70	25.29	21.43	865.36	0.29	−0.55	0.49	5.40	3.40	2.69
		2010	1269.73	652.00	515.12	2.74	28.86	30.01	828.98	0.29	−0.54	0.48	5.43	3.37	2.78
		2011	1084.41	476.38	484.54	2.93	26.71	22.26	920.64	0.27	−0.59	0.45	6.38	3.39	2.68
		2012	1049.30	475.08	462.72	3.04	27.04	20.47	880.47	0.26	−0.61	0.45	5.78	3.45	2.69
		2013	1103.39	471.71	498.69	3.04	28.12	23.22	949.23	0.25	−0.60	0.43	6.65	3.41	2.64
		2014	1112.95	484.10	500.78	3.12	27.64	22.31	951.96	0.25	−0.62	0.42	6.59	3.44	2.73
		2015	1178.42	513.18	517.15	2.82	29.02	26.49	989.09	0.27	−0.56	0.46	6.87	3.49	2.53
		2016	1145.34	525.45	491.39	2.86	28.07	24.55	944.70	0.28	−0.56	0.47	6.19	3.48	2.55
		2017	1162.07	501.09	515.08	2.87	29.41	25.61	995.86	0.27	−0.56	0.45	6.82	3.45	2.55
		2018	1142.62	527.09	489.23	2.80	28.93	24.73	934.73	0.27	−0.57	0.46	6.15	3.41	2.66
		2019	1125.94	547.43	466.99	2.83	29.79	23.63	890.08	0.29	−0.56	0.47	5.37	3.48	2.63

**TABLE A2:** Community‐weighted means for functional traits, part 2. Community‐weighted trait means for caudal fin shape (CF), body shape (BSh), body cross‐section shape (CrSec), piscivory (Pisc), invertivory (Invert), herbivory (Herb), detritivory (Detr), water column position (Pos), preferred temperature in degrees Celsius (Tp), minimum observed temperature in degrees Celsius (MinTp), maximum observed temperature in degrees Celsius (MaxTp), observed temperature range (TpRng), and salinity preference (Sal). Note that some variable abbreviations here differ from those listed in Table 4 to save space. Season abbreviations are SP: spring, FA: fall. Bay abbreviations are as follows, SL: Sabine Lake, GB: Galveston Bay, MG: Matagorda Bay, SA: San Antonio Bay, AB: Aransas Bay, CC: Corpus Christi Bay, UL: Upper Laguna Madre, LL: Lower Laguna Madre

Season	Bay	Year	CF	BSh	CrSec	Pisc	Invert	Herb	Detr	Pos	RepG	Tp	MinTp	MaxTp	TpRng	Sal
SP	SL	1986	2.47	2.53	2.47	0.87	0.89	0.13	0.18	4.00	0.76	20.15	16.98	27.34	10.36	1.85
		1987	2.08	2.58	2.53	0.88	0.89	0.12	0.07	4.41	0.27	19.50	15.03	27.08	12.05	1.73
		1988	2.72	2.81	2.73	0.77	0.80	0.22	0.13	3.95	0.56	21.30	15.47	27.20	11.73	1.92
		1989	2.75	2.98	2.86	0.74	0.77	0.25	0.11	3.94	0.38	21.64	14.84	27.19	12.35	1.98
		1990	2.40	2.74	2.68	0.81	0.85	0.18	0.09	4.25	0.31	20.50	15.11	27.20	12.09	1.87
		1991	2.30	2.73	2.60	0.84	0.87	0.14	0.08	4.27	0.29	19.61	15.02	27.25	12.23	1.74
		1992	2.94	2.93	2.83	0.80	0.82	0.19	0.15	3.88	0.65	22.37	15.80	27.26	11.46	2.22
		1993	2.48	2.95	2.79	0.84	0.88	0.14	0.09	4.32	0.28	21.25	14.28	27.17	12.88	2.11
		1994	3.02	3.03	2.89	0.74	0.79	0.25	0.17	3.77	0.55	22.21	14.94	27.23	12.29	2.13
		1995	2.63	2.91	2.82	0.76	0.82	0.21	0.09	4.13	0.25	20.95	15.04	27.18	12.14	1.95
		1996	2.33	2.89	2.80	0.77	0.81	0.22	0.05	4.30	0.07	20.63	14.03	27.17	13.14	1.84
		1997	2.56	3.01	2.86	0.77	0.83	0.21	0.09	4.20	0.23	21.46	14.33	27.18	12.85	2.06
		1998	2.55	2.94	2.83	0.78	0.83	0.21	0.08	4.20	0.29	21.54	14.52	27.15	12.63	2.09
		1999	2.81	2.98	2.86	0.75	0.80	0.24	0.11	3.99	0.48	21.83	15.23	27.18	11.95	2.09
		2000	3.18	3.17	3.05	0.60	0.73	0.40	0.19	3.58	0.36	21.96	15.52	27.14	11.62	2.04
		2001	2.81	3.04	2.89	0.80	0.84	0.19	0.09	4.15	0.51	22.46	15.09	27.17	12.08	2.29
		2002	3.18	3.11	2.93	0.75	0.83	0.24	0.24	3.62	0.78	23.41	15.98	27.27	11.30	2.47
		2003	3.04	2.97	2.81	0.76	0.81	0.22	0.14	3.84	0.80	22.36	15.95	27.29	11.34	2.16
		2004	2.99	3.12	2.93	0.76	0.83	0.22	0.11	4.01	0.62	22.97	15.45	27.27	11.82	2.37
		2005	2.65	3.11	2.90	0.77	0.81	0.22	0.09	4.14	0.31	22.01	13.99	27.22	13.22	2.15
		2006	3.23	3.12	2.95	0.69	0.75	0.30	0.16	3.68	0.71	22.84	15.45	27.23	11.79	2.20
		2007	2.96	3.00	2.85	0.83	0.88	0.16	0.15	4.08	0.78	23.27	16.01	27.31	11.30	2.54
		2008	3.22	3.16	2.97	0.73	0.78	0.26	0.14	3.83	0.74	23.34	15.59	27.27	11.68	2.39
		2009	2.73	3.05	2.87	0.79	0.88	0.20	0.14	4.16	0.50	22.51	15.61	27.31	11.70	2.41
		2010	2.56	3.02	2.88	0.87	0.91	0.12	0.09	4.41	0.44	23.17	15.41	27.30	11.90	2.59
		2011	2.89	3.05	2.90	0.78	0.82	0.21	0.09	4.16	0.60	23.02	15.45	27.27	11.82	2.37
		2012	2.76	3.00	2.88	0.81	0.87	0.18	0.14	4.10	0.53	22.78	15.94	27.25	11.31	2.43
		2013	2.76	2.99	2.81	0.85	0.89	0.14	0.11	4.25	0.73	23.11	15.41	27.30	11.89	2.50
		2014	2.51	2.83	2.72	0.87	0.90	0.12	0.11	4.29	0.57	22.11	15.64	27.32	11.67	2.29
		2015	2.31	2.88	2.74	0.88	0.92	0.10	0.08	4.46	0.39	21.80	15.08	27.23	12.16	2.31
		2016	2.14	2.66	2.54	0.94	0.96	0.05	0.06	4.62	0.52	20.75	15.34	27.30	11.97	2.14
		2017	2.46	2.87	2.67	0.85	0.89	0.14	0.10	4.30	0.54	21.20	15.12	27.31	12.19	2.11
		2018	2.54	2.86	2.69	0.87	0.92	0.12	0.13	4.28	0.63	21.94	15.59	27.36	11.77	2.32
		2019	2.21	2.77	2.59	0.92	0.94	0.07	0.05	4.63	0.46	20.95	14.95	27.36	12.42	2.15
SP	GB	1982	4.05	2.71	2.63	0.79	0.88	0.19	0.53	2.59	2.10	25.00	19.12	27.48	8.36	2.75
		1983	3.98	2.80	2.68	0.77	0.91	0.20	0.57	2.60	1.97	24.97	19.08	27.47	8.39	2.85
		1984	3.85	2.85	2.75	0.77	0.92	0.20	0.49	2.80	1.75	24.94	18.69	27.45	8.76	2.87
		1985	4.09	2.75	2.69	0.77	0.91	0.23	0.59	2.54	2.02	25.01	19.37	27.45	8.07	2.87
		1986	3.98	2.91	2.82	0.73	0.90	0.26	0.52	2.78	1.71	24.89	18.93	27.38	8.46	2.89
		1987	4.28	2.71	2.64	0.77	0.93	0.22	0.65	2.36	2.26	25.24	20.07	27.47	7.40	2.96
		1988	4.07	2.57	2.50	0.87	0.92	0.12	0.58	2.46	2.37	25.31	19.36	27.51	8.15	2.82
		1989	4.29	2.71	2.66	0.76	0.96	0.23	0.69	2.31	2.21	25.23	20.39	27.47	7.07	3.05
		1990	4.43	2.63	2.59	0.77	0.96	0.22	0.70	2.26	2.45	25.29	20.92	27.51	6.59	3.04
		1991	4.17	2.63	2.53	0.82	0.90	0.16	0.59	2.41	2.32	24.80	19.56	27.50	7.94	2.72
		1992	3.91	2.72	2.64	0.82	0.94	0.18	0.55	2.68	1.94	24.78	19.26	27.45	8.19	2.85
		1993	4.20	2.72	2.66	0.77	0.95	0.23	0.63	2.50	2.12	24.92	20.35	27.45	7.10	2.98
		1994	4.09	2.64	2.55	0.82	0.94	0.16	0.60	2.50	2.21	24.72	19.69	27.48	7.79	2.80
		1995	4.15	2.55	2.51	0.84	0.92	0.15	0.58	2.51	2.38	24.89	19.47	27.53	8.06	2.73
		1996	3.74	2.87	2.78	0.77	0.87	0.21	0.42	3.02	1.63	24.84	17.82	27.41	9.59	2.73
		1997	3.37	2.82	2.73	0.84	0.94	0.14	0.34	3.54	1.39	24.38	17.32	27.38	10.06	2.79
		1998	3.71	2.91	2.79	0.77	0.93	0.22	0.42	3.26	1.51	24.42	18.21	27.36	9.16	2.83
		1999	3.70	2.89	2.79	0.77	0.87	0.22	0.36	3.30	1.51	24.40	17.49	27.38	9.89	2.68
		2000	3.78	2.89	2.73	0.80	0.89	0.18	0.40	3.17	1.80	24.87	17.87	27.42	9.56	2.82
		2001	3.53	2.78	2.64	0.87	0.95	0.11	0.41	3.27	1.71	24.48	17.75	27.45	9.70	2.78
		2002	3.79	2.76	2.68	0.82	0.92	0.17	0.47	3.00	1.84	24.83	18.40	27.43	9.04	2.84
		2003	3.88	2.78	2.69	0.81	0.95	0.19	0.47	3.08	1.87	24.74	19.09	27.45	8.36	2.93
		2004	3.99	2.58	2.51	0.86	0.93	0.12	0.51	2.81	2.33	24.97	19.28	27.53	8.25	2.80
		2005	4.08	2.95	2.88	0.67	0.92	0.31	0.54	2.84	1.64	24.84	19.35	27.33	7.98	3.00
		2006	4.26	2.70	2.63	0.78	0.90	0.21	0.57	2.53	2.29	25.19	19.72	27.48	7.76	2.86
		2007	3.54	2.81	2.75	0.83	0.92	0.15	0.37	3.39	1.53	24.65	17.74	27.37	9.64	2.82
		2008	3.81	2.70	2.65	0.84	0.92	0.15	0.38	3.32	1.91	24.55	18.62	27.45	8.84	2.79
		2009	3.68	2.73	2.65	0.88	0.95	0.11	0.39	3.25	1.85	24.79	18.56	27.54	8.97	2.85
		2010	3.67	2.66	2.56	0.89	0.94	0.09	0.40	3.22	1.92	24.37	18.76	27.51	8.75	2.74
		2011	3.74	2.77	2.71	0.84	0.96	0.15	0.39	3.37	1.82	24.85	18.95	27.46	8.51	2.96
		2012	3.79	2.65	2.56	0.89	0.97	0.09	0.49	2.94	2.12	25.04	19.31	27.52	8.21	2.94
		2013	3.91	2.58	2.51	0.90	0.95	0.08	0.45	3.04	2.29	24.84	19.53	27.58	8.05	2.83
		2014	3.69	2.69	2.64	0.87	0.95	0.11	0.36	3.42	1.89	24.87	18.66	27.50	8.85	2.87
		2015	3.58	2.72	2.66	0.89	0.96	0.08	0.30	3.53	1.73	24.47	18.35	27.51	9.16	2.81
		2016	3.56	2.72	2.67	0.86	0.94	0.12	0.28	3.65	1.65	24.20	18.39	27.46	9.07	2.74
		2017	3.55	2.75	2.67	0.83	0.89	0.15	0.28	3.52	1.62	23.83	18.19	27.45	9.26	2.58
		2018	3.43	2.79	2.70	0.84	0.91	0.15	0.22	3.75	1.45	23.77	17.56	27.42	9.87	2.62
		2019	3.59	2.84	2.74	0.80	0.87	0.18	0.23	3.73	1.51	24.07	17.67	27.44	9.77	2.62
SP	MG	1982	3.36	2.82	2.72	0.78	0.83	0.21	0.21	3.56	1.24	22.99	16.73	27.42	10.69	2.24
		1983	3.26	3.10	2.96	0.69	0.81	0.26	0.20	3.68	0.85	23.92	15.82	27.26	11.44	2.51
		1984	4.27	2.67	2.62	0.74	0.84	0.24	0.33	3.14	2.26	24.60	18.96	27.55	8.59	2.53
		1985	3.77	2.70	2.69	0.71	0.86	0.28	0.39	3.15	1.52	23.17	18.46	27.44	8.98	2.37
		1986	3.73	2.82	2.76	0.77	0.83	0.22	0.34	3.08	1.57	24.58	17.48	27.40	9.92	2.56
		1987	3.27	2.77	2.70	0.84	0.90	0.15	0.30	3.52	1.32	23.98	17.04	27.41	10.37	2.59
		1988	3.68	2.89	2.81	0.76	0.89	0.22	0.28	3.47	1.52	24.70	17.60	27.42	9.82	2.77
		1989	3.91	2.78	2.72	0.76	0.83	0.22	0.34	3.05	1.83	24.85	17.86	27.48	9.62	2.59
		1990	3.95	2.71	2.61	0.77	0.85	0.22	0.41	2.94	1.99	24.41	18.24	27.55	9.32	2.50
		1991	3.98	2.63	2.54	0.84	0.90	0.15	0.47	2.75	2.18	24.68	18.88	27.60	8.72	2.65
		1992	3.51	2.72	2.71	0.79	0.86	0.19	0.34	3.24	1.42	23.91	17.46	27.44	9.98	2.46
		1993	3.82	2.62	2.59	0.82	0.89	0.16	0.42	2.99	1.90	24.15	18.53	27.50	8.97	2.55
		1994	3.90	2.83	2.77	0.72	0.79	0.27	0.29	3.23	1.58	23.89	17.11	27.41	10.30	2.36
		1995	3.78	2.91	2.87	0.67	0.81	0.32	0.35	3.21	1.18	23.39	16.94	27.38	10.44	2.36
		1996	3.99	2.83	2.77	0.74	0.81	0.23	0.41	2.80	1.79	24.99	18.00	27.44	9.44	2.61
		1997	3.43	2.96	2.86	0.73	0.86	0.24	0.29	3.57	1.00	23.49	15.99	27.34	11.35	2.48
		1998	3.69	2.85	2.77	0.76	0.84	0.21	0.26	3.46	1.44	23.99	17.06	27.38	10.32	2.51
		1999	3.99	2.90	2.82	0.72	0.79	0.26	0.32	3.03	1.69	24.77	17.34	27.41	10.07	2.52
		2000	3.78	2.91	2.76	0.75	0.84	0.19	0.23	3.30	1.66	24.28	17.45	27.48	10.03	2.52
		2001	3.92	2.76	2.70	0.78	0.85	0.21	0.37	3.11	1.85	24.80	17.77	27.50	9.73	2.63
		2002	3.93	2.73	2.62	0.82	0.89	0.15	0.39	3.04	2.05	24.84	18.39	27.55	9.16	2.71
		2003	3.55	2.79	2.69	0.83	0.94	0.13	0.33	3.43	1.66	24.72	17.77	27.53	9.75	2.81
		2004	3.47	2.77	2.74	0.82	0.90	0.17	0.28	3.58	1.35	23.92	16.81	27.46	10.65	2.59
		2005	3.79	2.69	2.67	0.82	0.92	0.17	0.37	3.28	1.81	24.63	17.96	27.54	9.57	2.76
		2006	3.79	2.85	2.80	0.76	0.84	0.22	0.25	3.46	1.51	24.03	17.50	27.43	9.93	2.51
		2007	3.67	2.85	2.79	0.76	0.84	0.23	0.22	3.67	1.38	23.72	16.96	27.42	10.46	2.47
		2008	3.67	2.82	2.77	0.83	0.90	0.13	0.24	3.45	1.61	24.71	17.96	27.59	9.63	2.71
		2009	4.13	2.89	2.79	0.70	0.81	0.24	0.23	3.41	1.91	24.71	18.41	27.49	9.07	2.63
		2010	3.85	2.65	2.54	0.89	0.95	0.09	0.30	3.45	2.18	24.81	18.64	27.61	8.97	2.84
		2011	4.01	2.64	2.57	0.85	0.90	0.14	0.23	3.66	2.23	24.91	18.64	27.54	8.89	2.78
		2012	3.73	2.67	2.57	0.88	0.95	0.09	0.29	3.61	2.03	24.72	18.49	27.61	9.11	2.85
		2013	3.87	2.71	2.56	0.89	0.94	0.08	0.24	3.68	2.18	24.83	18.61	27.63	9.02	2.84
		2014	4.01	2.59	2.52	0.92	0.96	0.06	0.21	3.70	2.35	25.00	19.16	27.69	8.53	2.90
		2015	3.44	2.83	2.76	0.82	0.94	0.10	0.21	3.59	1.47	24.37	18.39	27.59	9.19	2.77
		2016	3.91	2.83	2.75	0.77	0.82	0.21	0.21	3.52	1.73	24.28	17.73	27.47	9.74	2.52
		2017	3.60	2.79	2.74	0.82	0.87	0.16	0.16	3.74	1.54	24.08	17.51	27.53	10.02	2.58
		2018	3.60	2.68	2.59	0.87	0.92	0.11	0.18	3.83	1.84	24.20	17.93	27.57	9.64	2.65
		2019	3.56	2.77	2.71	0.79	0.82	0.20	0.17	3.68	1.47	23.51	17.30	27.45	10.16	2.36
SP	SA	1982	3.86	2.62	2.53	0.85	0.96	0.12	0.37	3.36	2.16	24.63	19.07	27.55	8.48	2.82
		1983	3.87	2.65	2.56	0.84	0.94	0.12	0.29	3.64	2.11	24.56	18.83	27.53	8.70	2.80
		1984	4.21	2.49	2.47	0.80	0.92	0.15	0.45	2.83	2.53	24.69	20.43	27.62	7.20	2.68
		1985	3.99	2.34	2.32	0.88	1.00	0.10	0.50	3.01	2.55	24.18	21.01	27.61	6.60	2.76
		1986	3.72	2.50	2.47	0.90	0.99	0.07	0.42	3.29	2.11	24.69	19.30	27.49	8.19	2.87
		1987	3.25	2.64	2.62	0.85	0.95	0.13	0.38	3.51	1.40	24.39	17.19	27.40	10.21	2.72
		1988	3.23	2.63	2.58	0.88	0.97	0.10	0.33	3.59	1.45	23.76	17.78	27.48	9.71	2.65
		1989	3.79	2.47	2.44	0.90	0.94	0.09	0.49	2.83	2.18	24.44	19.35	27.54	8.20	2.67
		1990	4.09	2.41	2.37	0.89	0.94	0.09	0.55	2.63	2.58	24.81	19.93	27.62	7.68	2.72
		1991	3.91	2.49	2.41	0.90	0.96	0.09	0.47	2.94	2.31	24.52	19.22	27.57	8.35	2.73
		1992	3.30	2.68	2.55	0.91	0.97	0.08	0.32	3.63	1.62	23.93	17.81	27.55	9.75	2.69
		1993	3.30	2.80	2.60	0.90	0.97	0.09	0.31	3.69	1.54	24.08	17.08	27.54	10.47	2.74
		1994	3.70	2.78	2.65	0.83	0.88	0.16	0.32	3.40	1.77	24.50	17.64	27.48	9.85	2.65
		1995	3.83	2.73	2.65	0.79	0.92	0.18	0.39	3.14	1.78	24.19	18.43	27.49	9.06	2.60
		1996	3.48	2.84	2.74	0.81	0.90	0.13	0.33	3.23	1.50	24.51	17.47	27.45	9.98	2.68
		1997	3.56	2.73	2.55	0.90	0.98	0.06	0.36	3.42	1.96	24.76	17.97	27.59	9.62	2.88
		1998	3.36	2.85	2.69	0.88	0.97	0.08	0.31	3.57	1.55	24.68	17.22	27.45	10.22	2.90
		1999	3.50	3.00	2.85	0.70	0.85	0.28	0.24	3.68	0.95	22.91	15.55	27.38	11.83	2.26
		2000	3.61	3.00	2.82	0.76	0.86	0.19	0.23	3.45	1.38	24.18	16.84	27.41	10.57	2.55
		2001	3.16	3.14	2.81	0.80	0.95	0.15	0.23	3.86	0.96	23.34	15.55	27.50	11.95	2.61
		2002	3.46	2.87	2.66	0.87	0.95	0.10	0.31	3.50	1.60	24.39	17.36	27.50	10.15	2.76
		2003	3.42	2.82	2.69	0.86	0.97	0.11	0.36	3.45	1.52	24.58	17.44	27.49	10.06	2.85
		2004	3.41	2.90	2.71	0.88	0.94	0.10	0.28	3.61	1.49	24.41	17.03	27.52	10.49	2.79
		2005	3.46	2.81	2.69	0.85	0.93	0.12	0.34	3.38	1.52	24.41	17.27	27.49	10.22	2.73
		2006	3.72	2.83	2.82	0.75	0.84	0.22	0.27	3.25	1.30	23.64	17.69	27.46	9.77	2.43
		2007	3.67	2.73	2.63	0.86	0.93	0.10	0.29	3.48	1.78	24.31	18.35	27.57	9.22	2.71
		2008	3.77	2.64	2.57	0.91	0.96	0.06	0.34	3.28	2.01	24.75	18.81	27.62	8.81	2.84
		2009	3.58	2.82	2.74	0.88	0.94	0.09	0.23	3.56	1.47	24.16	17.75	27.61	9.86	2.73
		2010	3.48	2.75	2.61	0.91	0.97	0.05	0.24	3.72	1.71	24.25	17.88	27.61	9.72	2.80
		2011	3.64	2.72	2.64	0.87	0.94	0.08	0.25	3.50	1.80	24.59	18.31	27.58	9.27	2.79
		2012	3.44	2.79	2.70	0.89	0.94	0.08	0.23	3.67	1.51	24.43	17.50	27.51	10.01	2.79
		2013	3.47	2.64	2.57	0.91	0.96	0.06	0.21	3.82	1.71	23.86	18.16	27.54	9.38	2.70
		2014	3.50	2.82	2.72	0.86	0.94	0.10	0.18	3.77	1.49	24.14	17.76	27.53	9.77	2.75
		2015	3.74	2.57	2.53	0.92	0.97	0.05	0.23	3.79	2.08	24.54	19.05	27.59	8.54	2.85
		2016	3.79	2.52	2.49	0.93	0.97	0.05	0.26	3.59	2.18	24.55	19.05	27.55	8.50	2.83
		2017	3.56	2.76	2.67	0.84	0.89	0.14	0.17	3.84	1.45	23.16	17.18	27.47	10.29	2.47
		2018	3.73	2.60	2.49	0.93	0.97	0.04	0.29	3.59	1.98	23.55	18.61	27.63	9.02	2.68
		2019	3.58	2.72	2.62	0.91	0.95	0.08	0.26	3.53	1.75	24.39	18.06	27.59	9.53	2.78
SP	AB	1982	3.42	2.85	2.68	0.83	0.91	0.16	0.25	3.77	1.46	23.95	17.01	27.44	10.42	2.64
		1983	3.45	2.96	2.77	0.80	0.89	0.17	0.23	3.80	1.38	24.30	16.61	27.46	10.85	2.70
		1984	3.93	2.57	2.65	0.73	0.91	0.26	0.47	3.07	1.85	24.31	18.91	27.51	8.60	2.63
		1985	3.38	2.71	2.74	0.73	0.95	0.26	0.46	3.31	1.19	23.48	18.21	27.36	9.14	2.64
		1986	3.35	2.81	2.74	0.81	0.92	0.18	0.30	3.73	1.27	24.14	17.24	27.36	10.11	2.75
		1987	3.09	2.93	2.86	0.79	0.88	0.20	0.25	3.79	0.89	24.06	15.49	27.35	11.86	2.59
		1988	3.19	2.83	2.75	0.84	0.92	0.13	0.26	3.69	1.20	24.17	16.81	27.37	10.56	2.70
		1989	3.43	2.91	2.82	0.80	0.85	0.19	0.32	3.28	1.25	24.52	16.52	27.37	10.85	2.60
		1990	3.73	2.64	2.56	0.86	0.93	0.12	0.46	3.05	1.96	24.71	18.21	27.53	9.32	2.74
		1991	3.54	2.78	2.66	0.85	0.91	0.15	0.39	3.32	1.62	24.67	16.92	27.45	10.53	2.73
		1992	3.34	2.69	2.64	0.86	0.94	0.13	0.42	3.29	1.48	24.25	17.43	27.48	10.05	2.68
		1993	3.07	2.96	2.79	0.82	0.88	0.18	0.26	3.74	0.99	23.74	15.55	27.43	11.88	2.54
		1994	3.31	2.96	2.85	0.78	0.82	0.21	0.26	3.47	1.07	23.94	15.99	27.34	11.35	2.47
		1995	3.63	3.08	2.95	0.68	0.75	0.30	0.29	3.16	1.11	24.28	16.02	27.33	11.31	2.40
		1996	3.32	2.97	2.86	0.81	0.85	0.18	0.27	3.47	1.12	24.55	16.33	27.34	11.02	2.64
		1997	3.18	2.93	2.75	0.86	0.94	0.12	0.33	3.57	1.23	24.36	16.55	27.46	10.91	2.80
		1998	3.22	2.87	2.71	0.90	0.96	0.07	0.35	3.46	1.37	24.68	16.89	27.45	10.56	2.87
		1999	3.07	3.12	2.94	0.78	0.86	0.19	0.22	3.71	0.78	24.11	15.12	27.33	12.20	2.60
		2000	3.29	3.14	2.99	0.75	0.83	0.22	0.23	3.51	0.86	24.40	15.54	27.33	11.79	2.60
		2001	2.96	3.18	2.92	0.83	0.91	0.14	0.23	3.85	0.79	24.33	14.96	27.38	12.43	2.79
		2002	3.38	3.01	2.87	0.81	0.88	0.17	0.30	3.41	1.20	24.67	16.38	27.38	11.00	2.73
		2003	3.25	3.03	2.87	0.82	0.91	0.14	0.29	3.44	1.11	24.60	16.51	27.42	10.91	2.79
		2004	3.00	2.96	2.81	0.87	0.93	0.11	0.23	3.79	0.97	24.03	16.01	27.44	11.43	2.70
		2005	3.14	3.00	2.86	0.81	0.88	0.16	0.23	3.61	0.94	23.98	15.86	27.47	11.61	2.58
		2006	3.48	2.98	2.91	0.76	0.84	0.22	0.27	3.28	1.10	24.18	16.83	27.45	10.62	2.54
		2007	3.34	2.77	2.74	0.89	0.93	0.09	0.32	3.36	1.36	24.49	17.50	27.50	10.00	2.75
		2008	3.56	2.95	2.85	0.81	0.86	0.17	0.27	3.33	1.31	24.45	17.08	27.49	10.41	2.64
		2009	3.42	2.97	2.78	0.87	0.91	0.11	0.29	3.43	1.37	24.71	16.85	27.54	10.69	2.78
		2010	2.88	3.10	2.75	0.91	0.95	0.07	0.19	4.03	0.99	23.68	15.35	27.54	12.19	2.72
		2011	3.47	2.83	2.77	0.86	0.91	0.12	0.24	3.57	1.39	24.56	17.35	27.50	10.15	2.75
		2012	3.48	2.84	2.74	0.85	0.92	0.11	0.27	3.49	1.49	24.59	17.57	27.52	9.95	2.76
		2013	3.83	2.70	2.62	0.90	0.93	0.08	0.34	3.23	1.99	25.04	18.54	27.62	9.07	2.84
		2014	3.67	2.74	2.67	0.91	0.97	0.07	0.22	3.79	1.79	24.78	18.21	27.62	9.40	2.92
		2015	3.67	2.74	2.64	0.89	0.96	0.10	0.27	3.70	1.79	24.61	18.29	27.59	9.30	2.88
		2016	3.68	2.87	2.85	0.74	0.80	0.23	0.21	3.42	1.36	24.09	17.29	27.40	10.11	2.43
		2017	3.34	2.94	2.83	0.78	0.86	0.19	0.17	3.72	1.10	23.60	16.84	27.46	10.62	2.49
		2018	3.30	2.79	2.73	0.88	0.91	0.10	0.17	3.83	1.30	23.91	17.17	27.53	10.35	2.61
		2019	3.35	2.92	2.77	0.83	0.87	0.15	0.17	3.82	1.28	23.97	16.48	27.44	10.95	2.58
SP	CC	1982	3.20	2.87	2.79	0.80	0.95	0.11	0.31	3.57	1.23	24.53	17.28	27.40	10.12	2.79
		1983	3.45	3.09	2.90	0.72	0.85	0.18	0.31	3.15	1.22	24.67	16.86	27.38	10.52	2.66
		1984	3.75	2.89	2.89	0.73	0.83	0.22	0.34	2.90	1.34	24.98	17.91	27.45	9.53	2.66
		1985	4.04	2.72	2.70	0.75	0.94	0.21	0.60	2.45	1.93	25.20	19.89	27.43	7.54	2.97
		1986	3.53	2.99	2.88	0.78	0.90	0.19	0.35	3.38	1.31	24.77	17.21	27.34	10.12	2.85
		1987	3.18	2.82	2.73	0.90	0.97	0.08	0.31	3.71	1.30	24.79	16.74	27.37	10.62	2.94
		1988	3.79	2.81	2.71	0.82	0.88	0.15	0.45	2.81	1.78	25.10	17.90	27.46	9.56	2.76
		1989	3.65	2.88	2.85	0.79	0.88	0.15	0.38	2.91	1.47	25.09	17.65	27.43	9.78	2.76
		1990	3.61	2.85	2.79	0.80	0.87	0.15	0.35	3.14	1.53	24.94	17.38	27.40	10.02	2.72
		1991	3.67	2.87	2.84	0.79	0.83	0.20	0.30	3.22	1.40	24.86	17.14	27.34	10.20	2.65
		1992	3.07	2.99	2.80	0.89	0.96	0.08	0.28	3.72	1.10	24.59	16.07	27.41	11.34	2.89
		1993	3.18	3.13	2.84	0.88	0.91	0.10	0.27	3.58	1.10	24.58	15.67	27.47	11.80	2.81
		1994	3.37	3.04	2.79	0.86	0.92	0.12	0.34	3.37	1.35	24.74	16.37	27.47	11.11	2.81
		1995	3.60	3.10	2.98	0.70	0.80	0.22	0.32	2.96	1.18	24.85	16.62	27.37	10.75	2.58
		1996	3.30	3.06	2.88	0.82	0.88	0.14	0.28	3.44	1.15	24.70	16.30	27.40	11.10	2.73
		1997	3.29	2.85	2.72	0.91	0.97	0.07	0.34	3.54	1.44	24.86	17.22	27.43	10.20	2.95
		1998	3.21	2.85	2.68	0.92	0.99	0.05	0.38	3.39	1.51	24.97	17.37	27.50	10.13	2.95
		1999	3.10	2.95	2.84	0.87	0.96	0.07	0.24	3.69	1.14	24.81	16.67	27.41	10.74	2.89
		2000	3.01	3.02	2.83	0.91	0.97	0.04	0.23	3.75	1.05	24.74	16.41	27.45	11.05	2.92
		2001	3.15	2.91	2.84	0.91	0.99	0.05	0.27	3.61	1.14	24.89	17.27	27.52	10.25	2.98
		2002	2.95	3.12	2.85	0.93	1.00	0.04	0.23	3.90	0.98	24.61	16.02	27.45	11.43	2.96
		2003	3.17	2.98	2.97	0.85	0.97	0.07	0.22	3.54	0.95	24.90	17.37	27.48	10.11	2.90
		2004	3.36	2.76	2.86	0.89	0.96	0.08	0.28	3.48	1.15	24.74	17.83	27.51	9.68	2.84
		2005	3.49	2.82	2.90	0.84	0.90	0.14	0.25	3.37	1.11	24.51	17.57	27.46	9.89	2.69
		2006	3.50	2.98	3.03	0.80	0.89	0.15	0.23	3.27	1.00	24.88	17.36	27.49	10.13	2.75
		2007	3.62	2.88	2.84	0.84	0.89	0.13	0.33	3.12	1.45	25.00	17.56	27.48	9.92	2.76
		2008	3.61	2.97	2.90	0.83	0.92	0.14	0.33	3.25	1.32	24.95	17.79	27.49	9.70	2.87
		2009	3.58	2.96	2.94	0.81	0.89	0.14	0.26	3.27	1.21	24.96	17.58	27.52	9.94	2.76
		2010	3.51	3.10	2.98	0.77	0.91	0.21	0.29	3.44	1.07	24.66	17.26	27.42	10.16	2.90
		2011	3.55	2.87	2.85	0.88	0.96	0.08	0.28	3.25	1.38	25.06	18.09	27.58	9.49	2.93
		2012	3.47	2.76	2.81	0.93	0.98	0.03	0.27	3.34	1.40	25.15	18.11	27.65	9.54	2.95
		2013	3.74	2.80	2.82	0.87	0.95	0.09	0.28	3.27	1.57	25.13	18.34	27.62	9.28	2.90
		2014	3.56	2.85	2.82	0.87	0.98	0.08	0.28	3.36	1.48	25.05	18.25	27.61	9.36	3.00
		2015	3.66	2.85	2.85	0.85	0.91	0.13	0.27	3.32	1.41	24.94	17.74	27.53	9.78	2.82
		2016	3.75	2.73	2.67	0.87	0.94	0.09	0.33	3.24	1.86	25.08	18.23	27.49	9.25	2.88
		2017	3.88	2.77	2.76	0.81	0.91	0.14	0.36	3.06	1.80	25.13	18.80	27.54	8.74	2.85
		2018	3.63	2.75	2.80	0.89	0.96	0.08	0.27	3.38	1.54	25.06	18.27	27.54	9.27	2.92
		2019	3.32	2.77	2.78	0.95	0.99	0.03	0.23	3.60	1.36	24.95	17.60	27.54	9.94	2.96
SP	UL	1982	3.10	3.20	2.95	0.68	0.97	0.15	0.44	3.62	0.85	24.62	16.56	27.25	10.70	2.98
		1983	3.38	2.89	2.69	0.83	0.98	0.09	0.47	3.14	1.57	24.93	17.38	27.47	10.09	2.91
		1984	3.66	2.63	2.65	0.73	0.96	0.22	0.62	2.83	1.72	24.92	17.61	27.66	10.05	2.76
		1985	3.48	2.83	2.67	0.86	0.97	0.09	0.47	3.12	1.63	24.96	17.64	27.56	9.92	2.89
		1986	2.96	3.01	2.73	0.88	0.98	0.05	0.34	3.70	1.18	24.71	16.23	27.40	11.17	2.95
		1987	2.77	3.11	2.79	0.91	0.99	0.06	0.28	3.96	0.86	24.44	15.12	27.44	12.32	2.94
		1988	3.14	2.84	2.67	0.90	0.99	0.05	0.41	3.46	1.36	24.84	16.85	27.47	10.62	2.94
		1989	2.86	3.05	2.77	0.92	1.00	0.05	0.30	3.82	1.05	24.60	15.40	27.52	12.12	2.95
		1990	3.04	3.01	2.78	0.84	1.00	0.13	0.40	3.62	1.05	24.53	15.34	27.66	12.31	2.87
		1991	2.58	3.38	2.89	0.93	0.98	0.05	0.16	4.24	0.49	24.19	13.87	27.47	13.61	2.94
		1992	2.46	3.37	2.91	0.90	0.99	0.05	0.17	4.35	0.50	24.16	13.63	27.50	13.87	2.93
		1993	2.62	3.36	2.94	0.91	0.96	0.08	0.18	4.20	0.52	24.20	13.81	27.51	13.70	2.88
		1994	2.58	3.44	2.95	0.92	0.96	0.07	0.16	4.27	0.47	24.14	13.45	27.49	14.04	2.89
		1995	2.76	3.41	2.88	0.91	0.95	0.08	0.22	4.04	0.73	24.21	13.81	27.51	13.70	2.87
		1996	2.63	3.36	2.87	0.93	0.98	0.06	0.20	4.16	0.66	24.23	13.89	27.55	13.66	2.93
		1997	2.60	3.28	2.85	0.88	1.00	0.10	0.26	4.21	0.63	24.12	13.53	27.53	14.00	2.90
		1998	2.51	3.44	2.92	0.92	0.97	0.06	0.16	4.33	0.50	24.09	13.40	27.50	14.10	2.91
		1999	2.58	3.47	2.94	0.90	0.95	0.08	0.16	4.29	0.50	24.09	13.28	27.47	14.19	2.87
		2000	2.80	3.34	2.96	0.82	0.91	0.16	0.22	4.02	0.58	24.19	13.74	27.45	13.71	2.75
		2001	2.58	3.41	2.92	0.93	0.98	0.06	0.18	4.26	0.53	24.16	13.66	27.49	13.83	2.93
		2002	2.49	3.42	2.90	0.93	0.99	0.04	0.16	4.33	0.52	24.16	13.62	27.52	13.89	2.97
		2003	2.57	3.50	2.96	0.87	0.95	0.11	0.17	4.30	0.44	24.03	13.11	27.49	14.38	2.87
		2004	2.60	3.45	2.92	0.91	0.97	0.05	0.19	4.24	0.56	24.18	13.68	27.50	13.82	2.92
		2005	2.65	3.38	2.91	0.94	0.98	0.04	0.18	4.14	0.62	24.29	14.08	27.54	13.46	2.94
		2006	2.73	3.19	2.89	0.90	1.00	0.09	0.25	4.06	0.66	24.34	14.38	27.57	13.19	2.91
		2007	2.59	3.36	2.91	0.90	0.99	0.08	0.21	4.23	0.56	24.17	13.58	27.55	13.97	2.91
		2008	2.89	3.26	2.92	0.88	0.97	0.07	0.27	3.88	0.73	24.49	14.98	27.52	12.54	2.93
		2009	2.93	3.31	2.95	0.89	0.94	0.09	0.21	3.82	0.72	24.45	14.68	27.57	12.89	2.85
		2010	2.60	3.38	2.91	0.93	1.00	0.06	0.19	4.22	0.56	24.20	13.72	27.61	13.89	2.94
		2011	2.78	3.41	3.00	0.84	0.92	0.12	0.19	4.04	0.52	24.22	13.88	27.43	13.55	2.82
		2012	2.79	3.29	2.97	0.87	0.96	0.09	0.20	3.94	0.60	24.42	14.52	27.55	13.03	2.87
		2013	2.67	3.37	2.93	0.94	0.99	0.03	0.16	4.10	0.60	24.34	14.32	27.58	13.27	2.96
		2014	2.56	3.39	3.01	0.94	1.00	0.04	0.12	4.28	0.34	24.27	13.72	27.60	13.88	2.96
		2015	2.36	3.49	2.99	0.93	1.00	0.04	0.09	4.49	0.26	24.07	13.10	27.51	14.41	2.96
		2016	2.56	3.42	3.01	0.90	0.98	0.07	0.12	4.28	0.35	24.21	13.65	27.54	13.89	2.92
		2017	2.52	3.62	2.99	0.92	0.95	0.07	0.11	4.38	0.37	23.97	12.82	27.54	14.72	2.88
		2018	2.46	3.54	2.94	0.95	0.99	0.03	0.13	4.39	0.43	24.06	13.22	27.55	14.34	2.96
		2019	2.53	3.54	3.04	0.87	0.99	0.10	0.16	4.32	0.26	24.05	13.49	27.56	14.07	2.97
SP	LL	1982	2.96	3.03	2.90	0.83	0.95	0.09	0.25	3.62	0.89	24.73	15.97	27.33	11.36	2.86
		1983	3.08	3.01	2.82	0.85	0.97	0.07	0.31	3.46	1.13	24.79	16.62	27.45	10.83	2.91
		1984	3.39	2.68	2.73	0.82	0.98	0.14	0.46	3.12	1.43	24.99	17.31	27.56	10.25	2.85
		1985	3.28	2.98	2.97	0.77	0.99	0.20	0.40	3.41	0.87	24.71	17.53	27.23	9.70	3.09
		1986	3.14	2.76	2.76	0.91	0.96	0.07	0.32	3.49	1.16	24.91	16.80	27.29	10.49	2.89
		1987	3.07	2.80	2.80	0.89	0.98	0.07	0.33	3.53	1.12	24.89	16.65	27.32	10.66	2.94
		1988	3.13	2.76	2.77	0.91	0.97	0.07	0.33	3.49	1.19	24.95	16.82	27.33	10.51	2.91
		1989	3.22	2.74	2.73	0.92	0.99	0.03	0.37	3.29	1.35	25.09	17.46	27.44	9.98	2.95
		1990	3.06	2.76	2.83	0.88	1.00	0.09	0.33	3.55	1.03	24.90	16.48	27.48	11.00	2.92
		1991	3.06	2.81	2.70	0.95	1.00	0.02	0.34	3.57	1.28	24.89	16.63	27.37	10.74	2.98
		1992	2.79	3.03	2.79	0.94	1.00	0.03	0.26	3.90	0.94	24.56	15.47	27.40	11.93	2.96
		1993	2.89	3.01	2.77	0.95	0.99	0.03	0.27	3.81	1.04	24.63	15.57	27.41	11.83	2.95
		1994	2.77	3.11	2.82	0.95	0.99	0.03	0.24	3.97	0.87	24.51	15.10	27.43	12.34	2.95
		1995	3.13	2.96	2.70	0.94	1.00	0.02	0.37	3.46	1.35	24.84	16.57	27.49	10.92	2.99
		1996	2.88	3.10	2.75	0.96	1.00	0.02	0.29	3.79	1.09	24.61	15.38	27.47	12.09	2.97
		1997	2.85	2.95	2.81	0.94	1.00	0.02	0.26	3.82	0.95	24.74	16.06	27.38	11.32	2.99
		1998	3.02	2.88	2.79	0.90	0.97	0.05	0.29	3.61	1.10	24.83	16.55	27.39	10.84	2.92
		1999	2.80	2.99	2.83	0.92	0.98	0.03	0.25	3.87	0.89	24.68	15.73	27.30	11.57	2.95
		2000	2.91	2.93	2.77	0.93	0.99	0.03	0.29	3.74	1.08	24.77	16.18	27.36	11.17	2.97
		2001	2.90	3.03	2.82	0.95	0.99	0.03	0.25	3.83	0.96	24.69	15.65	27.39	11.75	2.97
		2002	3.15	2.83	2.81	0.90	1.00	0.04	0.33	3.41	1.17	25.04	17.39	27.43	10.04	3.01
		2003	2.90	2.84	2.85	0.91	1.00	0.03	0.27	3.76	0.87	24.87	16.58	27.40	10.82	2.96
		2004	2.86	2.94	2.96	0.86	1.00	0.06	0.25	3.83	0.66	24.73	16.57	27.45	10.88	2.91
		2005	3.29	2.83	2.94	0.83	0.95	0.07	0.28	3.22	1.03	25.16	17.74	27.47	9.73	2.88
		2006	3.44	2.86	2.85	0.84	1.00	0.14	0.43	3.17	1.22	25.12	18.11	27.63	9.52	2.86
		2007	3.25	2.76	2.82	0.91	1.00	0.05	0.35	3.35	1.23	25.10	17.74	27.51	9.77	2.95
		2008	3.05	2.91	2.84	0.92	0.97	0.05	0.26	3.62	1.00	24.86	16.45	27.42	10.97	2.92
		2009	3.49	2.71	2.73	0.93	0.97	0.05	0.39	3.03	1.53	25.24	17.93	27.55	9.62	2.91
		2010	3.59	2.57	2.57	0.95	1.00	0.01	0.49	2.83	1.90	25.15	18.96	27.59	8.63	2.93
		2011	2.93	2.89	2.83	0.89	1.00	0.06	0.27	3.73	0.93	24.65	16.32	27.41	11.09	2.92
		2012	3.14	2.90	2.77	0.93	1.00	0.01	0.32	3.49	1.24	24.93	16.88	27.50	10.62	2.98
		2013	3.44	2.75	2.68	0.93	0.99	0.02	0.40	3.10	1.65	25.16	17.85	27.52	9.68	2.95
		2014	3.47	2.81	2.71	0.90	0.99	0.02	0.38	2.99	1.66	25.20	18.25	27.60	9.35	2.96
		2015	2.97	2.92	2.80	0.94	0.99	0.02	0.26	3.69	1.06	24.77	16.36	27.46	11.10	2.95
		2016	2.92	3.03	2.88	0.89	0.99	0.03	0.25	3.72	0.91	24.78	16.11	27.43	11.32	2.96
		2017	3.16	2.98	2.80	0.91	0.97	0.05	0.30	3.53	1.20	24.84	16.45	27.50	11.06	2.92
		2018	3.36	2.86	2.69	0.93	0.99	0.04	0.38	3.23	1.56	25.03	17.30	27.56	10.26	2.95
		2019	3.38	2.79	2.76	0.88	0.94	0.08	0.36	3.18	1.43	25.07	17.50	27.46	9.96	2.86
FA	SL	1986	2.99	3.26	3.02	0.69	0.81	0.27	0.16	3.96	0.36	23.37	15.48	27.12	11.64	2.48
		1987	2.71	3.12	2.92	0.71	0.83	0.24	0.17	4.05	0.25	22.61	15.36	27.10	11.75	2.36
		1988	3.40	3.35	3.20	0.55	0.69	0.41	0.22	3.47	0.39	23.56	15.34	27.14	11.79	2.26
		1989	2.92	3.32	3.05	0.69	0.80	0.29	0.16	3.88	0.27	22.94	14.92	27.21	12.29	2.39
		1990	3.21	3.16	2.95	0.69	0.78	0.28	0.23	3.57	0.65	23.22	15.85	27.24	11.39	2.35
		1991	3.04	3.16	3.08	0.72	0.86	0.27	0.19	3.91	0.25	22.74	16.17	27.17	11.00	2.46
		1992	3.18	3.41	3.19	0.62	0.64	0.37	0.08	3.70	0.21	22.99	13.86	27.16	13.29	2.04
		1993	3.25	3.15	3.15	0.48	0.86	0.49	0.42	3.65	0.22	23.20	15.35	27.28	11.93	2.50
		1994	3.32	3.42	3.21	0.57	0.70	0.37	0.16	3.69	0.24	23.35	14.53	27.15	12.62	2.26
		1995	2.99	3.32	3.05	0.67	0.81	0.28	0.20	3.88	0.33	23.12	14.39	27.28	12.89	2.39
		1996	3.14	3.45	3.27	0.58	0.67	0.39	0.10	3.78	0.07	23.42	13.79	27.14	13.35	2.19
		1997	2.65	3.45	3.10	0.72	0.81	0.24	0.10	4.30	0.09	23.28	13.34	27.26	13.92	2.45
		1998	2.45	3.07	2.87	0.77	0.82	0.21	0.07	4.26	0.15	21.49	13.75	27.20	13.45	2.06
		1999	3.11	3.48	3.20	0.61	0.73	0.34	0.16	3.81	0.22	23.74	14.29	27.15	12.86	2.40
		2000	2.84	3.54	3.15	0.65	0.79	0.28	0.16	4.07	0.14	23.43	13.77	27.22	13.44	2.47
		2001	3.36	3.56	3.31	0.54	0.89	0.41	0.39	3.57	0.22	23.78	16.95	27.13	10.18	2.98
		2002	2.88	3.31	3.09	0.72	0.83	0.25	0.17	3.97	0.31	23.45	15.03	27.23	12.20	2.54
		2003	3.19	3.36	3.14	0.63	0.83	0.34	0.24	3.85	0.39	23.52	15.34	27.24	11.90	2.57
		2004	2.98	3.37	3.08	0.68	0.80	0.29	0.17	3.96	0.36	23.65	14.31	27.27	12.96	2.48
		2005	2.68	3.18	2.92	0.74	0.86	0.20	0.16	4.24	0.37	22.77	14.49	27.28	12.79	2.38
		2006	3.53	3.57	3.30	0.53	0.91	0.43	0.42	3.48	0.35	23.85	17.52	27.16	9.63	3.03
		2007	3.43	3.44	3.28	0.56	0.91	0.42	0.40	3.53	0.32	23.84	17.50	27.15	9.65	3.02
		2008	2.62	3.14	2.96	0.75	0.90	0.23	0.18	4.24	0.20	22.54	15.36	27.23	11.87	2.53
		2009	3.23	3.41	3.18	0.59	0.88	0.35	0.33	3.78	0.36	23.80	16.32	27.17	10.86	2.83
		2010	2.80	3.35	3.03	0.76	0.88	0.22	0.15	4.22	0.36	23.56	14.79	27.30	12.51	2.68
		2011	2.81	3.23	2.98	0.68	0.86	0.25	0.21	4.06	0.32	22.75	15.74	27.20	11.46	2.49
		2012	3.40	3.48	3.24	0.55	0.81	0.41	0.31	3.52	0.37	23.75	16.44	27.17	10.73	2.68
		2013	3.03	3.32	3.02	0.66	0.84	0.25	0.24	3.93	0.57	23.73	15.23	27.27	12.04	2.58
		2014	2.99	3.24	3.05	0.68	0.88	0.28	0.25	4.04	0.43	23.74	15.49	27.28	11.79	2.69
		2015	3.04	3.27	3.06	0.68	0.87	0.27	0.25	3.95	0.48	23.77	16.11	27.22	11.10	2.71
		2016	2.85	3.20	2.93	0.78	0.92	0.20	0.20	4.09	0.55	23.26	16.14	27.31	11.17	2.70
		2017	2.62	3.00	2.83	0.74	0.90	0.21	0.21	4.13	0.36	21.64	16.22	27.23	11.01	2.29
		2018	2.96	3.09	2.90	0.73	0.88	0.25	0.20	4.02	0.64	22.92	15.76	27.39	11.62	2.46
		2019	3.10	3.06	2.94	0.71	0.80	0.27	0.12	4.03	0.57	22.42	16.06	27.25	11.19	2.25
FA	GB	1982	3.65	3.08	2.91	0.67	0.85	0.27	0.45	3.06	1.28	24.71	17.58	27.30	9.72	2.75
		1983	4.04	3.31	3.24	0.47	0.82	0.50	0.53	2.78	0.82	24.34	18.44	27.14	8.70	2.85
		1984	3.77	3.08	3.02	0.60	0.84	0.37	0.49	2.96	1.09	24.54	17.60	27.28	9.68	2.75
		1985	3.62	3.35	3.13	0.59	0.88	0.36	0.46	3.20	0.84	24.46	17.64	27.24	9.59	2.95
		1986	3.68	3.36	3.17	0.58	0.90	0.38	0.48	3.15	0.77	24.43	17.85	27.21	9.36	3.01
		1987	3.80	3.45	3.28	0.48	0.86	0.48	0.50	3.08	0.64	24.39	18.02	27.16	9.14	2.99
		1988	3.58	3.22	3.06	0.64	0.84	0.33	0.40	3.23	0.94	24.53	17.08	27.23	10.15	2.79
		1989	3.28	3.46	3.09	0.68	0.86	0.28	0.31	3.55	0.66	24.25	15.64	27.32	11.68	2.80
		1990	3.92	3.24	3.13	0.53	0.87	0.45	0.53	2.93	0.98	24.46	18.27	27.24	8.97	2.93
		1991	4.03	3.31	3.21	0.50	0.80	0.48	0.50	2.75	0.85	24.39	18.04	27.19	9.15	2.80
		1992	3.79	3.27	3.14	0.54	0.83	0.43	0.47	3.06	0.86	24.32	17.60	27.20	9.60	2.79
		1993	3.40	3.28	3.08	0.59	0.91	0.37	0.44	3.48	0.76	24.18	16.49	27.37	10.88	2.83
		1994	3.78	3.10	3.02	0.58	0.79	0.39	0.35	3.24	0.88	23.51	16.45	27.30	10.84	2.38
		1995	3.89	3.52	3.38	0.40	0.87	0.53	0.55	3.00	0.47	24.33	18.13	27.12	8.99	3.06
		1996	3.84	3.47	3.34	0.45	0.86	0.50	0.50	3.06	0.54	24.38	17.94	27.12	9.18	2.98
		1997	3.59	3.37	3.23	0.52	0.89	0.42	0.44	3.40	0.57	24.32	17.40	27.18	9.78	2.99
		1998	3.55	3.40	3.22	0.53	0.87	0.38	0.41	3.41	0.52	24.18	17.03	27.10	10.07	2.89
		1999	3.60	3.36	3.18	0.56	0.91	0.39	0.44	3.40	0.63	24.33	17.46	27.20	9.74	3.03
		2000	3.18	3.44	3.14	0.59	0.94	0.30	0.42	3.72	0.53	24.33	16.76	27.21	10.44	3.04
		2001	3.78	3.41	3.25	0.53	0.94	0.44	0.54	3.13	0.69	24.37	18.62	27.20	8.58	3.19
		2002	3.67	3.38	3.25	0.56	0.91	0.43	0.43	3.40	0.60	24.24	17.95	27.20	9.25	3.06
		2003	4.06	3.43	3.34	0.43	0.87	0.55	0.53	2.99	0.67	24.26	18.70	27.15	8.45	3.04
		2004	3.97	3.37	3.25	0.48	0.90	0.48	0.50	3.15	0.81	24.24	18.62	27.20	8.58	3.02
		2005	3.71	3.31	3.22	0.49	0.92	0.44	0.53	3.26	0.69	24.39	18.08	27.21	9.13	3.04
		2006	3.42	3.30	3.07	0.66	0.91	0.29	0.38	3.54	0.91	24.42	17.26	27.32	10.06	2.93
		2007	3.87	3.27	3.14	0.58	0.90	0.38	0.44	3.28	0.99	24.34	18.63	27.26	8.62	3.00
		2008	3.63	3.30	3.19	0.56	0.90	0.40	0.43	3.40	0.69	24.13	18.02	27.21	9.19	2.96
		2009	3.45	3.22	2.99	0.68	0.89	0.26	0.33	3.64	1.03	24.25	17.08	27.31	10.23	2.81
		2010	3.97	3.23	3.13	0.55	0.91	0.41	0.48	3.16	1.07	24.45	19.12	27.25	8.13	3.04
		2011	3.47	3.21	3.00	0.68	0.87	0.26	0.31	3.53	1.05	24.48	17.30	27.32	10.02	2.79
		2012	3.32	3.31	3.00	0.57	0.96	0.20	0.48	3.69	1.01	24.56	17.46	27.28	9.82	3.02
		2013	3.57	3.21	2.98	0.67	0.87	0.27	0.34	3.50	1.26	24.54	17.12	27.40	10.28	2.77
		2014	3.41	3.28	3.09	0.66	0.90	0.29	0.31	3.67	0.86	24.41	17.27	27.32	10.05	2.90
		2015	3.64	3.30	3.11	0.58	0.91	0.33	0.42	3.43	0.94	24.37	18.07	27.27	9.20	2.97
		2016	3.33	3.13	2.93	0.73	0.85	0.22	0.23	3.71	1.07	23.94	16.80	27.40	10.61	2.57
		2017	3.64	3.18	3.07	0.64	0.80	0.33	0.26	3.41	0.91	23.82	17.37	27.34	9.97	2.51
		2018	3.56	3.10	2.98	0.71	0.84	0.27	0.20	3.62	1.01	23.64	17.12	27.40	10.28	2.52
		2019	3.93	3.22	3.12	0.55	0.69	0.42	0.20	3.36	1.01	23.93	16.67	27.27	10.60	2.30
FA	MG	1982	3.14	3.20	3.10	0.64	0.78	0.32	0.19	3.72	0.42	23.97	14.87	27.30	12.43	2.39
		1983	3.35	3.29	3.11	0.62	0.72	0.34	0.17	3.43	0.58	23.98	15.09	27.25	12.16	2.28
		1984	3.98	3.12	3.10	0.52	0.72	0.46	0.38	2.86	1.07	24.24	17.06	27.30	10.24	2.36
		1985	3.28	2.98	2.87	0.73	0.88	0.23	0.31	3.49	1.07	24.54	16.70	27.36	10.66	2.67
		1986	3.46	3.19	3.06	0.67	0.85	0.30	0.29	3.38	0.83	24.41	16.45	27.33	10.88	2.70
		1987	3.29	3.09	3.00	0.67	0.83	0.28	0.30	3.52	0.80	24.41	15.99	27.29	11.30	2.60
		1988	3.48	3.06	2.90	0.69	0.86	0.25	0.36	3.35	1.19	24.58	16.78	27.42	10.64	2.67
		1989	3.61	3.04	2.80	0.75	0.88	0.21	0.38	3.22	1.48	24.69	17.08	27.46	10.38	2.73
		1990	3.85	3.00	2.91	0.68	0.90	0.30	0.49	2.96	1.40	24.76	18.42	27.41	8.99	2.88
		1991	3.59	2.99	2.87	0.75	0.80	0.24	0.33	3.09	1.29	24.45	16.80	27.35	10.55	2.51
		1992	3.63	3.05	2.98	0.65	0.78	0.31	0.33	3.09	1.06	24.43	16.41	27.33	10.92	2.46
		1993	3.53	3.04	2.96	0.66	0.81	0.31	0.35	3.24	1.04	24.32	16.34	27.39	11.05	2.52
		1994	3.53	3.09	3.02	0.58	0.77	0.36	0.33	3.31	0.90	24.28	15.67	27.35	11.69	2.39
		1995	3.87	3.09	3.02	0.58	0.72	0.37	0.36	2.92	1.15	24.48	16.48	27.31	10.84	2.36
		1996	3.46	3.16	2.99	0.66	0.79	0.30	0.30	3.40	0.97	24.31	15.69	27.37	11.69	2.48
		1997	3.31	3.05	3.16	0.46	0.78	0.50	0.33	3.65	0.32	23.66	14.23	27.39	13.17	2.24
		1998	3.64	3.28	3.22	0.50	0.63	0.47	0.19	3.40	0.42	23.48	14.18	27.24	13.06	2.04
		1999	3.67	3.11	2.98	0.63	0.80	0.32	0.29	3.25	1.13	24.54	16.81	27.37	10.55	2.53
		2000	3.15	3.18	2.93	0.76	0.91	0.18	0.28	3.73	0.99	24.53	16.06	27.42	11.35	2.77
		2001	3.47	2.95	2.85	0.76	0.92	0.20	0.34	3.47	1.23	24.31	17.08	27.49	10.41	2.73
		2002	3.25	3.15	3.04	0.66	0.83	0.28	0.20	3.72	0.68	23.96	15.70	27.37	11.67	2.50
		2003	3.46	3.13	3.01	0.69	0.85	0.28	0.26	3.61	0.94	24.20	16.35	27.37	11.02	2.65
		2004	3.63	3.10	3.09	0.61	0.81	0.37	0.31	3.33	0.82	24.30	16.68	27.33	10.65	2.56
		2005	3.69	2.98	2.94	0.69	0.81	0.28	0.23	3.47	1.25	24.42	17.04	27.48	10.44	2.53
		2006	3.77	3.13	3.02	0.64	0.74	0.32	0.19	3.31	1.16	24.29	16.72	27.41	10.69	2.37
		2007	3.89	2.94	3.02	0.71	0.82	0.28	0.26	3.12	1.17	24.52	17.88	27.48	9.60	2.55
		2008	3.68	3.13	2.93	0.69	0.80	0.27	0.24	3.47	1.28	24.48	16.65	27.44	10.79	2.56
		2009	3.87	3.19	3.07	0.59	0.76	0.35	0.27	3.30	1.15	24.48	16.94	27.38	10.44	2.49
		2010	3.58	2.95	2.87	0.75	0.91	0.22	0.28	3.67	1.21	23.87	17.51	27.43	9.92	2.71
		2011	3.84	3.16	3.05	0.64	0.75	0.30	0.20	3.32	1.18	24.67	17.16	27.38	10.22	2.52
		2012	3.44	2.91	2.85	0.83	0.92	0.14	0.21	3.52	1.33	24.80	17.48	27.58	10.10	2.80
		2013	3.68	3.04	2.84	0.78	0.88	0.19	0.23	3.45	1.61	24.78	17.33	27.63	10.30	2.69
		2014	3.60	2.94	2.83	0.79	0.96	0.16	0.27	3.61	1.48	24.77	17.84	27.59	9.75	2.89
		2015	3.67	3.20	3.10	0.63	0.74	0.34	0.18	3.36	0.88	23.97	16.54	27.34	10.80	2.37
		2016	3.84	3.00	2.99	0.66	0.77	0.31	0.24	3.21	1.25	24.58	17.41	27.41	10.00	2.46
		2017	3.53	3.06	2.97	0.67	0.75	0.30	0.14	3.60	0.99	23.44	16.70	27.35	10.65	2.27
		2018	3.39	2.91	2.81	0.76	0.87	0.21	0.17	3.74	1.22	23.63	16.91	27.55	10.63	2.47
		2019	3.41	2.90	2.88	0.78	0.88	0.19	0.18	3.58	1.18	24.22	17.26	27.52	10.26	2.58
FA	SA	1982	3.37	3.08	2.89	0.72	0.85	0.24	0.27	3.62	1.04	24.33	16.07	27.38	11.31	2.62
		1983	3.23	3.12	2.92	0.70	0.88	0.22	0.27	3.78	0.88	24.06	16.19	27.33	11.14	2.64
		1984	3.54	2.75	2.73	0.72	0.99	0.24	0.51	3.38	1.46	24.58	18.10	27.48	9.38	2.90
		1985	3.10	2.72	2.73	0.78	0.97	0.18	0.39	3.72	1.03	24.06	16.93	27.44	10.51	2.73
		1986	3.25	2.77	2.71	0.81	0.96	0.14	0.34	3.73	1.25	24.18	17.12	27.41	10.29	2.79
		1987	3.18	2.58	2.69	0.67	0.97	0.31	0.41	3.76	0.85	22.79	16.07	27.51	11.44	2.33
		1988	3.30	2.91	2.86	0.75	0.95	0.23	0.36	3.57	1.01	24.20	17.06	27.43	10.37	2.76
		1989	3.15	2.91	2.73	0.83	0.94	0.11	0.32	3.60	1.19	24.23	16.74	27.46	10.72	2.74
		1990	3.32	2.72	2.69	0.79	0.96	0.19	0.41	3.49	1.34	24.46	16.77	27.56	10.79	2.74
		1991	3.09	2.84	2.72	0.82	0.97	0.15	0.39	3.64	1.15	24.29	16.20	27.52	11.32	2.77
		1992	3.31	2.83	2.77	0.76	0.93	0.21	0.37	3.51	1.18	24.08	17.15	27.44	10.29	2.64
		1993	3.36	2.84	2.73	0.77	0.91	0.20	0.33	3.57	1.28	23.95	16.60	27.50	10.90	2.57
		1994	3.48	2.94	2.79	0.76	0.87	0.19	0.32	3.32	1.29	24.12	16.81	27.42	10.61	2.55
		1995	3.54	2.93	2.78	0.75	0.93	0.16	0.43	3.14	1.54	24.83	17.94	27.44	9.50	2.85
		1996	3.27	3.11	2.84	0.75	0.92	0.17	0.31	3.60	1.10	24.04	16.24	27.46	11.22	2.63
		1997	3.21	2.88	2.76	0.80	0.97	0.13	0.36	3.61	1.23	24.22	16.79	27.47	10.68	2.77
		1998	3.23	3.14	2.90	0.76	0.91	0.20	0.23	3.86	0.82	23.45	15.79	27.43	11.65	2.55
		1999	3.31	3.21	2.95	0.68	0.89	0.27	0.18	3.90	0.73	23.42	15.84	27.42	11.58	2.41
		2000	2.93	3.31	2.96	0.76	0.90	0.16	0.22	3.90	0.65	23.97	15.12	27.42	12.31	2.70
		2001	3.06	3.14	2.85	0.84	0.99	0.12	0.23	4.04	0.91	23.88	16.05	27.51	11.46	2.83
		2002	3.19	3.11	2.98	0.72	0.95	0.23	0.30	3.77	0.84	24.27	16.69	27.46	10.77	2.84
		2003	3.20	3.09	2.93	0.73	0.96	0.21	0.27	3.85	0.86	23.99	16.54	27.47	10.93	2.77
		2004	3.49	2.94	2.99	0.65	0.88	0.32	0.28	3.65	0.78	23.76	16.59	27.42	10.83	2.54
		2005	3.51	2.86	2.86	0.72	0.92	0.23	0.27	3.66	0.99	23.43	17.17	27.51	10.34	2.53
		2006	3.49	2.94	2.83	0.80	0.92	0.15	0.25	3.65	1.24	24.02	17.21	27.54	10.33	2.68
		2007	3.79	2.84	2.87	0.75	0.87	0.22	0.21	3.53	1.22	23.58	17.56	27.54	9.98	2.46
		2008	3.36	3.10	2.87	0.81	0.91	0.14	0.22	3.69	1.17	24.45	16.61	27.55	10.94	2.75
		2009	2.98	3.12	2.83	0.85	0.97	0.11	0.22	4.04	0.86	23.99	15.43	27.57	12.13	2.76
		2010	3.25	3.00	2.87	0.80	0.96	0.16	0.24	3.85	0.98	23.86	16.70	27.55	10.85	2.73
		2011	3.00	3.06	2.92	0.84	0.93	0.12	0.19	3.94	0.82	24.51	16.11	27.48	11.37	2.81
		2012	3.26	2.90	2.81	0.84	0.97	0.10	0.21	3.72	1.19	24.61	17.31	27.58	10.27	2.86
		2013	3.62	2.97	2.83	0.82	0.94	0.15	0.25	3.58	1.38	24.45	17.48	27.61	10.12	2.82
		2014	3.53	2.77	2.82	0.78	0.97	0.18	0.28	3.61	1.33	24.70	17.45	27.62	10.17	2.83
		2015	3.53	2.69	2.75	0.83	0.94	0.13	0.21	3.65	1.33	24.01	17.94	27.60	9.66	2.63
		2016	3.93	3.07	3.09	0.60	0.70	0.38	0.18	3.28	0.93	23.78	16.48	27.35	10.87	2.22
		2017	3.24	3.09	2.90	0.78	0.89	0.16	0.16	3.90	0.85	23.47	15.96	27.46	11.50	2.55
		2018	3.41	2.89	2.73	0.86	0.95	0.12	0.23	3.81	1.30	23.66	16.75	27.59	10.84	2.70
		2019	3.56	2.82	2.76	0.81	0.91	0.16	0.25	3.69	1.19	23.08	17.16	27.49	10.33	2.49
FA	AB	1982	2.90	3.15	3.00	0.64	0.88	0.30	0.27	3.91	0.46	23.81	14.48	27.46	12.99	2.52
		1983	3.10	3.13	2.99	0.63	0.87	0.31	0.30	3.69	0.65	23.75	15.46	27.42	11.95	2.52
		1984	3.52	2.85	2.92	0.57	0.92	0.41	0.51	3.29	0.91	23.78	17.66	27.36	9.71	2.66
		1985	2.87	2.94	2.79	0.81	0.97	0.14	0.31	3.96	0.87	24.00	16.40	27.43	11.03	2.79
		1986	3.16	3.12	2.97	0.72	0.88	0.22	0.29	3.67	0.82	24.31	16.14	27.37	11.23	2.70
		1987	2.77	3.00	2.94	0.68	0.95	0.25	0.31	4.03	0.50	23.99	14.88	27.45	12.57	2.65
		1988	3.08	3.14	3.01	0.67	0.82	0.28	0.24	3.78	0.60	24.06	14.99	27.36	12.37	2.49
		1989	2.89	3.17	2.90	0.77	0.90	0.18	0.26	3.90	0.68	24.06	14.80	27.41	12.61	2.67
		1990	2.84	2.94	2.88	0.73	0.95	0.24	0.35	3.98	0.61	23.98	15.01	27.46	12.46	2.69
		1991	3.30	2.84	2.74	0.79	0.91	0.18	0.42	3.38	1.29	24.53	16.57	27.46	10.89	2.69
		1992	2.94	2.90	2.80	0.77	0.92	0.20	0.33	3.78	0.86	23.77	15.62	27.44	11.82	2.57
		1993	2.63	3.03	2.90	0.76	0.93	0.22	0.24	4.19	0.43	23.54	14.22	27.45	13.22	2.58
		1994	3.20	2.98	2.90	0.70	0.86	0.26	0.34	3.58	0.91	24.23	15.51	27.41	11.90	2.56
		1995	3.27	3.10	2.93	0.70	0.85	0.24	0.33	3.49	0.98	24.37	15.68	27.41	11.74	2.59
		1996	3.13	3.19	2.97	0.74	0.83	0.22	0.26	3.64	0.79	24.28	15.04	27.35	12.31	2.58
		1997	2.73	3.04	2.92	0.75	0.95	0.19	0.29	4.10	0.55	23.98	14.90	27.43	12.54	2.72
		1998	3.06	3.14	3.05	0.64	0.85	0.30	0.27	3.73	0.52	23.98	14.84	27.39	12.55	2.51
		1999	2.72	3.17	2.96	0.77	0.93	0.18	0.22	4.08	0.47	24.01	14.65	27.42	12.77	2.73
		2000	2.99	3.38	3.08	0.70	0.85	0.22	0.21	3.74	0.52	24.25	14.70	27.40	12.70	2.64
		2001	3.16	3.11	2.98	0.70	0.94	0.25	0.34	3.72	0.79	24.32	15.88	27.44	11.55	2.81
		2002	3.04	3.17	2.99	0.73	0.87	0.22	0.23	3.85	0.67	24.15	15.31	27.38	12.07	2.63
		2003	2.99	3.01	2.93	0.74	0.92	0.21	0.28	3.87	0.70	23.98	15.56	27.46	11.90	2.65
		2004	3.07	2.97	2.91	0.76	0.91	0.21	0.27	3.75	0.78	23.97	16.16	27.42	11.26	2.64
		2005	3.26	2.94	2.87	0.76	0.90	0.20	0.30	3.63	1.05	24.25	16.59	27.49	10.90	2.64
		2006	3.36	3.10	3.00	0.72	0.81	0.25	0.24	3.42	0.88	24.10	16.42	27.41	10.99	2.49
		2007	3.27	2.87	2.87	0.76	0.90	0.21	0.31	3.53	1.05	24.29	16.56	27.52	10.96	2.62
		2008	2.91	3.31	2.92	0.83	0.92	0.11	0.17	4.06	0.78	24.07	15.16	27.52	12.37	2.74
		2009	3.25	3.22	3.01	0.70	0.83	0.24	0.23	3.66	0.78	24.19	15.23	27.48	12.24	2.55
		2010	3.18	3.10	2.99	0.70	0.95	0.27	0.32	3.82	0.73	24.00	16.41	27.48	11.07	2.81
		2011	3.16	3.06	2.89	0.78	0.93	0.12	0.21	3.67	1.02	24.50	16.75	27.54	10.78	2.77
		2012	3.07	3.17	2.91	0.82	0.93	0.10	0.20	3.72	0.91	24.45	16.17	27.53	11.36	2.81
		2013	3.44	3.08	2.87	0.81	0.93	0.12	0.24	3.58	1.32	24.67	17.06	27.62	10.56	2.80
		2014	3.14	3.02	2.82	0.84	0.98	0.12	0.24	3.88	1.12	24.51	16.27	27.60	11.33	2.90
		2015	3.02	2.98	2.93	0.77	0.91	0.20	0.21	3.91	0.69	23.91	16.15	27.45	11.30	2.65
		2016	3.35	2.94	2.96	0.65	0.79	0.33	0.24	3.56	0.76	23.38	16.16	27.41	11.25	2.28
		2017	3.33	2.94	2.89	0.72	0.82	0.25	0.20	3.69	0.97	23.58	16.30	27.47	11.17	2.38
		2018	3.40	2.95	2.83	0.77	0.84	0.21	0.22	3.60	1.13	23.67	16.49	27.51	11.03	2.43
		2019	3.35	3.03	2.88	0.76	0.87	0.19	0.23	3.60	1.15	24.36	16.53	27.48	10.95	2.65
FA	CC	1982	3.14	3.46	3.13	0.58	0.93	0.30	0.39	3.76	0.47	24.27	16.75	27.29	10.55	2.95
		1983	3.40	3.49	3.21	0.57	0.74	0.36	0.23	3.57	0.52	24.27	15.26	27.25	11.99	2.45
		1984	3.20	3.27	3.10	0.56	0.87	0.33	0.39	3.70	0.47	24.30	16.38	27.22	10.84	2.80
		1985	3.15	3.19	2.99	0.70	0.96	0.23	0.38	3.79	0.79	24.47	16.94	27.31	10.37	2.99
		1986	3.09	3.51	3.13	0.63	0.94	0.26	0.36	3.74	0.58	24.40	16.66	27.30	10.64	3.00
		1987	2.87	3.38	3.02	0.76	0.95	0.16	0.28	4.06	0.71	24.38	15.97	27.37	11.40	2.82
		1988	3.05	3.28	2.95	0.70	0.89	0.18	0.31	3.82	0.94	24.47	15.58	27.39	11.81	2.75
		1989	3.11	3.44	3.13	0.64	0.79	0.25	0.22	3.73	0.50	24.25	14.57	27.30	12.73	2.54
		1990	3.26	3.17	3.03	0.74	0.84	0.23	0.20	3.64	0.73	24.49	15.85	27.33	11.48	2.67
		1991	3.02	3.23	3.01	0.73	0.86	0.21	0.24	3.88	0.61	24.35	15.31	27.28	11.97	2.70
		1992	2.83	3.26	2.98	0.75	0.92	0.18	0.26	3.95	0.64	24.34	15.31	27.37	12.06	2.78
		1993	2.82	3.26	2.97	0.80	0.95	0.16	0.26	4.03	0.61	24.29	15.18	27.41	12.23	2.83
		1994	2.86	3.32	2.95	0.78	0.93	0.15	0.24	3.93	0.78	24.37	15.51	27.40	11.89	2.79
		1995	3.11	3.25	2.98	0.77	0.90	0.18	0.27	3.66	0.82	24.51	15.64	27.47	11.83	2.75
		1996	3.02	3.24	2.92	0.75	0.94	0.16	0.31	3.83	0.87	24.43	15.73	27.45	11.72	2.83
		1997	2.81	3.19	2.97	0.73	0.96	0.19	0.31	4.00	0.57	24.34	15.58	27.37	11.79	2.87
		1998	3.12	3.32	3.05	0.65	0.90	0.23	0.33	3.83	0.65	24.36	15.97	27.35	11.38	2.69
		1999	3.28	3.38	3.11	0.61	0.94	0.27	0.39	3.72	0.69	24.46	17.04	27.28	10.24	3.01
		2000	2.60	3.33	2.95	0.82	0.99	0.07	0.23	4.24	0.52	24.33	15.32	27.42	12.10	2.90
		2001	3.19	3.28	3.02	0.72	0.96	0.20	0.34	3.82	0.84	24.48	16.59	27.40	10.81	2.96
		2002	3.02	3.11	2.90	0.79	0.99	0.15	0.28	4.00	0.82	24.10	16.34	27.39	11.05	2.91
		2003	3.10	3.33	3.02	0.67	0.91	0.19	0.30	3.97	0.73	24.17	16.08	27.37	11.29	2.75
		2004	3.17	3.14	2.93	0.80	0.96	0.13	0.28	3.64	1.01	24.51	17.09	27.45	10.36	2.80
		2005	3.44	3.09	2.96	0.75	0.90	0.21	0.30	3.40	1.07	24.58	17.18	27.43	10.25	2.75
		2006	3.31	3.20	2.97	0.73	0.87	0.18	0.27	3.43	1.05	24.62	16.69	27.42	10.74	2.66
		2007	3.28	3.01	2.87	0.74	0.94	0.14	0.35	3.60	1.18	24.57	17.44	27.46	10.02	2.78
		2008	3.35	3.19	2.93	0.79	0.91	0.14	0.26	3.58	1.27	24.67	17.18	27.52	10.34	2.75
		2009	3.33	3.13	2.87	0.79	0.93	0.13	0.33	3.47	1.21	24.70	17.08	27.51	10.43	2.82
		2010	3.11	3.12	2.92	0.79	0.96	0.14	0.25	3.81	1.08	24.55	16.91	27.50	10.59	2.89
		2011	3.27	3.04	2.91	0.76	0.91	0.15	0.25	3.58	1.14	24.74	17.18	27.47	10.28	2.79
		2012	3.13	3.06	2.84	0.75	1.00	0.06	0.34	3.72	1.20	24.77	17.16	27.50	10.34	2.94
		2013	3.43	2.97	2.78	0.82	0.98	0.09	0.33	3.38	1.48	24.89	17.75	27.59	9.85	2.89
		2014	3.24	3.08	2.97	0.76	0.98	0.11	0.26	3.67	1.01	24.78	17.51	27.55	10.04	2.89
		2015	3.02	3.23	3.04	0.72	0.90	0.16	0.24	3.79	0.67	24.53	16.18	27.39	11.21	2.75
		2016	3.69	3.13	3.04	0.66	0.86	0.24	0.33	3.14	1.17	24.80	17.64	27.43	9.79	2.71
		2017	3.40	3.05	2.95	0.78	0.96	0.13	0.30	3.41	1.13	24.85	17.80	27.55	9.75	2.88
		2018	3.72	2.84	2.80	0.81	0.99	0.12	0.29	3.29	1.68	25.06	18.70	27.69	8.99	2.99
		2019	3.32	3.00	2.85	0.82	0.97	0.08	0.27	3.48	1.34	24.90	17.45	27.57	10.12	2.89
FA	UL	1982	2.90	3.26	2.88	0.79	0.94	0.11	0.29	3.80	0.86	24.45	15.72	27.37	11.65	2.85
		1983	3.41	2.83	2.70	0.78	1.00	0.11	0.51	3.14	1.56	24.92	17.84	27.49	9.65	2.87
		1984	3.28	2.74	2.76	0.73	1.00	0.22	0.52	3.36	1.18	24.65	17.30	27.51	10.21	2.88
		1985	2.92	3.12	2.79	0.72	0.99	0.14	0.47	3.86	0.86	24.41	15.93	27.33	11.40	2.95
		1986	2.73	2.95	2.64	0.90	1.00	0.03	0.31	3.97	1.06	24.58	16.16	27.30	11.14	2.98
		1987	2.86	2.99	2.79	0.87	1.00	0.10	0.33	3.90	0.92	24.49	15.71	27.45	11.74	2.92
		1988	2.55	3.28	2.88	0.81	1.00	0.13	0.28	4.28	0.51	24.04	13.64	27.51	13.87	2.86
		1989	2.64	3.28	2.89	0.86	1.00	0.11	0.27	4.20	0.55	24.13	13.87	27.59	13.72	2.88
		1990	2.75	3.05	2.85	0.90	1.00	0.08	0.24	4.01	0.63	24.44	15.48	27.54	12.07	2.92
		1991	2.42	3.39	2.86	0.94	1.00	0.03	0.16	4.44	0.46	24.10	13.79	27.42	13.64	2.97
		1992	2.34	3.37	2.98	0.87	0.97	0.08	0.15	4.59	0.20	24.01	13.36	27.34	13.97	2.89
		1993	2.38	3.52	3.01	0.84	0.95	0.13	0.14	4.57	0.16	23.81	12.39	27.49	15.10	2.81
		1994	2.38	3.69	3.00	0.92	0.95	0.07	0.08	4.56	0.24	23.85	12.30	27.48	15.18	2.88
		1995	2.37	3.60	2.95	0.90	0.99	0.08	0.16	4.56	0.30	23.84	12.47	27.53	15.06	2.92
		1996	2.42	3.41	2.93	0.86	0.99	0.12	0.21	4.49	0.33	23.89	12.62	27.54	14.91	2.87
		1997	2.34	3.49	2.95	0.89	0.99	0.09	0.16	4.59	0.27	23.87	12.72	27.52	14.80	2.90
		1998	2.46	3.58	2.98	0.80	0.95	0.11	0.22	4.44	0.32	23.94	12.95	27.41	14.46	2.87
		1999	2.56	3.45	2.99	0.80	0.96	0.14	0.23	4.35	0.37	24.03	13.48	27.44	13.97	2.84
		2000	2.50	3.56	2.99	0.87	0.98	0.10	0.18	4.40	0.32	23.97	13.45	27.48	14.03	2.96
		2001	2.65	3.45	2.98	0.83	0.96	0.13	0.21	4.20	0.45	24.07	13.59	27.51	13.92	2.86
		2002	2.37	3.53	2.99	0.81	0.99	0.12	0.22	4.55	0.20	23.85	12.76	27.48	14.72	2.90
		2003	2.85	3.57	3.14	0.72	0.94	0.23	0.27	4.07	0.25	24.05	14.43	27.34	12.92	2.96
		2004	2.63	3.51	3.03	0.85	0.99	0.10	0.21	4.24	0.37	24.14	14.19	27.48	13.29	3.00
		2005	2.52	3.50	2.95	0.90	0.99	0.06	0.19	4.35	0.43	24.07	13.47	27.56	14.09	2.94
		2006	2.61	3.39	2.94	0.84	0.99	0.10	0.26	4.26	0.49	24.12	13.80	27.53	13.73	2.88
		2007	2.48	3.46	3.00	0.86	0.98	0.10	0.19	4.43	0.28	24.03	13.30	27.49	14.19	2.91
		2008	2.75	3.43	2.97	0.84	0.96	0.10	0.23	4.07	0.55	24.26	14.33	27.50	13.18	2.89
		2009	2.59	3.45	2.93	0.89	0.99	0.08	0.21	4.24	0.49	24.09	13.46	27.62	14.16	2.90
		2010	2.44	3.23	2.97	0.76	0.99	0.23	0.27	4.47	0.20	23.72	12.71	27.57	14.86	2.74
		2011	2.70	3.46	3.00	0.84	0.94	0.10	0.19	4.13	0.45	24.18	14.00	27.48	13.48	2.87
		2012	2.66	3.38	3.01	0.84	0.98	0.09	0.19	4.10	0.41	24.26	14.25	27.59	13.33	2.88
		2013	2.59	3.43	2.97	0.88	1.00	0.08	0.19	4.23	0.42	24.14	13.69	27.63	13.93	2.92
		2014	2.38	3.56	3.02	0.91	1.00	0.04	0.10	4.48	0.24	24.05	13.25	27.55	14.31	2.96
		2015	2.33	3.60	2.98	0.90	1.00	0.05	0.11	4.56	0.24	23.92	12.97	27.53	14.56	2.96
		2016	2.56	3.59	3.01	0.85	0.95	0.09	0.16	4.28	0.34	24.03	13.31	27.52	14.22	2.86
		2017	2.52	3.59	2.99	0.90	0.99	0.05	0.15	4.31	0.38	24.09	13.57	27.58	14.01	2.96
		2018	2.59	3.54	2.99	0.85	0.99	0.09	0.21	4.28	0.41	24.06	13.73	27.56	13.83	2.95
		2019	2.59	3.48	3.01	0.86	0.99	0.07	0.19	4.21	0.40	24.19	14.47	27.50	13.03	3.01
FA	LL	1982	2.85	3.14	2.91	0.78	0.97	0.12	0.27	3.79	0.63	24.48	15.56	27.47	11.91	2.84
		1983	2.93	2.98	2.89	0.76	0.99	0.14	0.34	3.67	0.81	24.61	16.47	27.50	11.04	2.88
		1984	3.25	2.81	2.87	0.76	0.98	0.20	0.42	3.38	0.99	24.53	17.44	27.48	10.03	2.84
		1985	3.02	2.91	2.87	0.81	0.99	0.10	0.36	3.65	0.89	24.76	16.88	27.43	10.55	2.94
		1986	2.91	2.88	2.86	0.78	0.97	0.16	0.33	3.85	0.77	24.54	15.72	27.38	11.66	2.81
		1987	2.65	2.98	2.92	0.85	1.00	0.09	0.22	4.08	0.52	24.52	15.84	27.42	11.58	2.91
		1988	2.95	2.95	2.89	0.80	0.96	0.13	0.31	3.73	0.81	24.66	16.33	27.42	11.09	2.85
		1989	2.95	3.01	2.89	0.79	0.96	0.13	0.31	3.72	0.78	24.58	15.84	27.46	11.62	2.83
		1990	2.93	2.90	2.79	0.87	1.00	0.08	0.32	3.76	0.98	24.66	16.19	27.51	11.31	2.91
		1991	2.52	3.21	2.86	0.89	1.00	0.06	0.22	4.29	0.65	24.33	14.99	27.44	12.45	2.94
		1992	2.64	3.20	2.88	0.90	0.98	0.07	0.22	4.14	0.65	24.29	14.95	27.47	12.51	2.92
		1993	2.79	3.21	2.91	0.85	1.00	0.10	0.28	3.98	0.66	24.41	15.37	27.49	12.12	2.95
		1994	2.74	3.23	2.87	0.88	1.00	0.06	0.27	4.02	0.73	24.42	15.11	27.49	12.38	2.94
		1995	2.73	3.24	2.85	0.91	1.00	0.04	0.25	4.00	0.77	24.42	15.08	27.50	12.42	2.96
		1996	2.69	3.22	2.89	0.84	1.00	0.07	0.27	3.99	0.67	24.38	15.16	27.52	12.36	2.93
		1997	2.47	3.24	2.92	0.81	1.00	0.06	0.25	4.35	0.42	24.30	15.07	27.36	12.29	2.95
		1998	2.61	3.18	2.95	0.85	0.98	0.09	0.20	4.21	0.43	24.32	14.92	27.43	12.51	2.89
		1999	2.80	3.17	2.94	0.81	0.99	0.11	0.28	3.88	0.60	24.49	15.80	27.39	11.60	2.95
		2000	2.74	3.10	2.90	0.84	0.99	0.07	0.28	3.99	0.70	24.54	15.81	27.41	11.59	2.94
		2001	2.70	3.08	2.91	0.86	0.99	0.08	0.22	4.10	0.60	24.49	15.15	27.44	12.29	2.91
		2002	2.77	3.06	2.98	0.76	0.98	0.14	0.29	3.98	0.49	24.46	15.71	27.43	11.72	2.87
		2003	2.72	3.02	3.01	0.72	1.00	0.16	0.30	3.94	0.41	24.48	15.86	27.49	11.63	2.86
		2004	2.80	3.00	2.91	0.76	1.00	0.05	0.35	3.76	0.76	24.79	16.85	27.41	10.56	2.92
		2005	2.99	2.98	2.89	0.83	1.00	0.04	0.32	3.49	0.92	24.94	17.50	27.52	10.02	2.93
		2006	3.07	2.91	2.95	0.75	0.94	0.18	0.32	3.58	0.75	24.67	16.36	27.52	11.16	2.75
		2007	2.73	3.04	2.89	0.85	0.99	0.05	0.24	3.98	0.72	24.65	15.89	27.46	11.57	2.93
		2008	2.80	3.10	2.97	0.83	0.99	0.07	0.25	3.95	0.55	24.58	15.91	27.41	11.51	2.92
		2009	3.09	3.06	3.00	0.75	0.98	0.13	0.34	3.49	0.73	24.78	16.81	27.53	10.72	2.86
		2010	2.83	2.76	2.90	0.73	0.98	0.22	0.35	3.83	0.54	23.85	15.69	27.55	11.86	2.62
		2011	3.14	3.04	2.89	0.79	0.96	0.11	0.35	3.55	0.98	24.70	16.52	27.50	10.98	2.86
		2012	3.27	2.89	2.78	0.85	0.99	0.04	0.38	3.16	1.35	25.07	17.87	27.56	9.69	2.92
		2013	3.18	2.96	2.80	0.85	0.99	0.06	0.39	3.36	1.21	24.86	16.95	27.57	10.63	2.90
		2014	3.22	2.90	2.80	0.88	0.98	0.07	0.34	3.39	1.20	24.87	16.95	27.57	10.62	2.90
		2015	2.70	3.20	2.91	0.84	0.99	0.06	0.25	4.05	0.68	24.43	15.30	27.49	12.19	2.92
		2016	2.90	3.04	2.83	0.83	0.98	0.07	0.28	3.74	1.03	24.68	16.21	27.51	11.30	2.90
		2017	2.97	3.06	2.85	0.86	1.00	0.05	0.32	3.63	0.95	24.76	16.38	27.58	11.19	2.92
		2018	3.08	3.05	2.91	0.82	1.00	0.11	0.34	3.59	0.88	24.70	16.90	27.51	10.61	2.98
		2019	3.05	2.90	2.84	0.86	0.98	0.05	0.31	3.48	1.06	24.93	17.26	27.53	10.27	2.91

## 2 Trait ANOVA results for decade and season

### 2.1 Trophic level

**TABLE A3:** ANOVA results for Trophic level. Standard ANOVA table for the model of Trophic level – Decade and Season, showing degrees of freedom, sum of squares, mean squared error, *F*‐statistic, and *p*‐value for the given factor

	Degrees of freedom	Sum of squares	Mean squared error	*F*‐value	Pr(>*F*)
Decade	3	0.587	0.1957	14.87	2.4 × 10^−9^
Season	1	1.025	1.0246	77.84	<2.0 × 10^−16^
Residuals	595	7.832	7.832		

### 2.2 Maximum temperature

**TABLE A4:** ANOVA results for Maximum Temperature. Standard ANOVA table for the model of Maximum Temperature – Decade and Season, showing degrees of freedom, sum of squares, mean squared error, *F*‐statistic, and *p*‐value for the given factor

	Degrees of freedom	Sum of squares	Mean squared error	*F*‐value	Pr(>*F*)
Decade	3	0.874	0.2914	23.31	2.82 × 10^−14^
Season	1	0.399	0.3991	31.92	2.49 × 10^−8^
Residuals	595	7.439	0.0125		

### 2.3 Piscivore

**TABLE A5:** ANOVA results for Piscivore. Standard ANOVA table for the model of Piscivore – Decade and Season, showing degrees of freedom, sum of squares, mean squared error, *F*‐statistic, and *p*‐value for the given factor

	Degrees of freedom	Sum of squares	Mean squared error	*F*‐value	Pr(>*F*)
Decade	3	0.304	0.1013	13.32	2.0 × 10^−8^
Season	1	1.992	1.9921	261.88	<2 × 10^−16^
Residuals	595	4.526	0.0076		

### 2.4 Age at maturity

**TABLE A6:** ANOVA results for Age at maturity. Standard ANOVA table for the model of Age at maturity – Decade and Season, showing degrees of freedom, sum of squares, mean squared error, *F*‐statistic, and *p*‐value for the given factor

	Degrees of freedom	Sum of squares	Mean squared error	*F*‐value	Pr(>*F*)
Decade	3	1.07	0.36	7.075	.0001
Season	1	36.18	36.18	717.707	<2.0 × 10^−16^
Residuals	595	29.99	0.05		

### 2.5 Natural mortality

**TABLE A7:** ANOVA results for Natural mortality. Standard ANOVA table for the model of Natural mortality – Decade and Season, showing degrees of freedom, sum of squares, mean squared error, *F*‐statistic, and *p*‐value for the given factor

	Degrees of freedom	Sum of squares	Mean squared error	*F*‐value	Pr(>*F*)
Decade	3	0.0350	0.0117	8.561	1.43 × 10^−5^
Season	1	0.7662	0.7662	562.561	<2.0 × 10^−16^
Residuals	595	0.8104	0.0014		

### 2.6 Maximum age

**TABLE A8:** ANOVA results for Maximum age. Standard ANOVA table for the model of Maximum age – Decade and Season, showing degrees of freedom, sum of squares, mean squared error, *F*‐statistic, and *p*‐value for the given factor

	Degrees of freedom	Sum of squares	Mean squared error	*F*‐value	Pr(>*F*)
Decade	3	454	151.4	9.496	3.90 × 10^−9^
Season	1	357	357.2	22.396	2.77 × 10^−6^
Residuals	595	9489	15.9		

### 2.7 Asymptotic length (L_∞_)

**TABLE A9:** ANOVA results for L_∞_. Standard ANOVA table for the model of L infinity – Decade and Season, showing degrees of freedom, sum of squares, mean squared error, *F*‐statistic, and *p*‐value for the given factor

	Degrees of freedom	Sum of squares	Mean squared error	*F*‐value	Pr(>*F*)
Decade	3	742,723	247,574	10.97	5.08 × 10^−7^
Season	1	310,067	310,067	13.74	.0002
Residuals	595	13,426,370	22,565		

### 2.8 Generation time

**TABLE A10:** ANOVA results for Generation time. Standard ANOVA table for the model of Generation time – Decade and Season, showing degrees of freedom, sum of squares, mean squared error, *F*‐statistic, and *p*‐value for the given factor

	Degrees of freedom	Sum of squares	Mean squared error	*F*‐value	Pr(>*F*)
Decade	3	48.6	16.18	8.38	1.83 × 10^−5^
Season	1	38.9	38.85	20.12	8.73 × 10^−6^
Residuals	595	1149.0	1.93		

### 2.9 Parental care

**TABLE A11:** ANOVA results for Parental care. Standard ANOVA table for the model of Parental care – Decade and Season, showing degrees of freedom, sum of squares, mean squared error, *F*‐statistic, and *p*‐value for the given factor

	Degrees of freedom	Sum of squares	Mean squared error	*F*‐value	Pr(>*F*)
Decade	3	4.38	1.46	7.05	.0001
Season	1	39.49	39.49	190.61	<2.0 × 10^−16^
Residuals	595	123.29	0.21		

**TABLE A12:** Species catch data by time period. Total catch in number of individuals by species for all species included in the functional diversity analyses. Data are shown for two time periods (1982–2000 and 2001–2019). A column for occurrence is included to indicate whether species are temperate, subtropical, or tropical

Species (Latin name)	Species (common name)	Occurrence	Total Catch (1982–2000)	Total Catch (2001–2019)	Total Catch All Years
*Pogonias cromis*	Black drum	Subtropical	182,521	246,787	429,308
*Ariopsis felis*	Hardhead catfish	Subtropical	214,439	207,623	422,062
*Sciaenops ocellatus*	Red drum	Subtropical	141,312	174,659	315,971
*Cynoscion nebulosus*	Spotted seatrout	Subtropical	99,881	118,421	218,302
*Dorosoma cepedianum*	Gizzard shad	Subtropical	94,643	95,078	189,721
*Bagre marinus*	Gafftopsail catfish	Subtropical	31,699	90,997	122,696
*Brevoortia patronus*	Gulf menhaden	Subtropical	41,174	52,287	93,461
*Mugil cephalus*	Striped mullet	Subtropical	50,358	43,103	93,461
*Micropogonias undulatus*	Atlantic croaker	Subtropical	35,077	32,844	67,921
*Elops saurus*	Ladyfish	Subtropical	10,179	37,250	47,429
*Leiostomus xanthurus*	Spot	Subtropical	21,111	25,369	46,480
*Atractosteus spatula*	Alligator gar	Subtropical	14,046	24,347	38,393
*Archosargus probatocephalus*	Sheepshead	Subtropical	15,529	20,020	35,549
*Lepisosteus oculatus*	Spotted gar	Subtropical	12,670	13,979	26,649
*Paralichthys lethostigma*	Southern flounder	Subtropical	10,738	6647	17,385
*Ictalurus furcatus*	Blue catfish	Subtropical	5203	7801	13,004
*Carcharhinus leucas*	Bull shark	Subtropical	3759	6216	9975
*Lagodon rhomboides*	Pinfish	Subtropical	2399	4472	6871
*Sphyrna tiburo*	Bonnethead	Subtropical	1174	4174	5348
*Lutjanus griseus*	Gray snapper	Subtropical	1052	3943	4995
*Brevoortia gunteri*	Finescale menhaden	Subtropical	2491	2164	4655
*Rhinoptera bonasus*	Cownose ray	Tropical	1559	3075	4634
*Cynoscion arenarius*	Sand seatrout	Subtropical	1768	2778	4546
*Ictiobus bubalus*	Smallmouth buffalo	Temperate	2450	1848	4298
*Carcharhinus limbatus*	Blacktip shark	Subtropical	1387	2260	3647
*Chaetodipterus faber*	Atlantic spadefish	Subtropical	1067	1613	2680
*Trachinotus carolinus*	Florida pompano	Subtropical	723	1871	2594
*Lepisosteus osseus*	Longnose gar	Subtropical	1085	1431	2516
*Scomberomorus maculatus*	Spanish mackerel	Subtropical	1018	1468	2486
*Menticirrhus americanus*	Southern kingfish	Subtropical	307	1762	2069
*Dasyatis sabina*	Atlantic stingray	Subtropical	793	784	1577
*Orthopristis chrysoptera*	Pigfish	Temperate	725	608	1333
*Dorosoma petenense*	Threadfin shad	Subtropical	515	714	1229
*Paralichthys albigutta*	Gulf flounder	Subtropical	879	313	1192
*Cyprinus carpio*	Common carp	Subtropical	868	230	1098
*Carcharhinus brevipinna*	Spinner shark	Subtropical	113	849	962
*Lobotes surinamensis*	Atlantic tripletail	Subtropical	488	449	937
*Bairdiella chrysoura*	Silver perch	Subtropical	378	544	922
*Caranx hippos*	Crevalle jack	Subtropical	323	591	914
*Centropomus undecimalis*	Common snook	Tropical	213	667	880
*Peprilus paru*	Harvestfish	Subtropical	338	388	726
*Rhizoprionodon terraenovae*	Atlantic sharpnose shark	Subtropical	339	373	712
*Sphyrna lewini*	Scalloped hammerhead	Tropical	134	409	543
*Morone mississippiensis*	Yellow bass	Subtropical	257	272	529
*Carcharhinus isodon*	Finetooth shark	Subtropical	241	280	521
*Megalops atlanticus*	Tarpon	Subtropical	146	271	417
*Pomatomus saltatrix*	Bluefish	Subtropical	221	196	417
*Lepisosteus platostomus*	Shortnose gar	Subtropical	292	32	324
*Prionotus tribulus*	Bighead searobin	Subtropical	120	194	314
*Alosa chrysochloris*	Skipjack herring	Subtropical	173	136	309
*Opsanus beta*	Gulf toadfish	Subtropical	99	100	199
*Trachinotus falcatus*	Permit	Subtropical	53	145	198
*Negaprion brevirostris*	Lemon shark	Subtropical	178	14	192
*Selene vomer*	Lookdown	Subtropical	35	130	165
*Menticirrhus littoralis*	Gulf kingfish	Subtropical	34	112	146
*Peprilus burti*	Gulf butterfish	Subtropical	48	83	131
*Polydactylus octonemus*	Atlantic threadfin	Subtropical	84	31	115
*Morone chrysops*	White bass	Temperate	68	39	107
*Trinectes maculatus*	Hogchoker	Subtropical	70	27	97
*Mugil curema*	White mullet	Subtropical	61	34	95
*Dasyatis americana*	Southern stingray	Subtropical	25	65	90
*Ictalurus punctatus*	Channel catfish	Subtropical	25	45	70
*Chloroscombrus chrysurus*	Atlantic bumper	Subtropical	2	67	69
*Morone saxatilis*	Striped bass	Temperate	44	24	68
*Micropterus salmoides*	Largemouth bass	Subtropical	40	25	65
*Gymnura micrura*	Smooth butterfly ray	N/A^a^	2	61	63
*Ancylopsetta quadrocellata*	Ocellated flounder	Subtropical	17	43	60
*Centropomus parallelus*	Smallscale fat snook	Subtropical	15	45	60
*Aplodinotus grunniens*	Freshwater drum	Subtropical	20	39	59
*Carcharhinus obscurus*	Dusky shark	Subtropical	10	48	58
*Cynoscion nothus*	Silver seatrout	Subtropical	17	38	55
*Pylodictis olivaris*	Flathead catfish	Subtropical	25	23	48
*Synodus foetens*	Inshore lizardfish	Tropical	28	16	44
*Syngnathus scovelli*	Gulf pipefish	Tropical	1	41	42
*Echeneis naucrates*	Sharksucker	Subtropical	7	29	36
*Remora Shark sucker*	Remora	Subtropical	3	33	36
*Strongylura marina*	Atlantic needlefish	Subtropical	19	15	34
*Diapterus auratus*	Irish pompano	Tropical	3	29	32
*Syngnathus louisianae*	Chain pipefish	Subtropical	2	30	32
*Prionotus rubio*	Blackwing searobin	Subtropical	11	19	30
*Carcharhinus plumbeus*	Sandbar shark	Subtropical	7	20	27
*Oreochromis aureus*	Blue tilapia	Tropical	1	26	27
*Rachycentron canadum*	Cobia	Subtropical	12	14	26
*Eucinostomus gula*	Silver jenny	Subtropical	24	1	25
*Gobiosoma bosc*	Naked goby	Tropical	2	20	22
*Mycteroperca microlepis*	Gag	Subtropical	5	16	21
*Trichiurus lepturus*	Atlantic cutlassfish	Subtropical	10	11	21
*Gobiesox strumosus*	Skilletfish	Tropical	2	18	20
*Anchoa mitchilli*	Bay anchovy	Subtropical	1	17	18
*Pterygoplichthys anisitsi*	Parana sailfin catfish	Tropical	0	18	18
*Chilomycterus schoepfi*	Striped burrfish	Tropical	3	14	17
*Histrio histrio*	Sargassum fish	Subtropical	1	15	16
*Oligoplites saurus*	Leatherjack	Subtropical	6	10	16
*Prionotus longispinosus*	Bigeye searobin	Tropical	8	7	15
*Hemicaranx amblyrhynchus*	Bluntnose jack	Subtropical	6	8	14
*Syacium gunteri*	Shoal flounder	Tropical	0	14	14
*Selene setapinnis*	Atlantic moonfish	Subtropical	4	9	13
*Amia calva*	Bowfin	Subtropical	7	5	12
*Antennarius striatus*	Striated frogfish	Subtropical	0	12	12
*Fundulus chrysotus*	Golden topminnow	Subtropical	12	0	12
*Scorpaena plumieri*	Spotted scorpionfish	Subtropical	0	12	12
*Dasyatis say*	Bluntnose stingray	Subtropical	2	9	11
*Lepomis macrochirus*	Bluegill	Subtropical	7	3	10
*Menidia peninsulae*	Tidewater silverside	Tropical	0	10	10
*Sphyraena barracuda*	Great barracuda	Subtropical	1	9	10
*Sphyrna mokarran*	Great hammerhead	Subtropical	6	4	10
*Syngnathus pelagicus*	Sargassum pipefish	Subtropical	0	10	10
*Achirus lineatus*	Lined sole	Tropical	6	3	9
*Lepomis microlophus*	Redear sunfish	Subtropical	5	4	9
*Ctenopharyngodon idella*	Grass carp	Subtropical	5	3	8
*Larimus fasciatus*	Banded drum	Subtropical	5	3	8
*Stellifer lanceolatus*	Star drum	Subtropical	1	7	8
*Gobiomorus dormitor*	Bigmouth sleeper	Tropical	2	5	7
*Anchoa hepsetus*	Striped anchovy	Subtropical	4	2	6
*Kyphosus saltatrix*	Bermuda chub	Subtropical	1	5	6
*Lutjanus synagris*	Lane snapper	Subtropical	3	3	6
*Aetobatus narinari*	Spotted eagle ray	Subtropical	1	4	5
*Caranx latus*	Horse‐eye jack	Subtropical	1	4	5
*Carcharhinus falciformis*	Silky shark	Subtropical	2	3	5
*Lepomis gulosus*	Warmouth	Temperate	1	4	5
*Trachinotus goodei*	Palometa	Subtropical	5	0	5
*Carcharhinus acronotus*	Blacknose shark	Subtropical	1	3	4
*Citharichthys spilopterus*	Bay whiff	Tropical	0	4	4
*Echeneis neucratoides*	Whitefin sharksucker	Subtropical	0	4	4
*Gobiosoma robustum*	Code goby	Tropical	0	4	4
*Harengula jaguana*	Scaled sardine	Tropical	1	3	4
*Saurida caribbaea*	Smallscale lizardfish	Tropical	1	3	4
*Scomberomorus cavalla*	King mackerel	Tropical	2	2	4
*Ameiurus melas*	Black bullhead	Temperate	0	3	3
*Ameiurus natalis*	Yellow bullhead	Temperate	2	1	3
*Carcharhinus porosus*	Smalltail shark	Subtropical	2	1	3
*Cyclopsetta chittendeni*	Mexican flounder	Subtropical	0	3	3
*Dasyatis centroura*	Roughtail stingray	Subtropical	1	2	3
*Gerres cinereus*	Yellowfin mojarra	Subtropical	0	3	3
*Ophichthus gomesi*	Shrimp eel	Tropical	2	1	3
*Pomoxis annularis*	White crappie	Temperate	2	1	3
*Scomberomorus regalis*	Cero	Tropical	1	2	3
*Sphyrna tudes*	Smalleye hammerhead	Subtropical	3	0	3
		Totals	1,011,608	1,246,439	2,258,047

^a^ N/A Smooth butterfly ray (*Gymnura micrura*) did not have occurrence data available on FishBase.

**TABLE A13:** Proportion of total catch by species. The proportion of the total catch in numbers by species for two time periods (1982–2000 and 2001–2019). A column for occurrence is included to indicate whether species are temperate, subtropical, or tropical

Species (Latin name)	Species (common name)	Occurrence	Catch proportion (1982–2000)	Catch proportion (2001–2019)
*Pogonias cromis*	Black drum	Subtropical	0.1804266079	0.1979936443
*Ariopsis felis*	Hardhead catfish	Subtropical	0.2119783553	0.1665729330
*Sciaenops ocellatus*	Red drum	Subtropical	0.1396904730	0.1401263921
*Cynoscion nebulosus*	Spotted seatrout	Subtropical	0.0987348854	0.0950074572
*Dorosoma cepedianum*	Gizzard shad	Subtropical	0.0935569905	0.0762797056
*Bagre marinus*	Gafftopsail catfish	Subtropical	0.0313352603	0.0730055783
*Brevoortia patronus*	Gulf menhaden	Subtropical	0.0407015366	0.0419491046
*Mugil cephalus*	Striped mullet	Subtropical	0.0497801520	0.0345809141
*Micropogonias undulatus*	Atlantic croaker	Subtropical	0.0346744984	0.0263502666
*Elops saurus*	Ladyfish	Subtropical	0.0100621980	0.0298851368
*Leiostomus xanthurus*	Spot	Subtropical	0.0208687555	0.0203531821
*Atractosteus spatula*	Alligator gar	Subtropical	0.0138848250	0.0195332463
*Archosargus probatocephalus*	Sheepshead	Subtropical	0.0153508078	0.0160617567
*Lepisosteus oculatus*	Spotted gar	Subtropical	0.0125246143	0.0112151497
*Paralichthys lethostigma*	Southern flounder	Subtropical	0.0106147836	0.0053327921
*Ictalurus furcatus*	Blue catfish	Subtropical	0.0051432966	0.0062586296
*Carcharhinus leucas*	Bull shark	Subtropical	0.0037158662	0.0049870070
*Lagodon rhomboides*	Pinfish	Subtropical	0.0023714720	0.0035878210
*Sphyrna tiburo*	Bonnethead	Subtropical	0.0011605286	0.0033487399
*Lutjanus griseus*	Gray snapper	Subtropical	0.0010399285	0.0031634119
*Brevoortia gunteri*	Finescale menhaden	Subtropical	0.0024624163	0.0017361459
*Rhinoptera bonasus*	Cownose ray	Tropical	0.0015411108	0.0024670281
*Cynoscion arenarius*	Sand seatrout	Subtropical	0.0017477126	0.0022287493
*Ictiobus bubalus*	Smallmouth buffalo	Temperate	0.0024218867	0.0014826237
*Carcharhinus limbatus*	Blacktip shark	Subtropical	0.0013710845	0.0018131653
*Chaetodipterus faber*	Atlantic spadefish	Subtropical	0.0010547564	0.0012940866
*Trachinotus carolinus*	Florida pompano	Subtropical	0.0007147037	0.0015010763
*Lepisosteus osseus*	Longnose gar	Subtropical	0.0010725498	0.0011480706
*Scomberomorus maculatus*	Spanish mackerel	Subtropical	0.0010063187	0.0011777552
*Menticirrhus americanus*	Southern kingfish	Subtropical	0.0003034772	0.0014136271
*Dasyatis sabina*	Atlantic stingray	Subtropical	0.0007839005	0.0006289919
*Orthopristis chrysoptera*	Pigfish	Temperate	0.0007166808	0.0004877896
*Dorosoma petenense*	Threadfin shad	Subtropical	0.0005090905	0.0005728319
*Paralichthys albigutta*	Gulf flounder	Subtropical	0.0008689137	0.0002511154
*Cyprinus carpio*	Common carp	Subtropical	0.0008580399	0.0001845257
*Carcharhinus brevipinna*	Spinner shark	Subtropical	0.0001117033	0.0006811404
*Lobotes surinamensis*	Atlantic tripletail	Subtropical	0.0004824003	0.0003602262
*Bairdiella chrysoura*	Silver perch	Subtropical	0.0003736625	0.0004364433
*Caranx hippos*	Crevalle jack	Subtropical	0.0003192936	0.0004741508
*Centropomus undecimalis*	Common snook	Tropical	0.0002105559	0.0005351245
*Peprilus paru*	Harvestfish	Subtropical	0.0003341215	0.0003112868
*Rhizoprionodon terraenovae*	Atlantic sharpnose shark	Subtropical	0.0003351100	0.0002992525
*Sphyrna lewini*	Scalloped hammerhead	Tropical	0.0001324624	0.0003281348
*Morone mississippiensis*	Yellow bass	Subtropical	0.0002540510	0.0002182217
*Carcharhinus isodon*	Finetooth shark	Subtropical	0.0002382346	0.0002246400
*Megalops atlanticus*	Tarpon	Subtropical	0.0001443247	0.0002174194
*Pomatomus saltatrix*	Bluefish	Subtropical	0.0002184641	0.0001572480
*Lepisosteus platostomus*	Shortnose gar	Subtropical	0.0002886494	0.0000256731
*Prionotus tribulus*	Bighead searobin	Subtropical	0.0001186230	0.0001556434
*Alosa chrysochloris*	Skipjack herring	Subtropical	0.0001710149	0.0001091108
*Opsanus beta*	Gulf toadfish	Subtropical	0.0000978640	0.0000802286
*Trachinotus falcatus*	Permit	Subtropical	0.0000523918	0.0001163314
*Negaprion brevirostris*	Lemon shark	Subtropical	0.0001759575	0.0000112320
*Selene vomer*	Lookdown	Subtropical	0.0000345984	0.0001042971
*Menticirrhus littoralis*	Gulf kingfish	Subtropical	0.0000336099	0.0000898560
*Peprilus burti*	Gulf butterfish	Subtropical	0.0000474492	0.0000665897
*Polydactylus octonemus*	Atlantic threadfin	Subtropical	0.0000830361	0.0000248709
*Morone chrysops*	White bass	Temperate	0.0000672197	0.0000312891
*Trinectes maculatus*	Hogchoker	Subtropical	0.0000691968	0.0000216617
*Mugil curema*	White mullet	Subtropical	0.0000603000	0.0000272777
*Dasyatis americana*	Southern stingray	Subtropical	0.0000247131	0.0000521486
*Ictalurus punctatus*	Channel catfish	Subtropical	0.0000247131	0.0000361028
*Chloroscombrus chrysurus*	Atlantic bumper	Subtropical	0.0000019771	0.0000537531
*Morone saxatilis*	Striped bass	Temperate	0.0000434951	0.0000192549
*Micropterus salmoides*	Largemouth bass	Subtropical	0.0000395410	0.0000200571
*Gymnura micrura*	Smooth butterfly ray	N/A^a^	0.0000019771	0.0000489394
*Ancylopsetta quadrocellata*	Ocellated flounder	Subtropical	0.0000168049	0.0000344983
*Centropomus parallelus*	Smallscale fat snook	Subtropical	0.0000148279	0.0000361028
*Aplodinotus grunniens*	Freshwater drum	Subtropical	0.0000197705	0.0000312891
*Carcharhinus obscurus*	Dusky shark	Subtropical	0.0000098853	0.0000385097
*Cynoscion nothus*	Silver seatrout	Subtropical	0.0000168049	0.0000304869
*Pylodictis olivaris*	Flathead catfish	Subtropical	0.0000247131	0.0000184526
*Synodus foetens*	Inshore lizardfish	Tropical	0.0000276787	0.0000128366
*Syngnathus scovelli*	Gulf pipefish	Tropical	0.0000009885	0.0000328937
*Echeneis naucrates*	Sharksucker	Subtropical	0.0000069197	0.0000232663
*Remora Small eye*	Remora	Subtropical	0.0000029656	0.0000264754
*Strongylura marina*	Atlantic needlefish	Subtropical	0.0000187820	0.0000120343
*Diapterus auratus*	Irish pompano	Tropical	0.0000029656	0.0000232663
*Syngnathus louisianae*	Chain pipefish	Subtropical	0.0000019771	0.0000240686
*Prionotus rubio*	Blackwing searobin	Subtropical	0.0000108738	0.0000152434
*Carcharhinus plumbeus*	Sandbar shark	Subtropical	0.0000069197	0.0000160457
*Oreochromis aureus*	Blue tilapia	Tropical	0.0000009885	0.0000208594
*Rachycentron canadum*	Cobia	Subtropical	0.0000118623	0.0000112320
*Eucinostomus gula*	Silver jenny	Subtropical	0.0000237246	0.0000008023
*Gobiosoma bosc*	Naked goby	Tropical	0.0000019771	0.0000160457
*Mycteroperca microlepis*	Gag	Subtropical	0.0000049426	0.0000128366
*Trichiurus lepturus*	Atlantic cutlassfish	Subtropical	0.0000098853	0.0000088251
*Gobiesox strumosus*	Skilletfish	Tropical	0.0000019771	0.0000144411
*Anchoa mitchilli*	Bay anchovy	Subtropical	0.0000009885	0.0000136389
*Pterygoplichthys anisitsi*	Parana sailfin catfish	Tropical	0.0000000000	0.0000144411
*Chilomycterus schoepfi*	Striped burrfish	Tropical	0.0000029656	0.0000112320
*Histrio Small eye*	Sargassum fish	Subtropical	0.0000009885	0.0000120343
*Oligoplites saurus*	Leatherjack	Subtropical	0.0000059312	0.0000080229
*Prionotus longispinosus*	Bigeye searobin	Tropical	0.0000079082	0.0000056160
*Hemicaranx amblyrhynchus*	Bluntnose jack	Subtropical	0.0000059312	0.0000064183
*Syacium gunteri*	Shoal flounder	Tropical	0.0000000000	0.0000112320
*Selene setapinnis*	Atlantic moonfish	Subtropical	0.0000039541	0.0000072206
*Amia calva*	Bowfin	Subtropical	0.0000069197	0.0000040114
*Antennarius striatus*	Striated frogfish	Subtropical	0.0000000000	0.0000096274
*Fundulus chrysotus*	Golden topminnow	Subtropical	0.0000118623	0.0000000000
*Scorpaena plumieri*	Spotted scorpionfish	Subtropical	0.0000000000	0.0000096274
*Dasyatis say*	Bluntnose stingray	Subtropical	0.0000019771	0.0000072206
*Lepomis macrochirus*	Bluegill	Subtropical	0.0000069197	0.0000024069
*Menidia peninsulae*	Tidewater silverside	Tropical	0.0000000000	0.0000080229
*Sphyraena barracuda*	Great barracuda	Subtropical	0.0000009885	0.0000072206
*Sphyrna mokarran*	Great hammerhead	Subtropical	0.0000059312	0.0000032091
*Syngnathus pelagicus*	Sargassum pipefish	Subtropical	0.0000000000	0.0000080229
*Achirus lineatus*	Lined sole	Tropical	0.0000059312	0.0000024069
*Lepomis microlophus*	Redear sunfish	Subtropical	0.0000049426	0.0000032091
*Ctenopharyngodon idella*	Grass carp	Subtropical	0.0000049426	0.0000024069
*Larimus fasciatus*	Banded drum	Subtropical	0.0000049426	0.0000024069
*Stellifer lanceolatus*	Star drum	Subtropical	0.0000009885	0.0000056160
*Gobiomorus dormitor*	Bigmouth sleeper	Tropical	0.0000019771	0.0000040114
*Anchoa hepsetus*	Striped anchovy	Subtropical	0.0000039541	0.0000016046
*Kyphosus saltatrix*	Bermuda chub	Subtropical	0.0000009885	0.0000040114
*Lutjanus synagris*	Lane snapper	Subtropical	0.0000029656	0.0000024069
*Aetobatus narinari*	Spotted eagle ray	Subtropical	0.0000009885	0.0000032091
*Caranx latus*	Horse‐eye jack	Subtropical	0.0000009885	0.0000032091
*Carcharhinus falciformis*	Silky shark	Subtropical	0.0000019771	0.0000024069
*Lepomis gulosus*	Warmouth	Temperate	0.0000009885	0.0000032091
*Trachinotus goodei*	Palometa	Subtropical	0.0000049426	0.0000000000
*Carcharhinus acronotus*	Blacknose shark	Subtropical	0.0000009885	0.0000024069
*Citharichthys spilopterus*	Bay whiff	Tropical	0.0000000000	0.0000032091
*Echeneis neucratoides*	Whitefin sharksucker	Subtropical	0.0000000000	0.0000032091
*Gobiosoma robustum*	Code goby	Tropical	0.0000000000	0.0000032091
*Harengula jaguana*	Scaled sardine	Tropical	0.0000009885	0.0000024069
*Saurida caribbaea*	Smallscale lizardfish	Tropical	0.0000009885	0.0000024069
*Scomberomorus cavalla*	King mackerel	Tropical	0.0000019771	0.0000016046
*Ameiurus melas*	Black bullhead	Temperate	0.0000000000	0.0000024069
*Ameiurus natalis*	Yellow bullhead	Temperate	0.0000019771	0.0000008023
*Carcharhinus porosus*	Smalltail shark	Subtropical	0.0000019771	0.0000008023
*Cyclopsetta chittendeni*	Mexican flounder	Subtropical	0.0000000000	0.0000024069
*Dasyatis centroura*	Roughtail stingray	Subtropical	0.0000009885	0.0000016046
*Gerres cinereus*	Yellowfin mojarra	Subtropical	0.0000000000	0.0000024069
*Ophichthus gomesi*	Shrimp eel	Tropical	0.0000019771	0.0000008023
*Pomoxis annularis*	White crappie	Temperate	0.0000019771	0.0000008023
*Scomberomorus regalis*	Cero	Tropical	0.0000009885	0.0000016046
*Sphyrna tudes*	Smalleye hammerhead	Subtropical	0.0000029656	0.0000000000

^a^ N/A Smooth butterfly ray (*Gymnura micrura*) did not have occurrence data available on FishBase.

**TABLE A14:** Proportion of total catch by occurrence. The proportion of the total catch by occurrence category (Temperate, Subtropical, and Tropical) for two time periods (1982–2000 and 2001–2019)

	Proportion of total catch	
	1982–2000	2001–2019
Temperate	0.0032542250	0.0020281779
Subtropical	0.9947973919	0.9944000469
Tropical	0.0019464061	0.0035228359
